# Supplementary material for: Comparative transcriptomic and co-expression analyses enable the discovery of key enzymes responsible for oleuropein biosynthesis in olive (Olea europaea)
Source: Plant Commun. 2026 Jan 8;7(4):101713. doi: 10.1016/j.xplc.2026.101713 (PMC13084073; doi:10.1016/j.xplc.2026.101713)
Supplement: Document S1. Supplemental Figures 1–20 [file mmc1.pdf]

Plant Communications, Volume *7*

Supplemental information

Comparative transcriptomic and co-expression analyses enable the discovery of key enzymes responsible for oleuropein biosynthesis in olive (*Olea europaea*)

Ornella Calderini, Mohamed O. Kamileen, Yoko Nakamura, Sarah Heinicke, Ryan M. Alam, Benke Hong, Yindi Jiang, Alma Gutiérrez-Vences, Fiammetta Alagna, Francesco Paolocci, Maria Cristina Valeri, Edoardo Franco, Soraya Mousavi, Roberto Mariotti, Lorenzo Caputi, Sarah E. O'Connor, and Carlos E. Rodríguez-López

SUPPLEMENTARY FIGURES

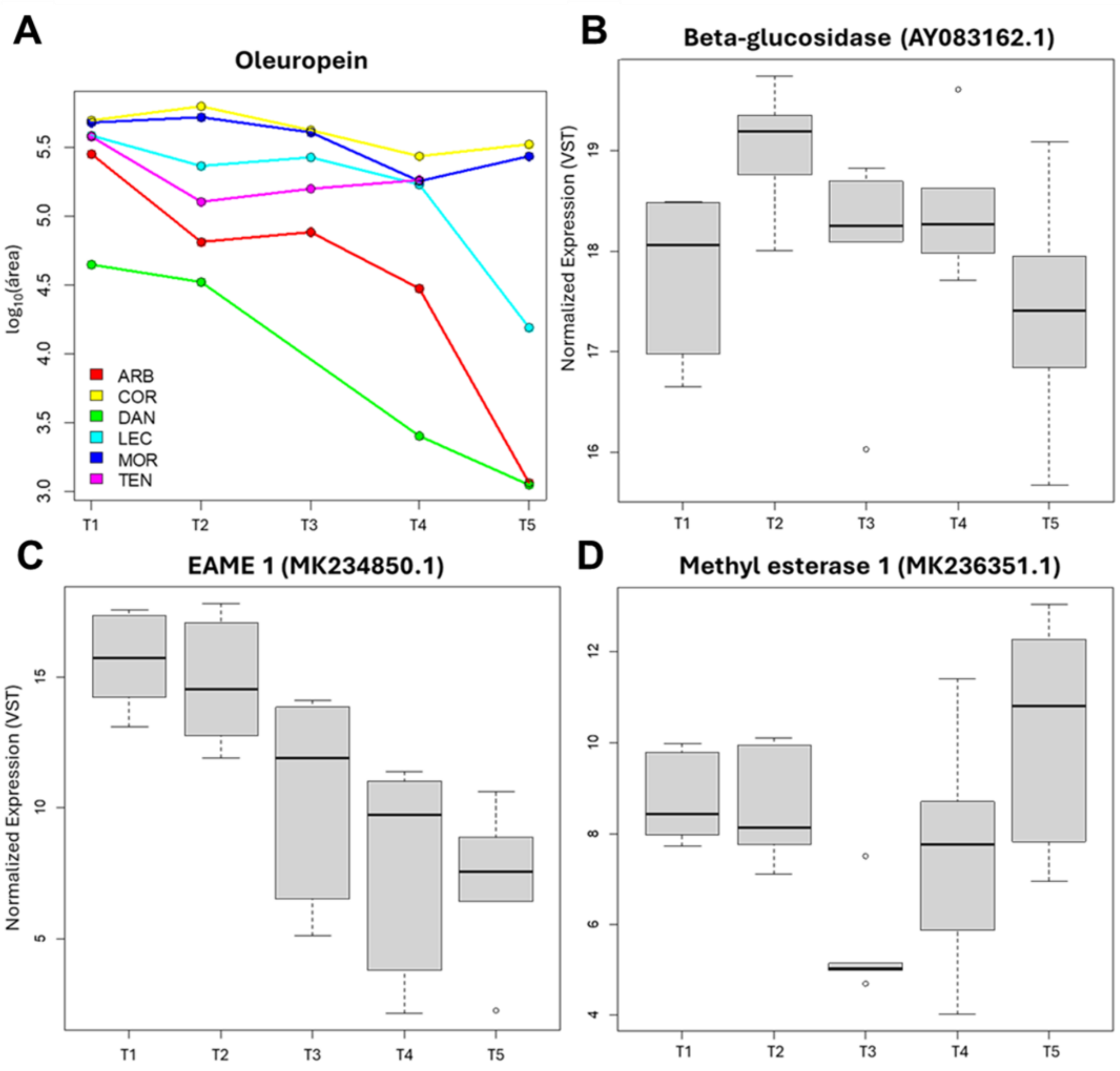

Figure S1. Oleuropein content and putative degradation pathway genes expression through olive maturation. (A) Logarithm of the area under the curve of the extracted ion chromatograms of the most abundant oleuropein adduct ($[M\text{-}H]^-$ = 539.1770 ± 0.05) of the cultivars *Dolce d'Andria* (DAN, green), *Tendellone* (TEN, purple), *Arbequina* (ARB, red), *Leccino* (LEC, cyan), *Coratina* (COR, yellow) and *Moraiolo* (MOR, blue). Boxplots depict the normalized mean expression after DESeq2 variance stabilized transformation of **(B)** a beta-glucosidase (GenBank: AY083162.1) reported by Koudounas *et al*. (2015), and **(C)** elenolic acid methylesterase 1 (EAME1, GenBank: MK234850.1) and **(D)** a methyl esterase 1 (GenBank: MK236351.1) reported by Volk *et al*. (2019), presumed to belong to degradation pathway of oleuropein and oleoside methyl ester.

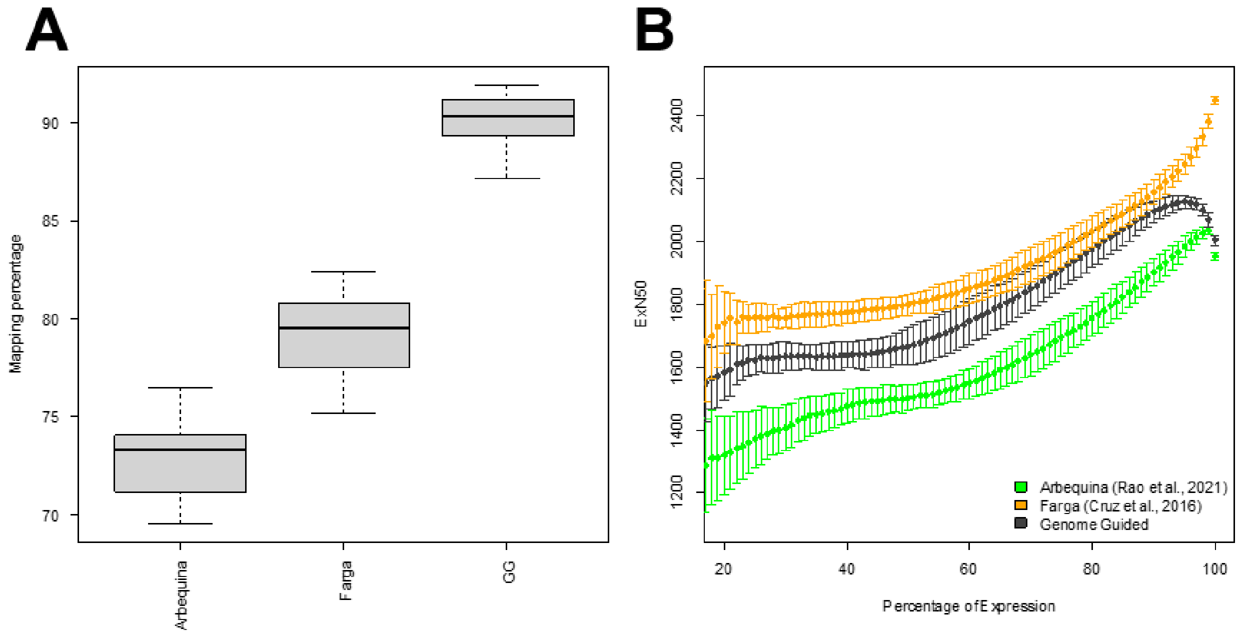

Figure S2. Assembly metrics. (A) Mapping of the sequenced reads to the published Arbequina (Rao *et al.*, 2021) and Farga (Cruz *et al.*, 2016) genomes, as well as the genome guided assembly using Farga genome as a reference (GG.) (B) ExN50 of contigs, i.e. N50 as a function of percentage of expression of the top x-expressed genes of the mappings against Arbequina (green), Farga (yellow) and our genome guided assembly (black), expressed as mean values (solid dots) and standard deviation (error bars.)

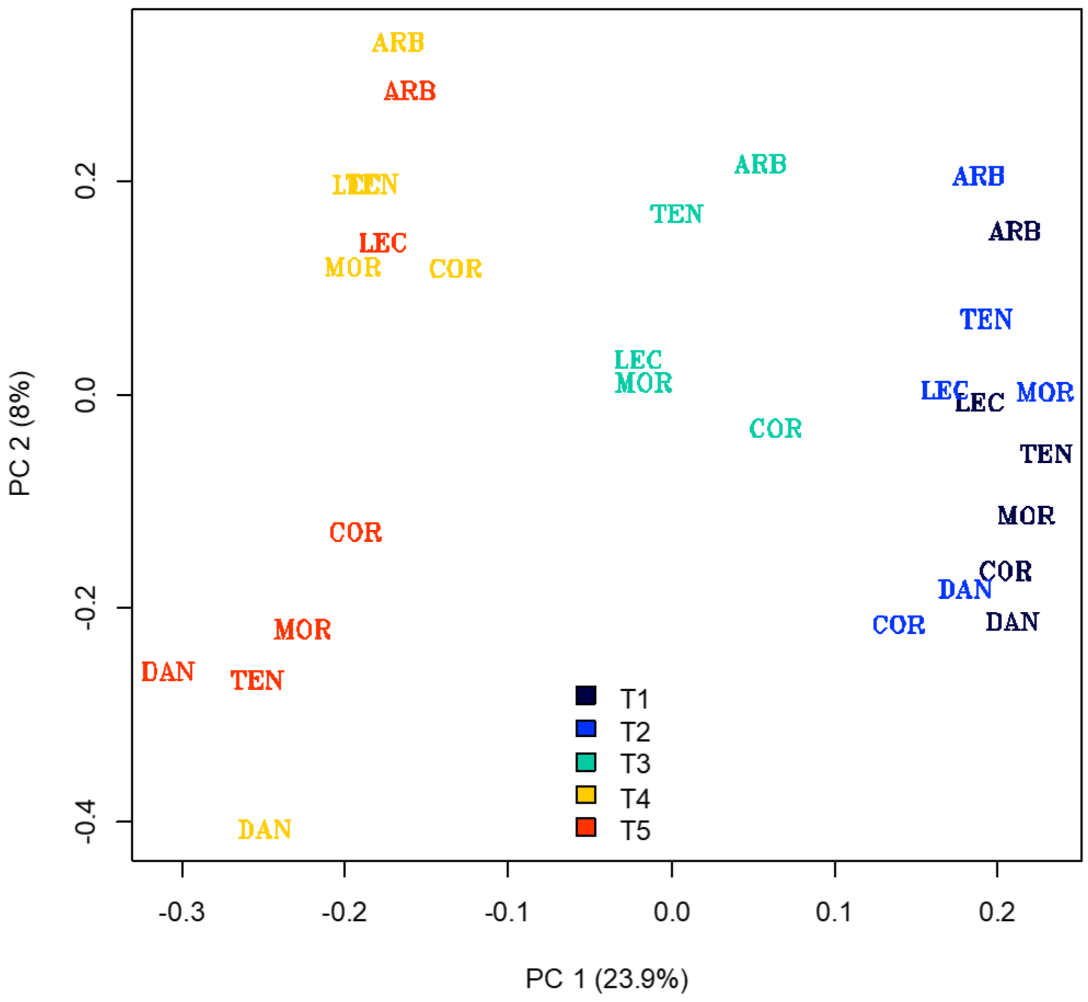

Figure S3. Principal Component Analysis (PCA) of expression data. A loadings plot of the main two components of a PCA of the expression of all genes detected in samples through maturation. Labels correspond to cultivars *Dolce d'Andria* (DAN), *Tendellone* (TEN)*, Arbequina* (ARB), *Leccino* (LEC), *Coratina* (COR) and *Moraiolo* (MOR). Colors correspond to stages 1 (black), 2 (blue), 3 (cyan), 4 (yellow) and 5 (red).

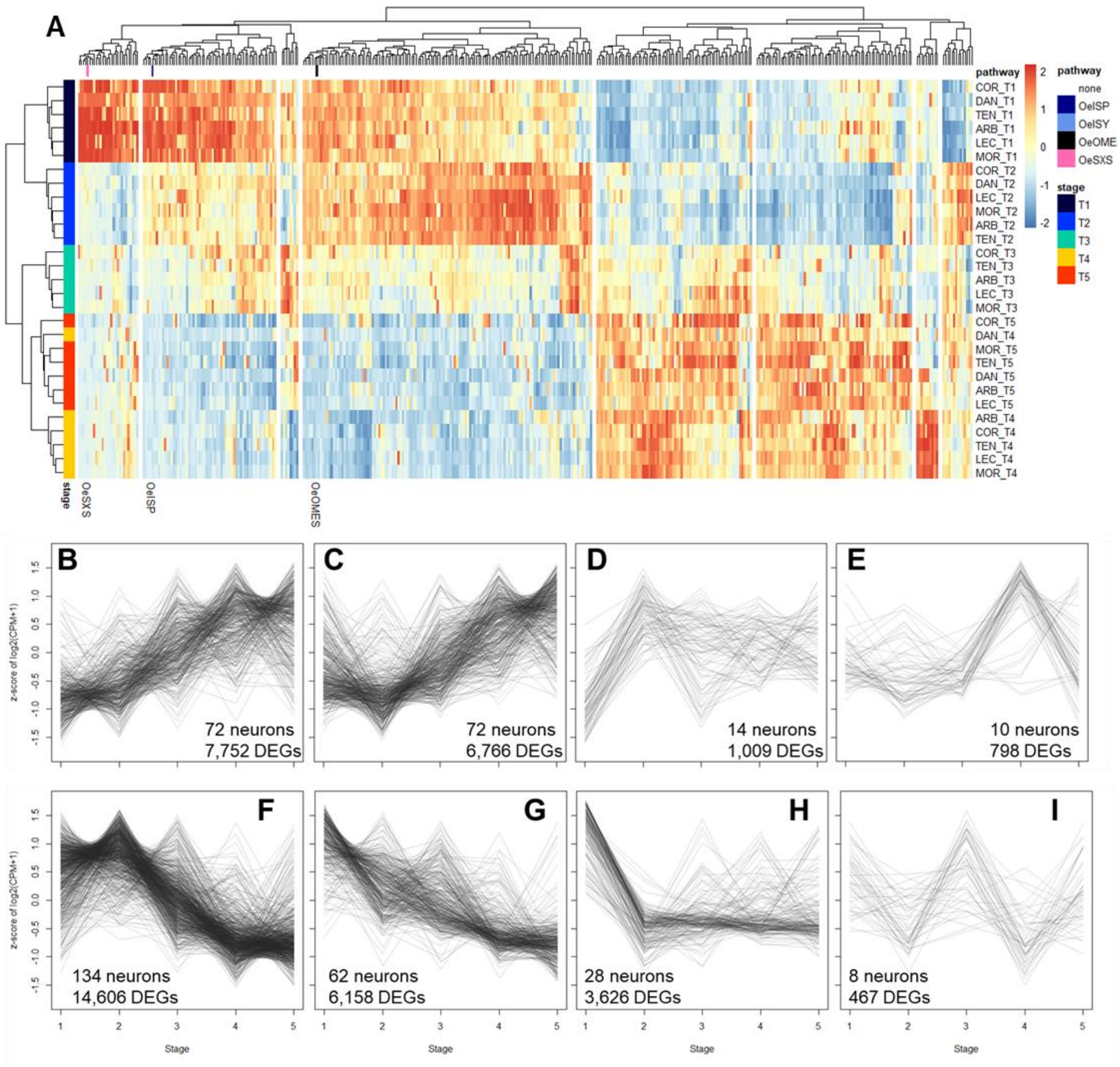

Figure S4. Heatmap of differentially expressed genes during Olive fruit maturation and ripening.
(A) Heatmap of the codebook vectors of the 400 self-organizing map nodes, showing the type expression pattern of the 41,182 differentially expressed genes in olive fruit through maturation. Row band colors correspond to stages 1 (black), 2 (blue), 3 (cyan), 4 (yellow) and 5 (red). **(B-I)** Line plots of the codebook vectors of the 400 self-organizing map nodes, grouped by cluster.

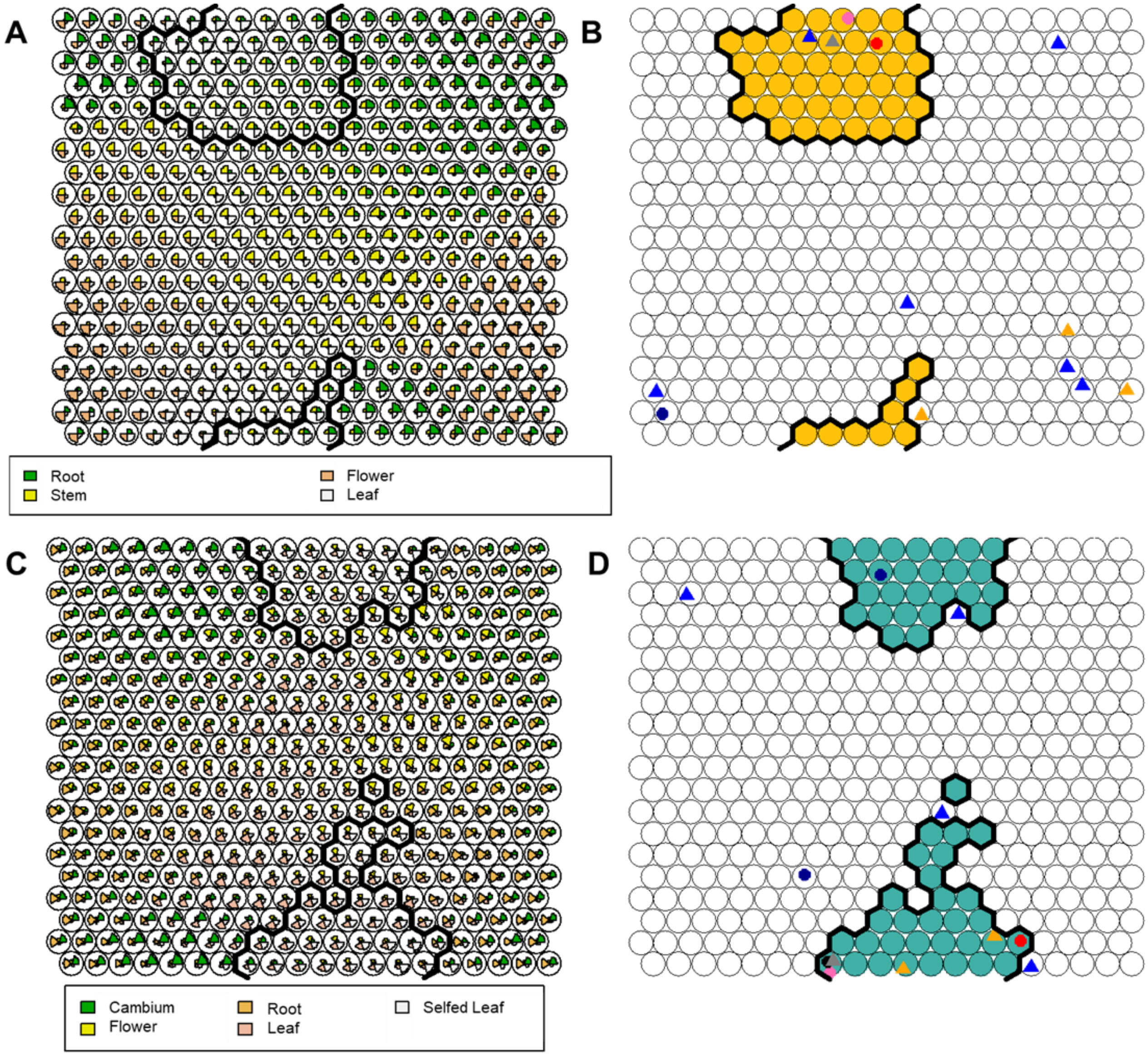

Figure S5. Self-Organizing Maps analysis of *Jasminium sambac* and *Fraxinus excelsior*. Codes plot **(A)** and mapping plots (B) of the self-organizing map (SOM) summarizing expression data of *J. sambac* and codes plot (C) and mapping plots (D) of the SOM for *F. excelsior* expression data. The best BLAST results of known biosynthetic enzymes are shown in the codes plot as figures: ISY, iridoid synthase (black circle); ISP, iridoid synthase paralogue (gray triangle); IO, iridoid oxidase (pink circle); DLGT, 7-deoxyloganetic acid glucosyltransferase (blue triangle); 7eLAMT, 7-*epi*-loganic acid methyl-transferase (dark blue circle); OMES, oleoside methyl ester synthase (red circle); OMEGT, oleoside-11-methyl ester glucosyl transferase (orange triangle). The selected cluster where most biosynthetic genes are located is highlighted in yellow for *J. sambac* and in green for *F. excelsior*.

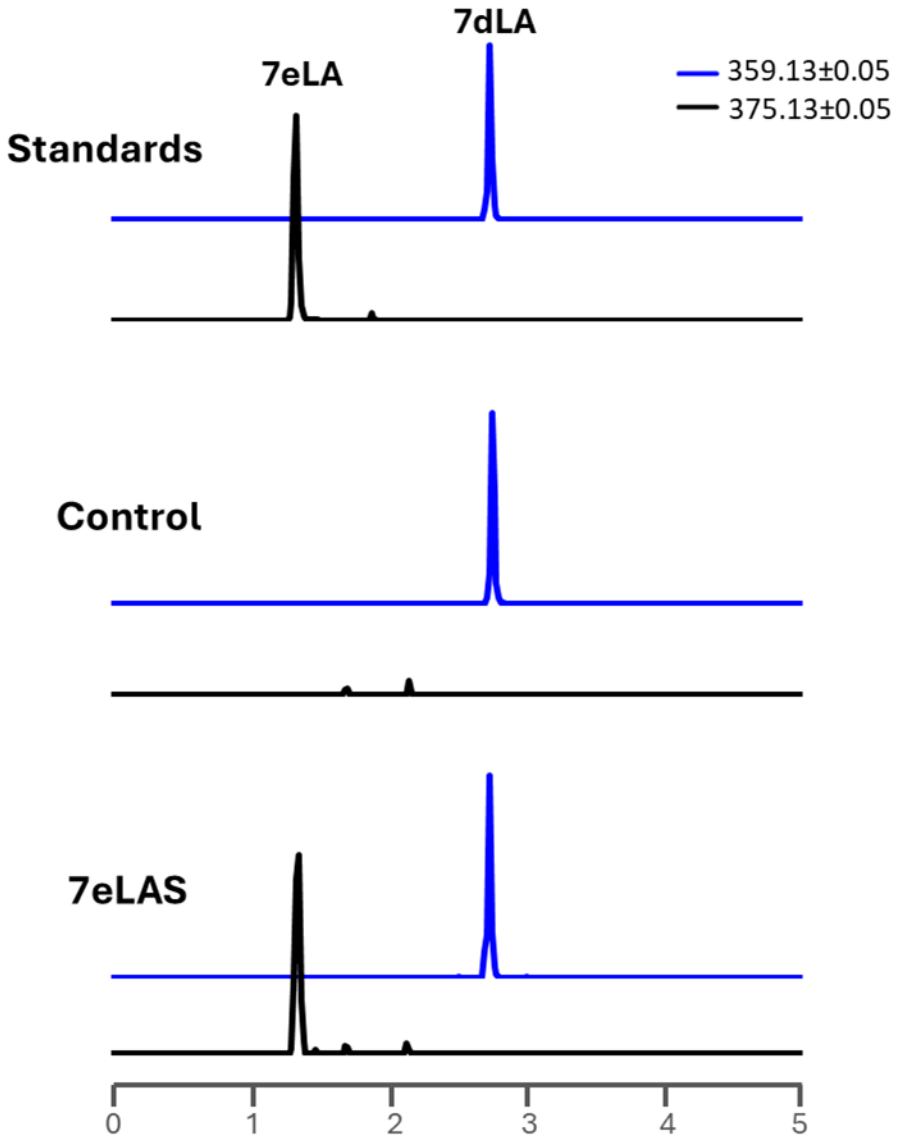

Figure S6. In vitro enzyme assay for Oe7eLAS. Extracted ion chromatogram (XIC) of the most abundant adducts of 7-*epi*-loganic acid (7eLA, $[M\text{-}H]^-$) in black (375.1297±0.05) and 7-deoxy-loganic acid (7dLA, $[M\text{-}H]^-$) in blue (359.1348 ± 0.05). From top to bottom: mix of standards (Standards), and incubations of 7-deoxy-loganic acid with boiled protein (Control) and purified protein (7eLAS), heterologously produced in *E. coli*

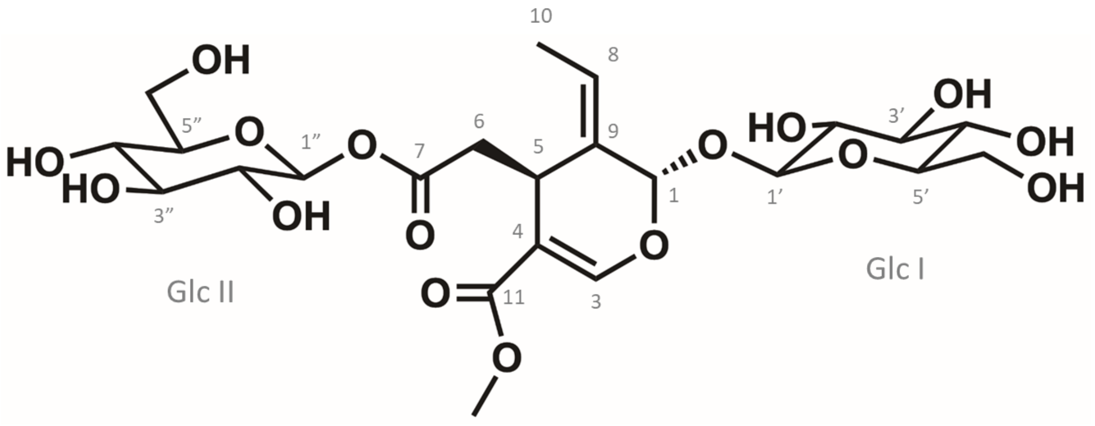

pos.	δ_H	mult.	J_{HH}	δ_C
1	5.94	*dd*	1.3/1.1	95.2
3	7.53	*s*	-	155.2
4	-	-	-	109.0
5	4.01	*dd*	9.2/3.7	31.1
6a	2.79	*dd*	15.4/3.7	40.4
6b	2.59	*dd*	15.4/9.2	40.4
7	-	-	-	171.8
8	6.11	*qd*	7.1/1.1	125.3
9	-	-	-	130.0
10	1.77	*dd*	7.1/1.3	13.7
11	-	-	-	168.6
OMe	3.71	*s*	-	51.8
**Glc I**				
1'	4.81	*d*	7.9	100.7
2'	3.31	*m**	-	74.7
3'	3.41	*dd*	8.7/8.7	78.0
4'	3.31	*m**	-	71.5
5'	3.32	*m**	-	78.3
6'a	3.90	*bd*	12.0	62.7
6'b	3.67	*dd*	12.0/5.9	62.7
**Glc II**				
1'	5.43	*d*	8.2	95.7
2'	3.33	*dd*	8.7/8.2	73.8
3'	3.41	*dd*	9.1/8.7	78.0
4'	3.35	*m**	-	71.0
5'	3.35	*m**	-	78.6
6'a	3.82	*bd*	12.3	62.3
6'b	3.68	*bd*	12.3	62.3
* overlapped signals J unresolved				

Figure S7. Chemical shift data for 7-β-1-D-glucopyranosyl oleoside-11-methyl ester. Chemical shifts table (right) acquired at 700MHz in MeOH-*d*$_3$ is shown along with the elucidated structure.

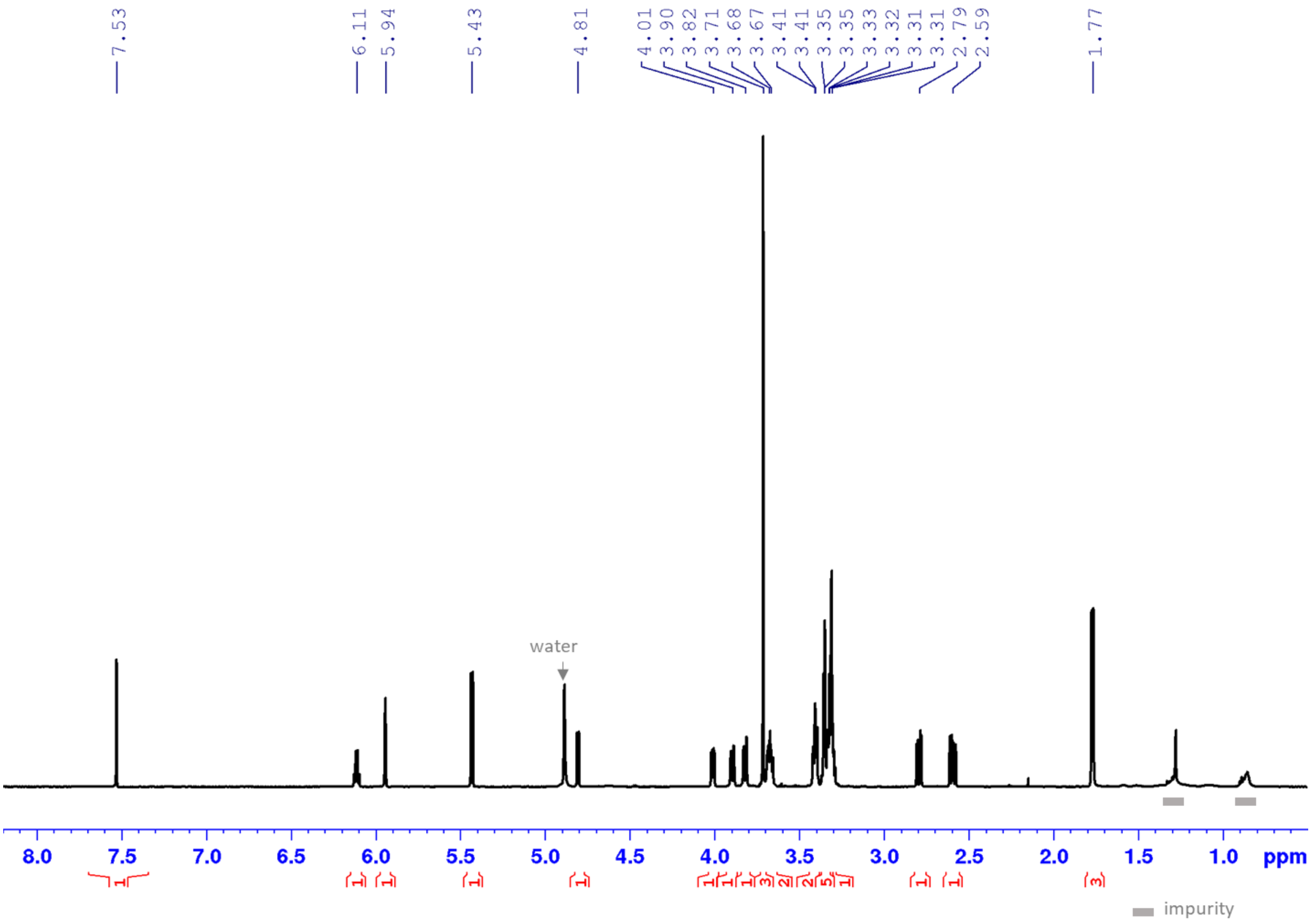

Figure S8. Proton NMR spectra for 7-β-1-D-glucopyranosyl oleoside-11-methyl ester with water suppression. Full range in MeOH-*d*3 is shown.

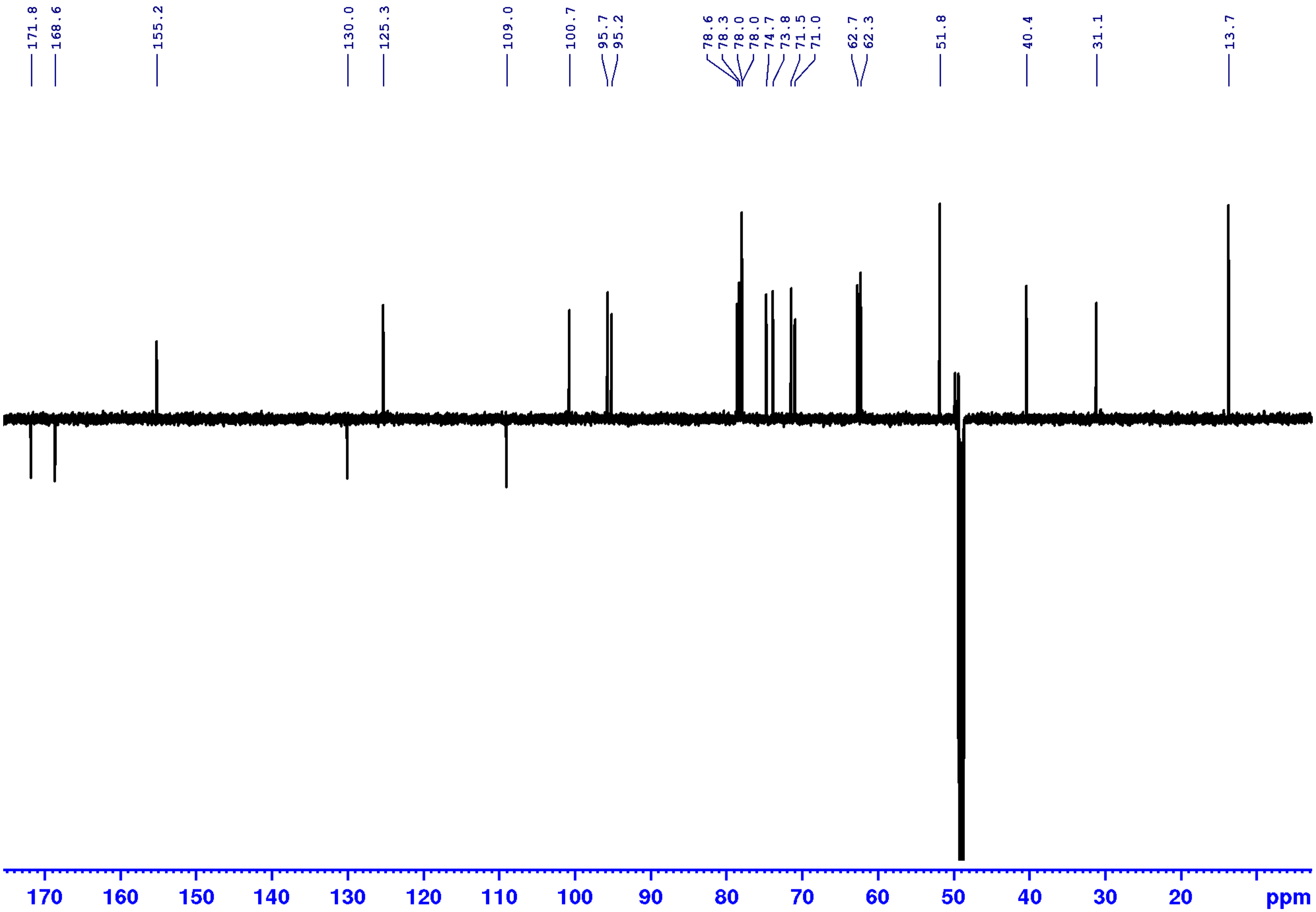

Figure S9. DEPTQ spectra for 7-β-1-D-glucopyranosyl oleoside-11-methyl ester.

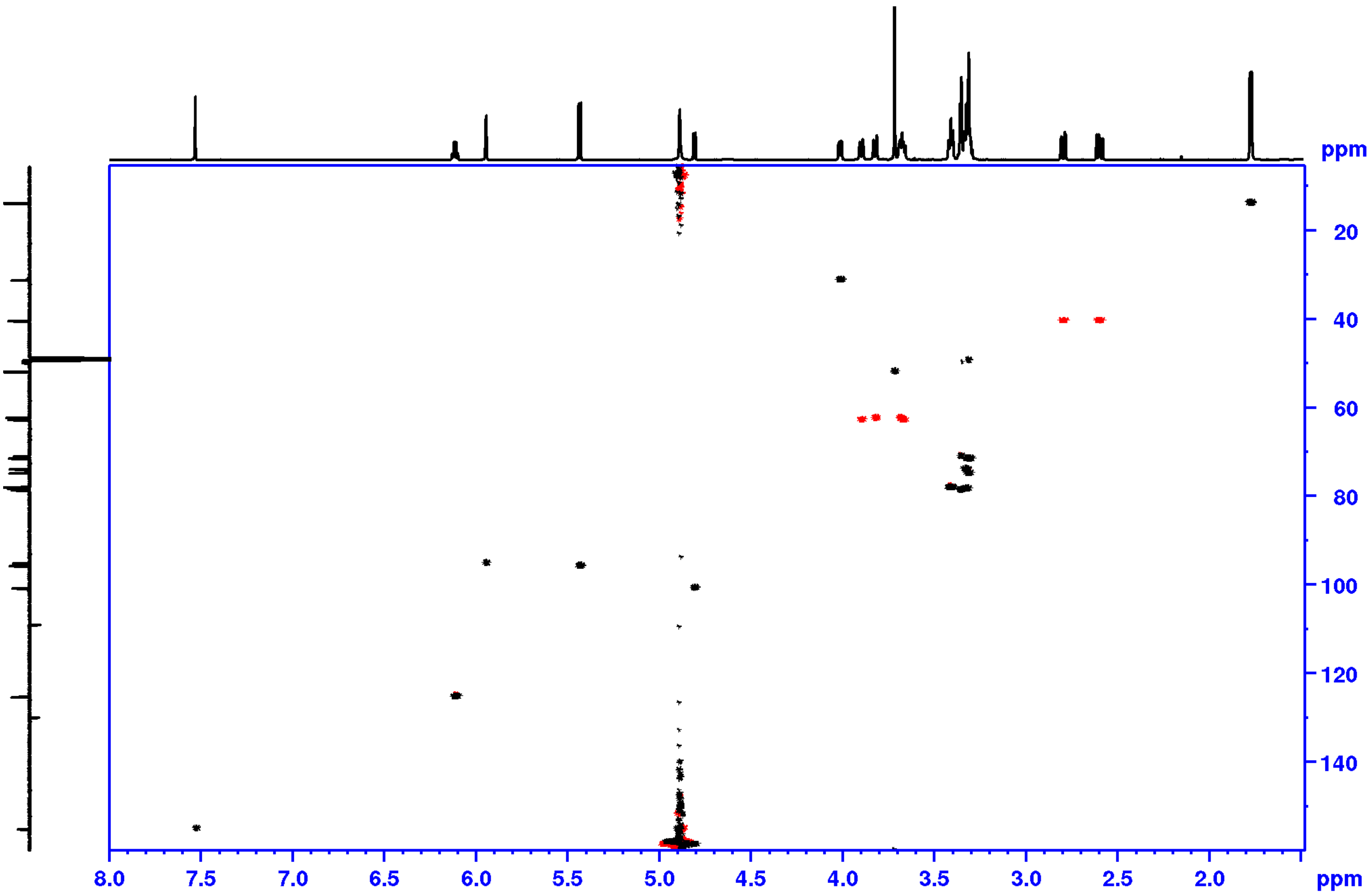

Figure S10. **Full range phase sensitive HSQC spectra for 7-β-1-D-glucopyranosyl oleoside-11-methyl ester. CH/CH_3: black, CH_2: red.**

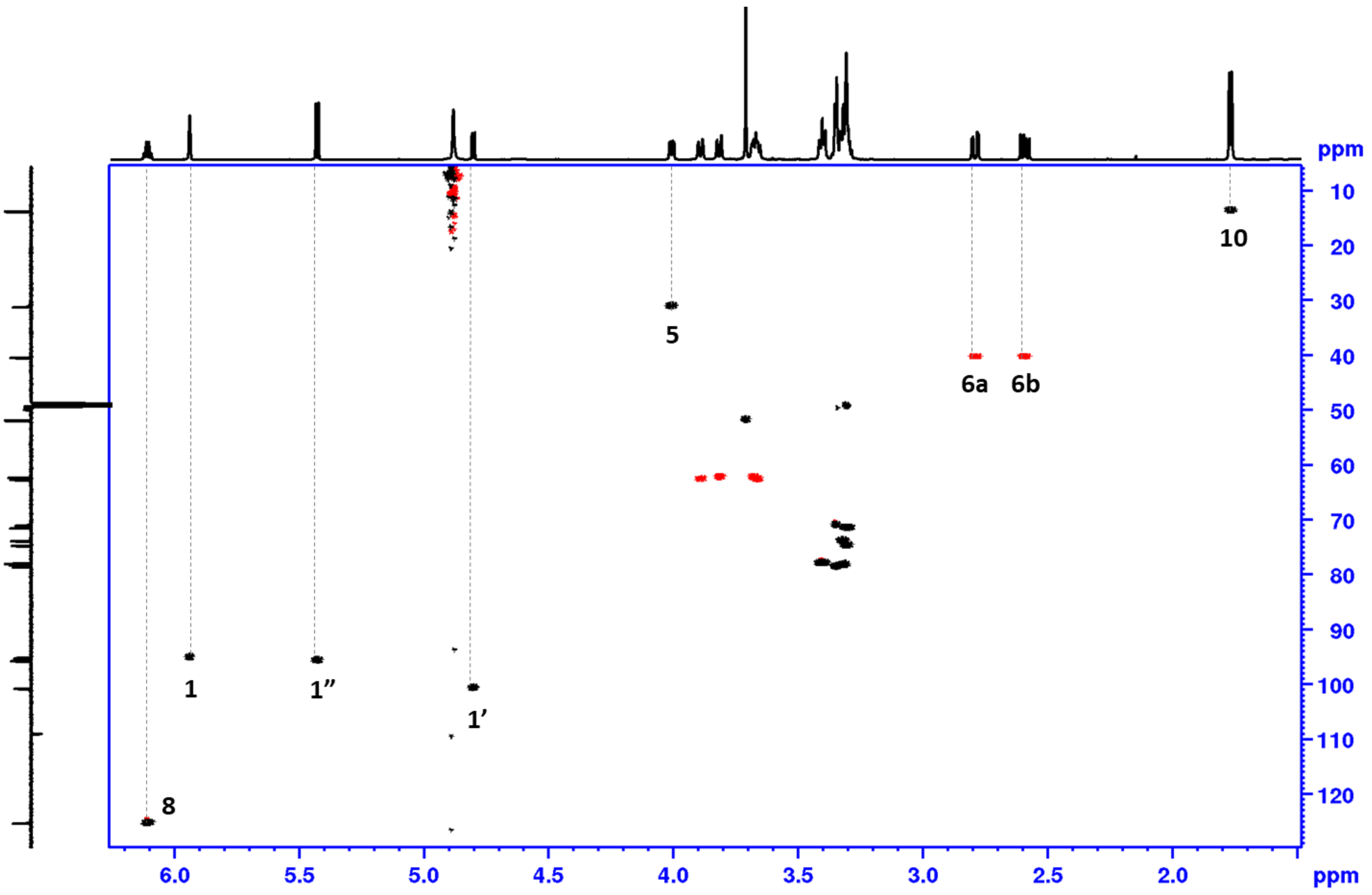

Figure S11. Focused phase sensitive HSQC spectra for 7-β-1-D-glucopyranosyl oleoside-11-methyl ester, from 1.5-6.5 ppm range. CH/CH_3: black, CH_2: red.

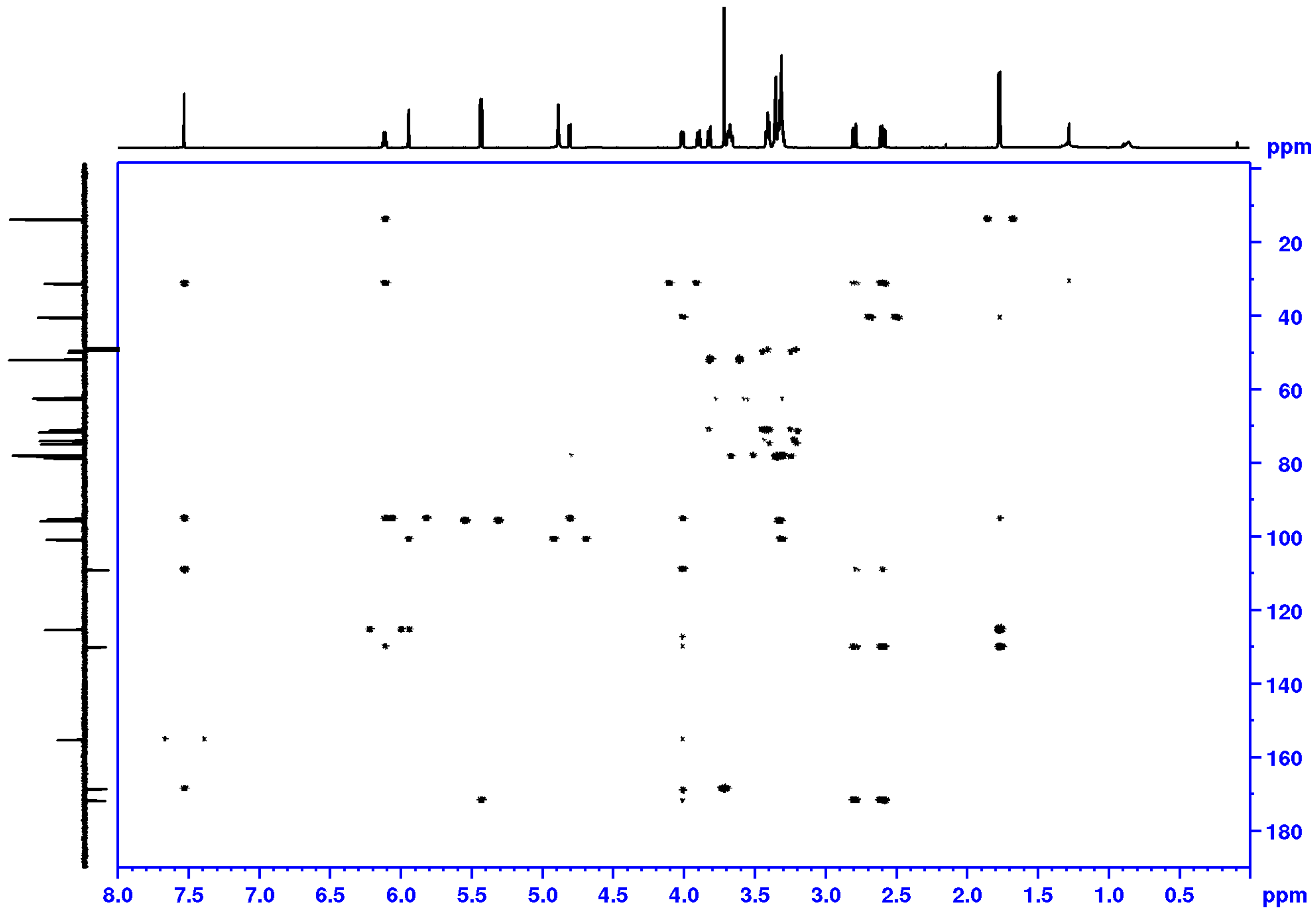

Figure S12. HMBC full range spectra for 7-β-1-D-glucopyranosyl oleoside-11-methyl ester in MeOH-*d*$_3$.

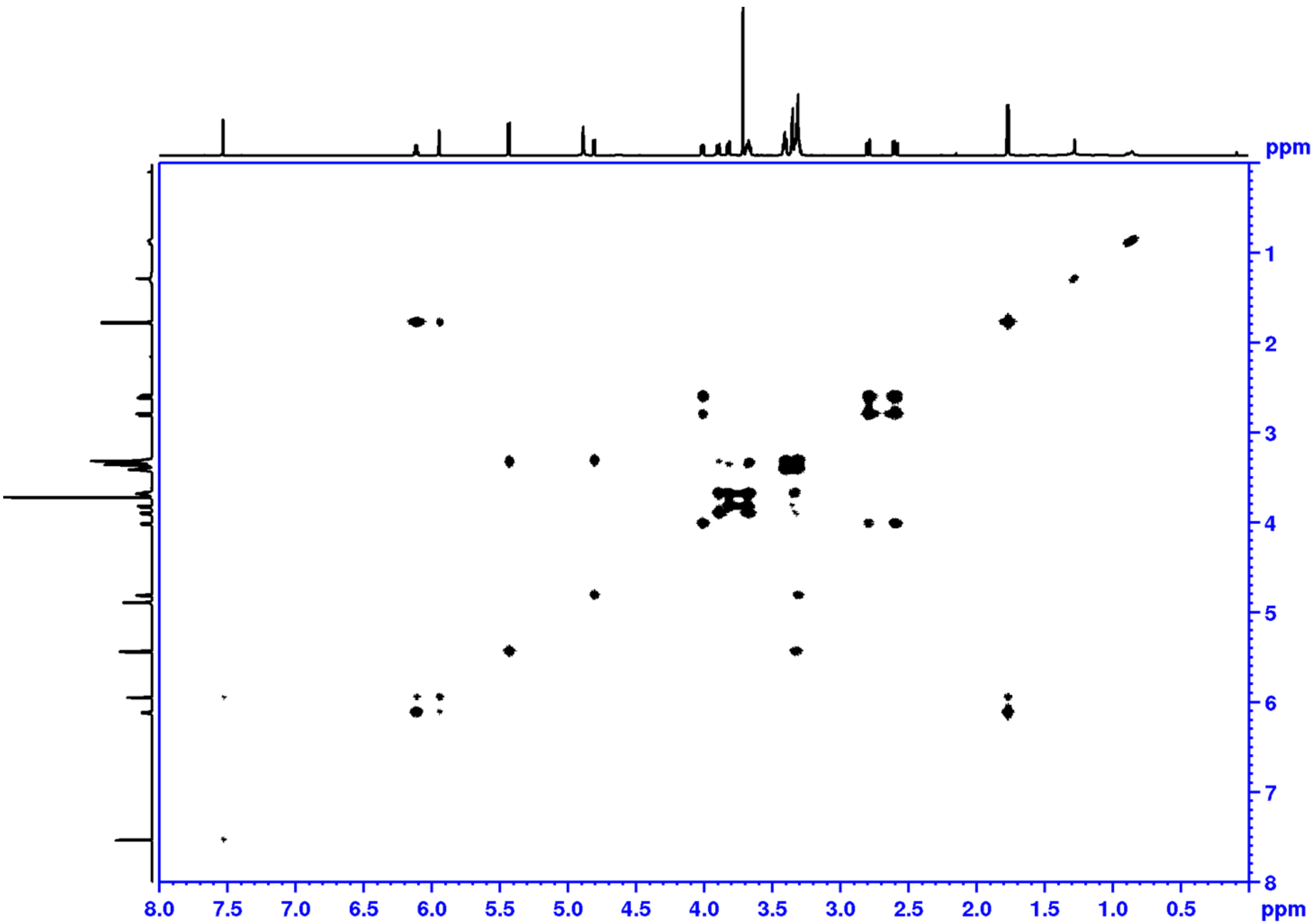

Figure S13. COSY full range spectra with water suppression for 7-β-1-D-glucopyranosyl oleoside-11-methyl ester in MeOH-*d*$_3$

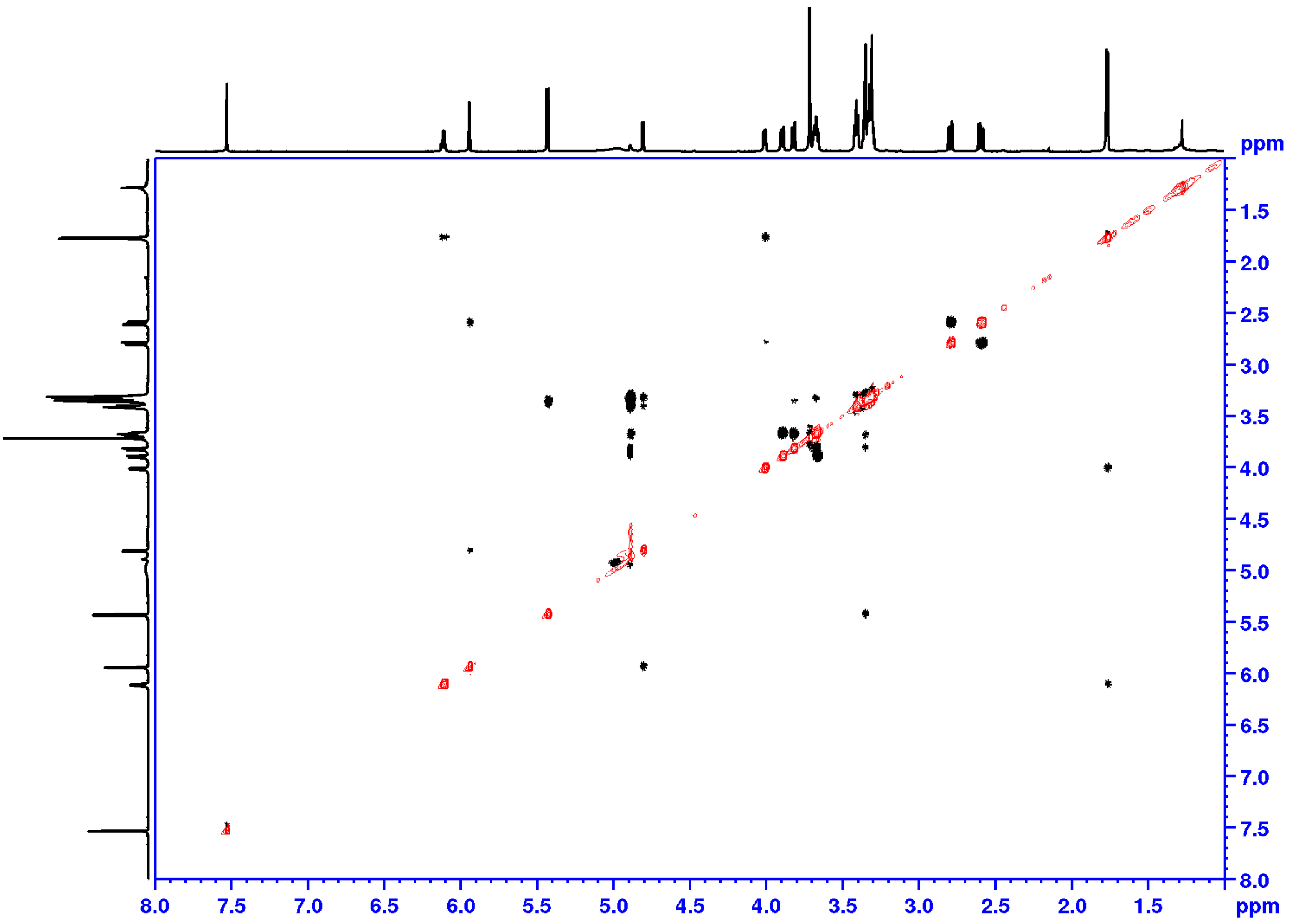

Figure S14. ROESY spectra for 7-β-1-D-glucopyranosyl oleoside-11-methyl ester, with water suppression.

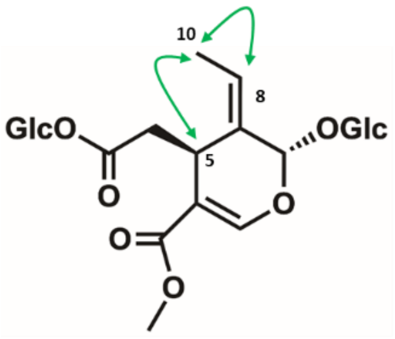

Green arrow shows important ROESY correlations

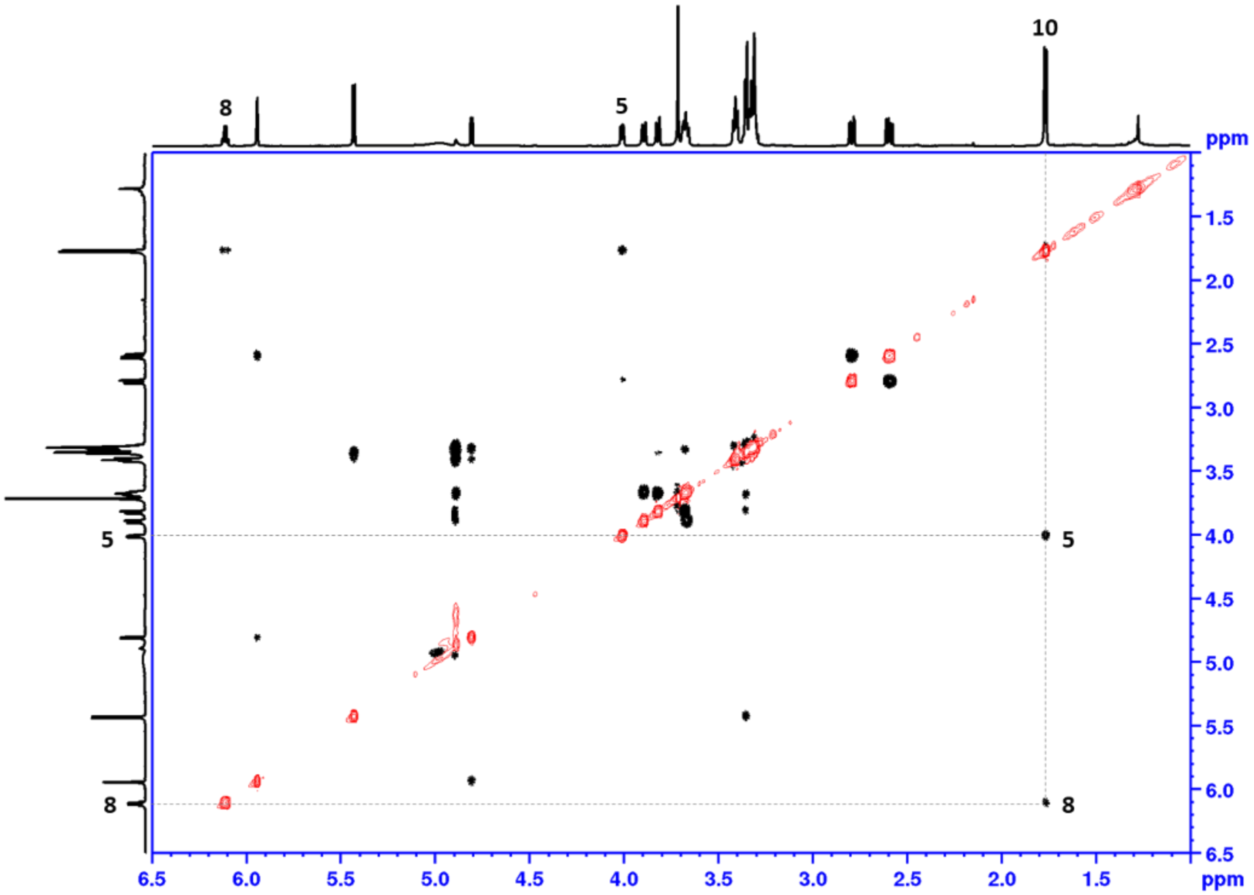

Figure S15. Focused ROESY spectra for 7-β-1-D-glucopyranosyl oleoside-11-methyl ester, with water suppression; range from 1.0-6.5 ppm.

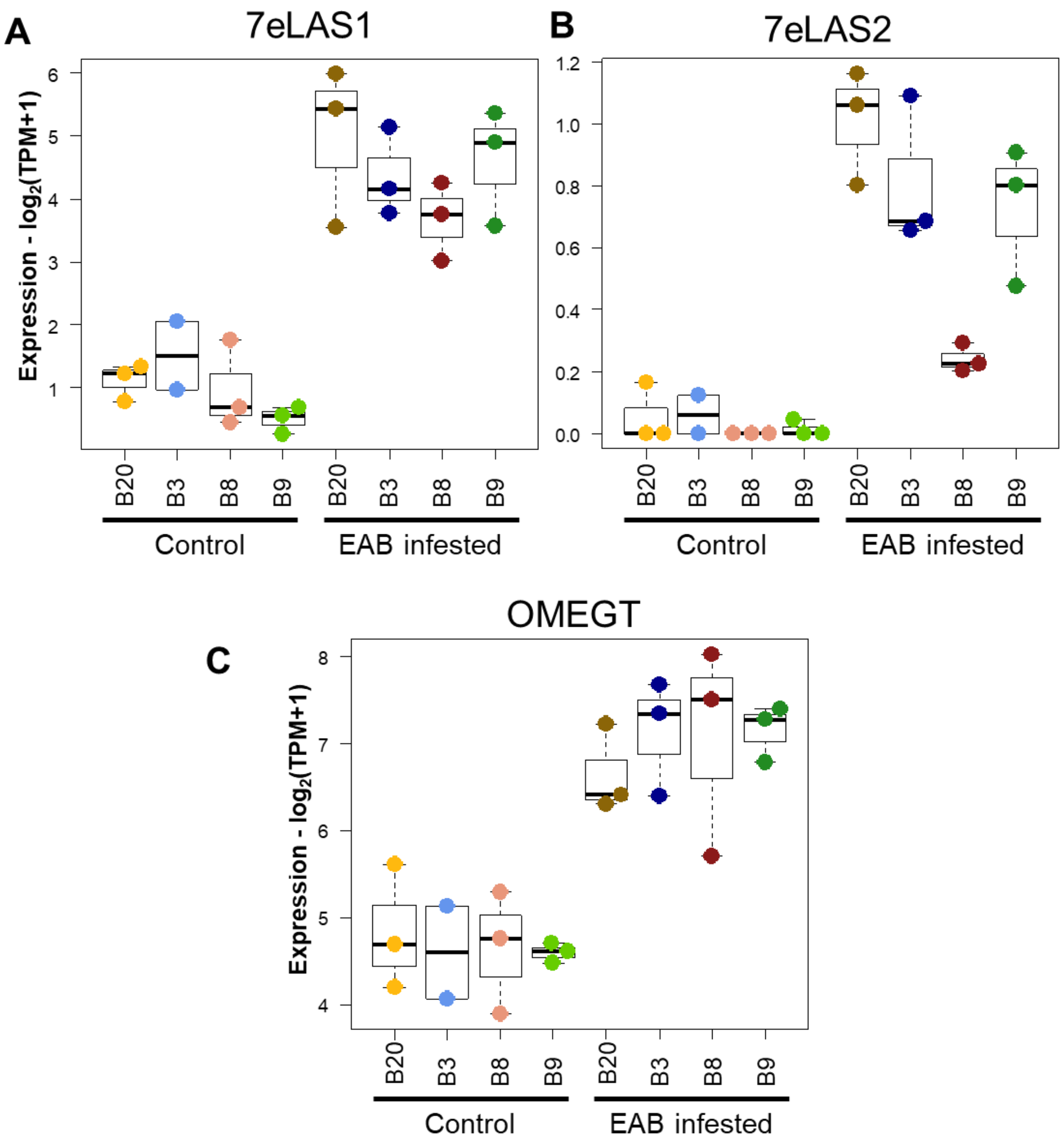

Figure S16. Response of *Fraxinus excelsior* cambium to an Emerald Ash Borer challenge. Expression is shown as a boxplot, with overlaid points for each individual (n=3 per combination) of B3 (cyan/blue), B8 (pink/red), B9 (green/dark green) and B20 (yellow/brown) genotypes infested with Emerald Ash Borer (right) and healthy (left.) RNA-seq data was obtained from project PRJDB15336 (Doonan et al., 2023), mapped against *F. excelsior* genome-guided transcriptome; only statistically significant ($p<0.05$) genes, via two-way ANOVA, are shown.

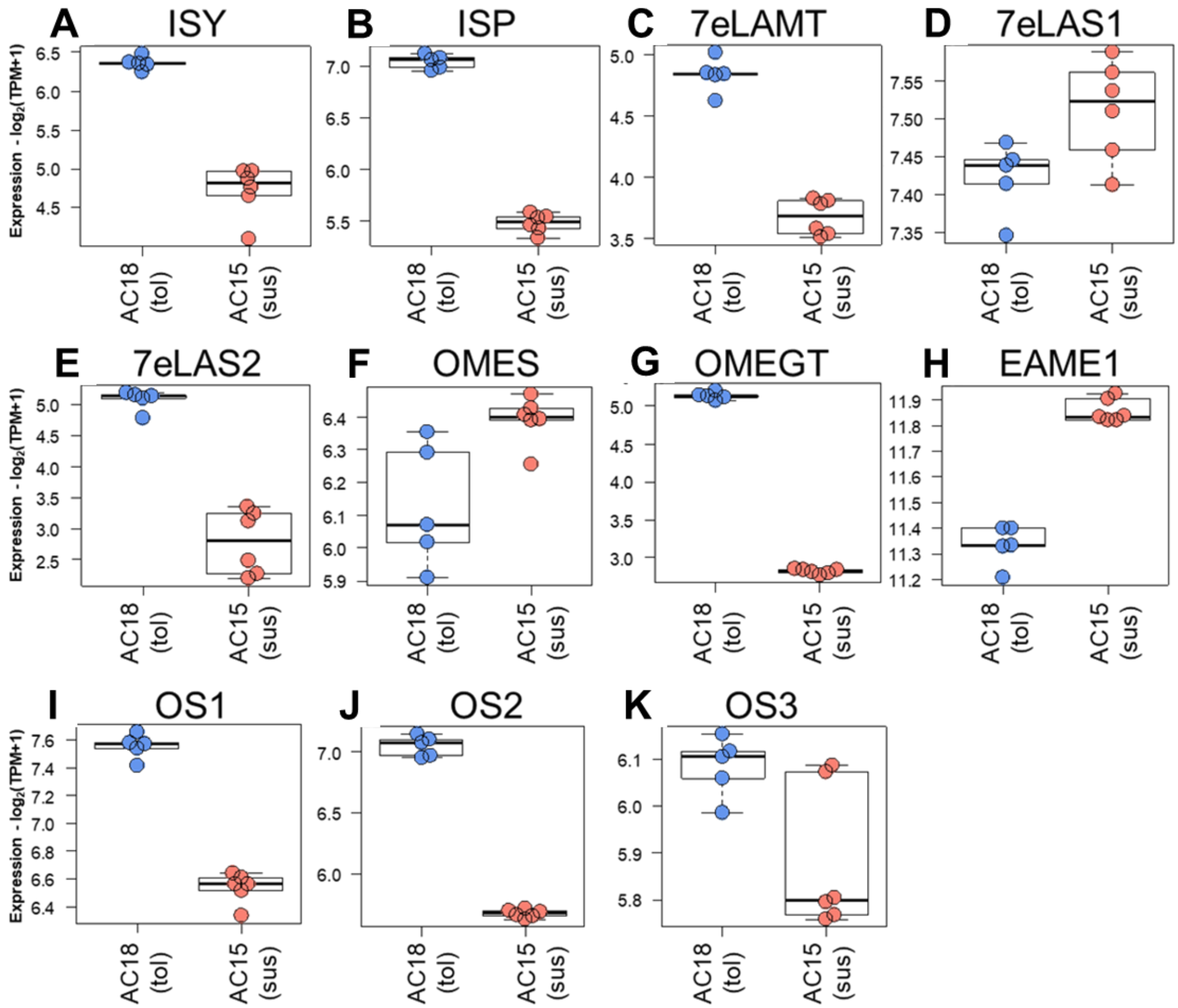

Figure S17. Response of oleaster cultivars AC15 and AC18 to infection by *Verticillium dahliae*. Expression is shown as a boxplot, with overlaid points for each individual of the tolerant cultivar AC18 (n=5) and the susceptible cultivar AC15 (n=6). RNA-seq data was obtained from project PRJNA1030405 (Mascuñano et al., 2025), mapped against the published gene models from the Farga genome (Cruz et al., 2016); only statistically significant ($p<0.05$) genes, via ANOVA, are shown.

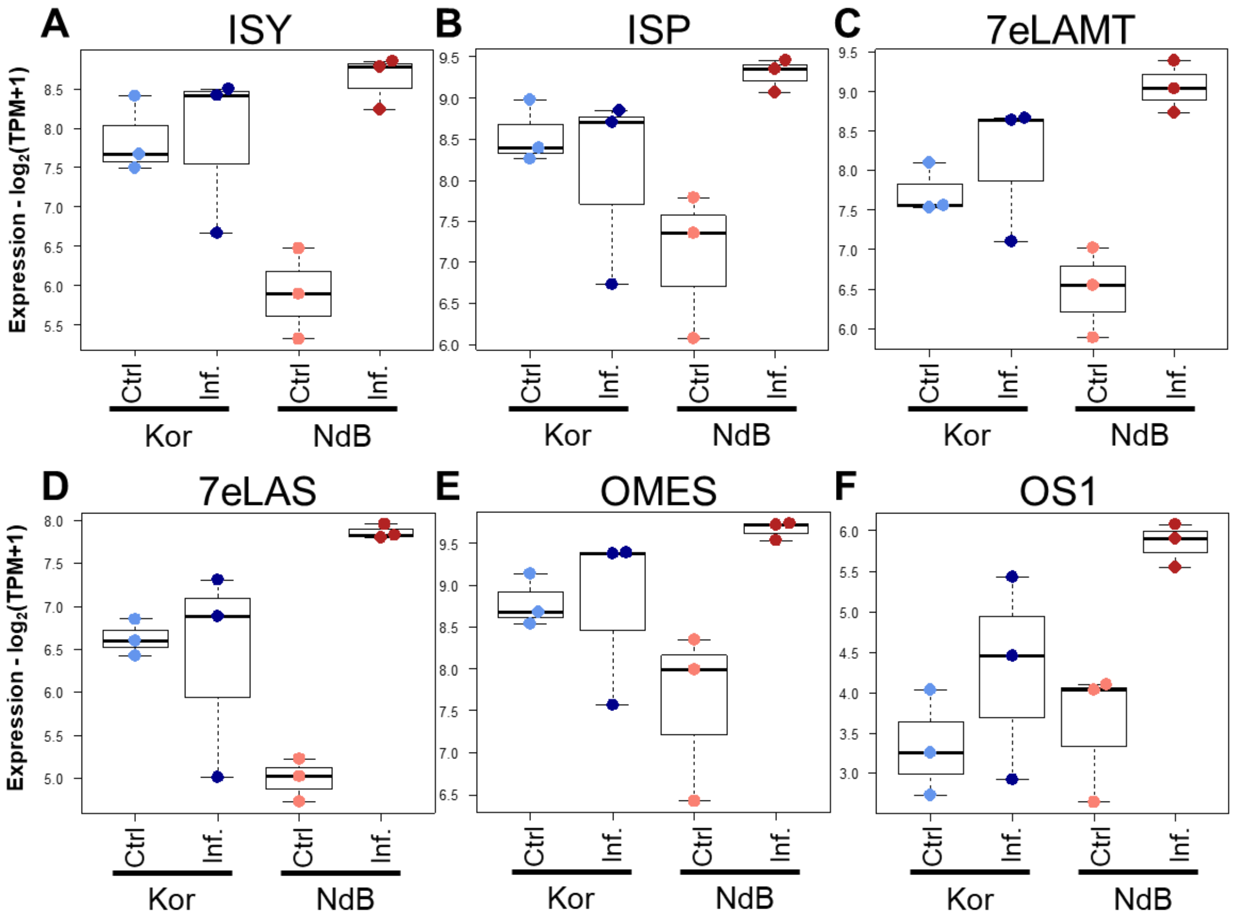

Figure S18. Differential response of olive cultivars Koroneiki and Nocellara del Belice to infection of *Spilocea oleagina*. Expression is shown as a boxplot, with overlaid points for each individual (n=3 per combination) of Koroneiki (Kor; left), a low susceptibility cultivar, and Norcellara del Belice (NdB; left) a highly susceptible cultivar, infected with *Spilocea oleagina* (Inf.) or healthy (Ctrl.) RNA-seq data was obtained from project PRJNA929711 (Marchese *et al*., 2023), mapped against the published gene models from the Farga genome (Cruz et al., 2016); only statistically significant ($p<0.05$) genes, via two way ANOVA, are shown.

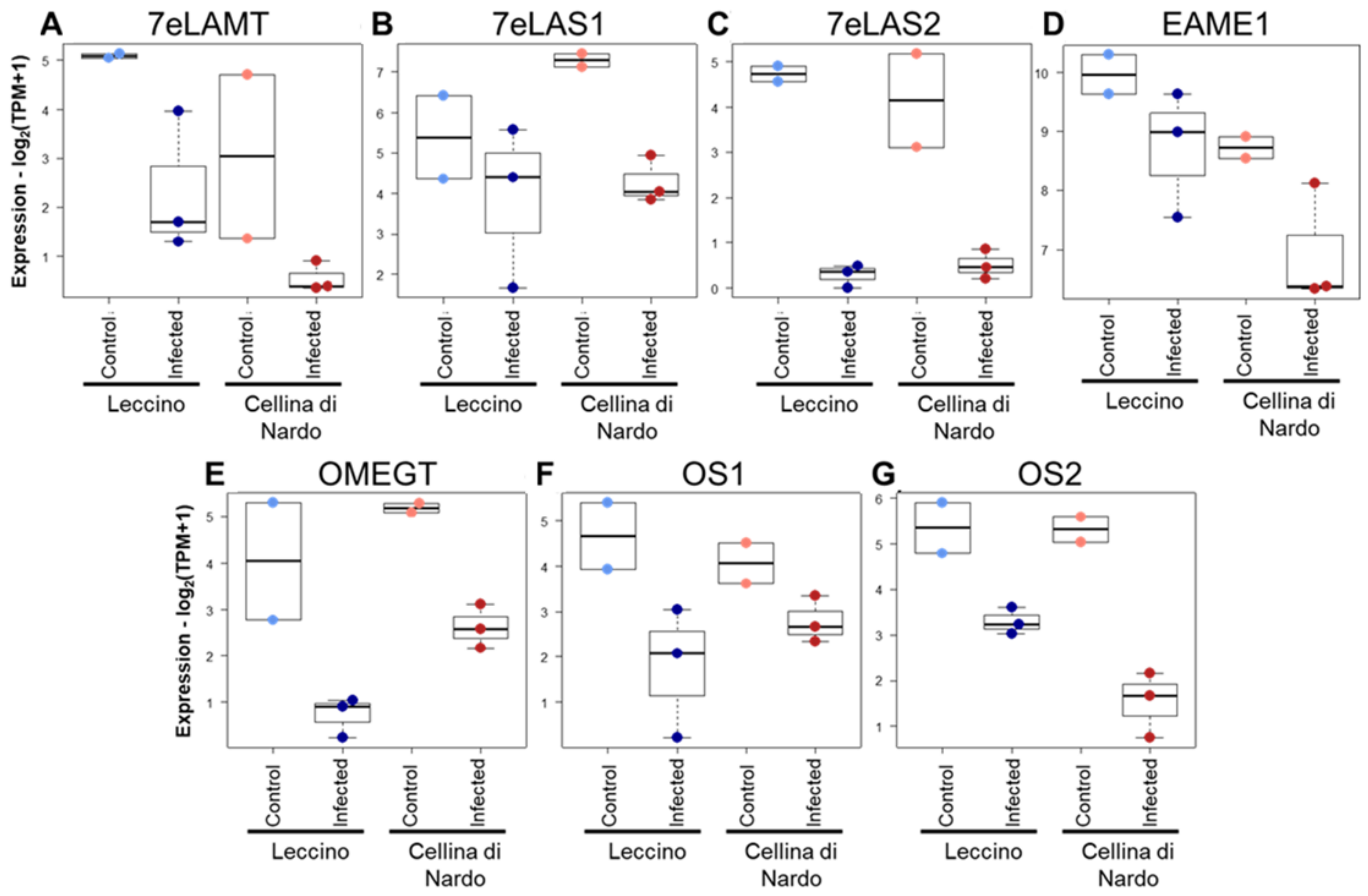

Figure S19. Response of olive cultivars to infection by *Xylella fastidiosa*. Expression is shown as a boxplot, with overlaid points for each individual of Leccino and Cellina di Nardo cultivars, infected with *Xylella fastidiosa* (n=3 per cultivar) or healthy (n=2 per cultivar.) RNA-seq data was obtained from project PRJNA780294 mapped against the published gene models from the Farga genome (Cruz et al., 2016); only statistically significant ($p<0.05$) genes, via two-way ANOVA, are shown.

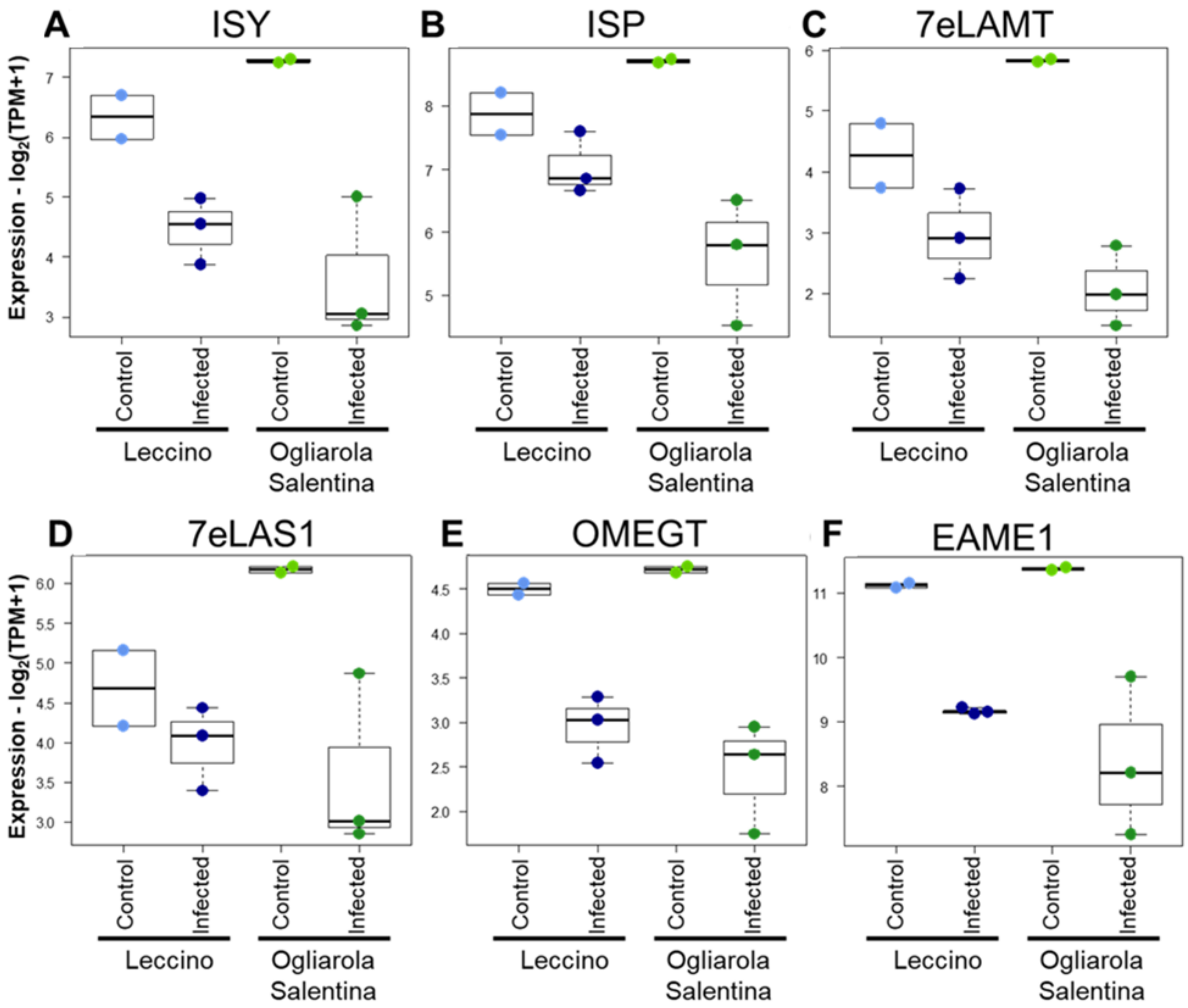

Figure S20. Response of olive cultivars to infection by *Xylella fastidiosa*. Expression is shown as a boxplot, with overlaid points for each individual of Leccino and Ogliarola Salentina cultivars, infected with *Xylella fastidiosa* (n=3 per cultivar) or healthy (n=2 per cultivar.) RNA-seq data was obtained from project PRJNA316374 (Giampetruzzi et al., 2016) mapped against the published gene models from the Farga genome (Cruz et al., 2016); only statistically significant ($p<0.05$) genes, via two-way ANOVA, are shown.
