# Supplementary material for: Comparative transcriptomic and co-expression analyses enable the discovery of key enzymes responsible for oleuropein biosynthesis in olive (Olea europaea)
Source: Plant Commun. 2026 Jan 8;7(4):101713. doi: 10.1016/j.xplc.2026.101713 (PMC13084073; doi:10.1016/j.xplc.2026.101713)
Supplement: Document S2. Article plus supplemental information [file mmc4.pdf]

Plant Communications
Research article

CellPress
Partner Journal

Comparative transcriptomic and co-expression analyses enable the discovery of key enzymes responsible for oleuropein biosynthesis in olive (*Olea europaea*)

Ornella Calderini[1,*], Mohamed O. Kamileen[2], Yoko Nakamura[2], Sarah Heinicke[2], Ryan M. Alam[2], Benke Hong[3], Yindi Jiang[4], Alma Gutiérrez-Vences[5], Fiammetta Alagna[6], Francesco Paolocci[1], Maria Cristina Valeri[1], Edoardo Franco[1], Soraya Mousavi[1], Roberto Mariotti[1], Lorenzo Caputi[2], Sarah E. O'Connor[2,*] and Carlos E. Rodríguez-López[5,7,*]

[1]Institute of Biosciences and Bioresources, CNR, 06128 Perugia, Italy

[2]Department of Natural Product Biosynthesis, Max Planck Institute for Chemical Ecology, 07745 Jena, Germany

[3]Zhejiang Key Laboratory of Precise Synthesis of Functional Molecules, Department of Chemistry, School of Science and Research Center for Industries of the Future, Westlake University, 310030 Hangzhou, China

[4]Key Laboratory of Quantitative Synthetic Biology, Shenzhen Institute of Synthetic Biology, Shenzhen Institutes of Advanced Technology, Chinese Academy of Sciences, Shenzhen 518055, China

[5]Tecnologico de Monterrey, Escuela de Ingenieria y Ciencias, Avenida Eugenio Garza Sada 2501, Monterrey 64700, NL, Mexico

[6]Department of Energy Technologies and Renewable Sources, National Agency for New Technologies, Energy and Sustainable Economic Development (ENEA), Trisaia Research Centre, 75026 Rotondella, MT, Italy

[7]Tecnologico de Monterrey, The Institute for Obesity Research, Integrative Biology Unit, Avenida Eugenio Garza Sada 2501, Monterrey 64700, NL, Mexico

***Correspondence: Ornella Calderini (ornella.calderini@cnr.it), Sarah E. O'Connor (oconnor@ice.mpg.de), Carlos E. Rodríguez-López (c.e.rodriguez@tec.mx)**

https://doi.org/10.1016/j.xplc.2026.101713

ABSTRACT

Olive (*Olea europaea*) is one of the most important crop trees, with olive oil being a key ingredient of the Mediterranean diet. Oleuropein, an oleoside-type secoiridoid, is the major determinant of olive oil flavor and quality. Iridoid biosynthesis has been elucidated in *Catharanthus roseus*, which produces secologanin-type secoiridoids, but iridoid biosynthesis in other species remains unresolved. In this work, we sequenced RNA from the fruit mesocarp of six commercial olive cultivars with various oleuropein contents during maturation and ripening. Using these data, we discovered three polyphenol oxidases with oleuropein synthase (OS) activity, a novel oleoside-11-methyl ester glucosyltransferase (OMEGT) that synthesizes a potential intermediate in the pathway, and a 7-*epi*-loganic acid O-methyltransferase (7eLAMT). Interestingly, the use of transcriptome assemblies for 15 plant species from three iridoid-producing plant orders (Lamiales, Gentianales, and Cornales) for orthogroup inference, and integration of two tissue expression panels from *Jasminum sambac* and *Fraxinus excelsior*, enabled the discovery of two 2-oxoglutarate-dependent dioxygenases (named 7eLAS) that synthesize 7-*epi*-loganic acid; by contrast, *C. roseus* 7-deoxy-loganic acid hydroxylase (7DLH), a known bottleneck in MIA production, is a cytochrome P450. This comparative co-expression method, which combines guilt-by-association and comparative transcriptomics approaches, can successfully leverage large datasets for untargeted discovery of enzymes. Given the increasing availability of expression data from species across the plant kingdom, the methods for gene discovery used in the present work can be readily applied to other untraced pathways.

Key words: enzyme discovery, olive, oleuropein, iridoid biosynthesis, comparative transcriptomics, 2-oxoglutarate-dependent dioxygenase

Calderini O., Kamileen M.O., Nakamura Y., Heinicke S., Alam R.M., Hong B., Jiang Y., Gutiérrez-Vences A., Alagna F., Paolocci F., Valeri M.C., Franco E., Mousavi S., Mariotti R., Caputi L., O'Connor S.E., and Rodríguez-López C.E. (2026). Comparative transcriptomic and co-expression analyses enable the discovery of key enzymes responsible for oleuropein biosynthesis in olive (*Olea europaea*). Plant Comm. **7**, 101713.

 © 2026 The Authors. Published by Elsevier Inc. on behalf of CAS Center for Excellence in Molecular Plant Sciences, Chinese Academy of Sciences, and Chinese Society for Plant Biology.
This is an open access article under the CC BY license (http://creativecommons.org/licenses/by/4.0/).

INTRODUCTION

Olive (*Olea europaea*) is one of the most culturally important crops of Middle Eastern and Mediterranean cultures. Olive oil has been an important part of the Mediterranean diet for millennia, to such an extent that the word for oil in most European languages is derived from the word for olive (Hoad 2003; "aceite," Diccionario de la Lengua Expañola, n.d.). One of the major components of olive oil, and a determinant of its flavor and quality, is oleuropein, a phenolic secoiridoid ester that comprises 6%–14% of the dry weight of the fruit (Amiot et al., 1986; Ryan et al., 1999). Previous research has found that genes involved in oleuropein biosynthesis, namely iridoid synthase (ISY) and oleoside-11-methyl ester (OME) synthase, are more highly expressed in domesticated than in wild olive trees (Rodríguez-López et al., 2021), highlighting the importance of the secoiridoid biosynthetic pathway.

Iridoid glycosides, non-canonical monoterpenes characterized by a cyclopentanopyran fused ring, are one of the most widespread specialized metabolites, present across the largest group of flowering plants, the Asterids (Stull et al., 2018). Although lost on numerous occasions in clades from the order (e.g., Solanales; Stull et al., 2018) to the subclade level (e.g., Nepetoideae; Boachon et al., 2018), iridoids have been used as a chemotaxonomic marker, as their structural diversity shows, for the most part, a clear correlation with phylogeny; this has enabled, for example, the reconstruction of family-wide biosynthetic pathways using phylogenetically aware algorithms (Rodríguez-López et al., 2022). In the Lamiales, an early divergence occurred at the cyclization of 8-oxogeranial, where lineage-specific ISY, in combination with iridoid cyclase (Colinas et al., 2025), differentiates Plantaginaceae and Lamiaceae iridoids from Oleaceae iridoids at the carbon 8 stereoconfiguration (Figure 1). Within the Oleaceae, iridoid chemical diversity is taxonomically partitioned (Jensen et al., 2002), matching the five monophyletic main tribes (Dupin et al., 2024) branching at the oxidation of 7-deoxy-loganic acid: Forsythieae oxidizing carbon 10, Myxophyreae and Fontanesieae oxidizing carbon 7 in the same stereoconfiguration as *Catharanthus roseus* (Gentianales), producing loganic acid, and the sister tribes Jasmineae and Oleae oxidizing carbon 7 with a different stereoconfiguration, producing 7-*epi*-loganic acid (Figure 1).

Iridoids remain critically understudied, and most of what we know of their biosynthesis has been revealed in the "non-model model" *C. roseus* (Apocynaceae; Gentianales). Oleuropein is derived from the oleoside-type secoiridoid OME, which differs from the secologanin-type iridoids present in *C. roseus* and Fontanesieae by a characteristic exocyclic olefin (Figure 1) and has been reported to be present across the Jasmineae and Oleae, with various concentrations and derivatives (Jensen et al., 2002). Despite their importance, the biosynthetic pathway of these compounds has not been fully elucidated, although the early steps are assumed to be the same as those in the well-studied *C. roseus*, despite their chemotaxonomic distribution.

In this work, we sequenced RNA from the fruit mesocarp of six commercial olive cultivars with various levels of oleuropein accumulation during maturation and ripening. Using these data, we performed a comparative co-expression approach, using transcriptome assemblies from 15 plant species in three iridoid-producing plant orders (Lamiales, Gentianales, and Cornales) for orthogroup inference analysis to integrate expression data from tissue panels of two oleoside-type iridoid-producing species (*Jasminum sambac* and *Fraxinus excelsior*). With this approach, we discovered two 2-oxoglutarate-dependent dioxygenases (2-ODDs) that produce 7-*epi*-loganic acid in a stereoselective manner, which we named 7-*epi*-loganic acid synthase (7eLAS) to differentiate them from *C. roseus* 7-deoxy-loganic acid hydroxylase (7DLH), a cytochrome P450 enzyme. Using homology-based approaches, we also identified 7-*epi*-loganic acid O-methyltransferase (7eLAMT) and the novel OME glucosyltransferase (OMEGT), completing the pathway to 7-β-1-D-glucopyranosyl-OME (OME-Glc), reconstructed in *Nicotiana benthamiana*. Finally, we discovered two enzymes from the polyphenol oxidase (PPO) family with oleuropein synthase (OS) activity that produced oleuropein when incubated with ligstroside. This work sets a precedent for leveraging publicly available datasets through a comparative approach, robustly narrowing down gene candidates from tens of thousands to a few hundred, enabling broader hypotheses on the nature of the enzyme candidates.

RESULTS

Transcriptomic profiling of olive fruit mesocarp during maturation and ripening

We collected fruits of six commercial olive cultivars for sequencing. We used the sweet varieties Dolce d'Andria and Tendellone, which are used to produce table olives because of their low oleuropein levels, as well as four varieties containing medium to high levels of phenolic secoiridoids: Arbequina, Leccino, Coratina, and Moraiolo (Alagna et al., 2012; Mousavi et al., 2022). We analyzed the contents of oleuropein and oleuropein precursors at five different time points throughout maturation and ripening. With the exception of OME, all measured metabolites decreased as the fruit matured on the tree, consistent with the available literature (Figure 2) (reviewed by Skodra et al., 2021). Interestingly, varieties with low to moderate iridoid levels had an initial oleuropein content similar to that of the high oleuropein cultivars (Supplemental Figure 1). When the expression of genes likely involved in the degradation pathway was analyzed, a β-glucosidase reported to hydrolyze oleuropein (Koudounas et al., 2015) and two methyl esterases working on the oleuropein and OME aglycones (Volk et al., 2019) were found to be expressed, with only methyl esterase 1 not decreasing during maturation (Supplemental Figure 1). A correlation analysis revealed that only elenolic acid methyl esterase 1 (EAME1) had a relatively high Spearman correlation with iridoid abundances, and it had a notably strong negative correlation with OME, its purported upstream substrate (Supplemental Table 1).

Using the same tissue from which metabolites were extracted, we performed an RNA sequencing (RNA-seq) experiment and assembled a genome-guided transcriptome using the published Farga genome (Cruz et al., 2016) (Supplemental Figure 2A). The resulting transcriptome assembly (Supplemental File 1) had an ExN50 of >2000 kb at 90% of expression and a

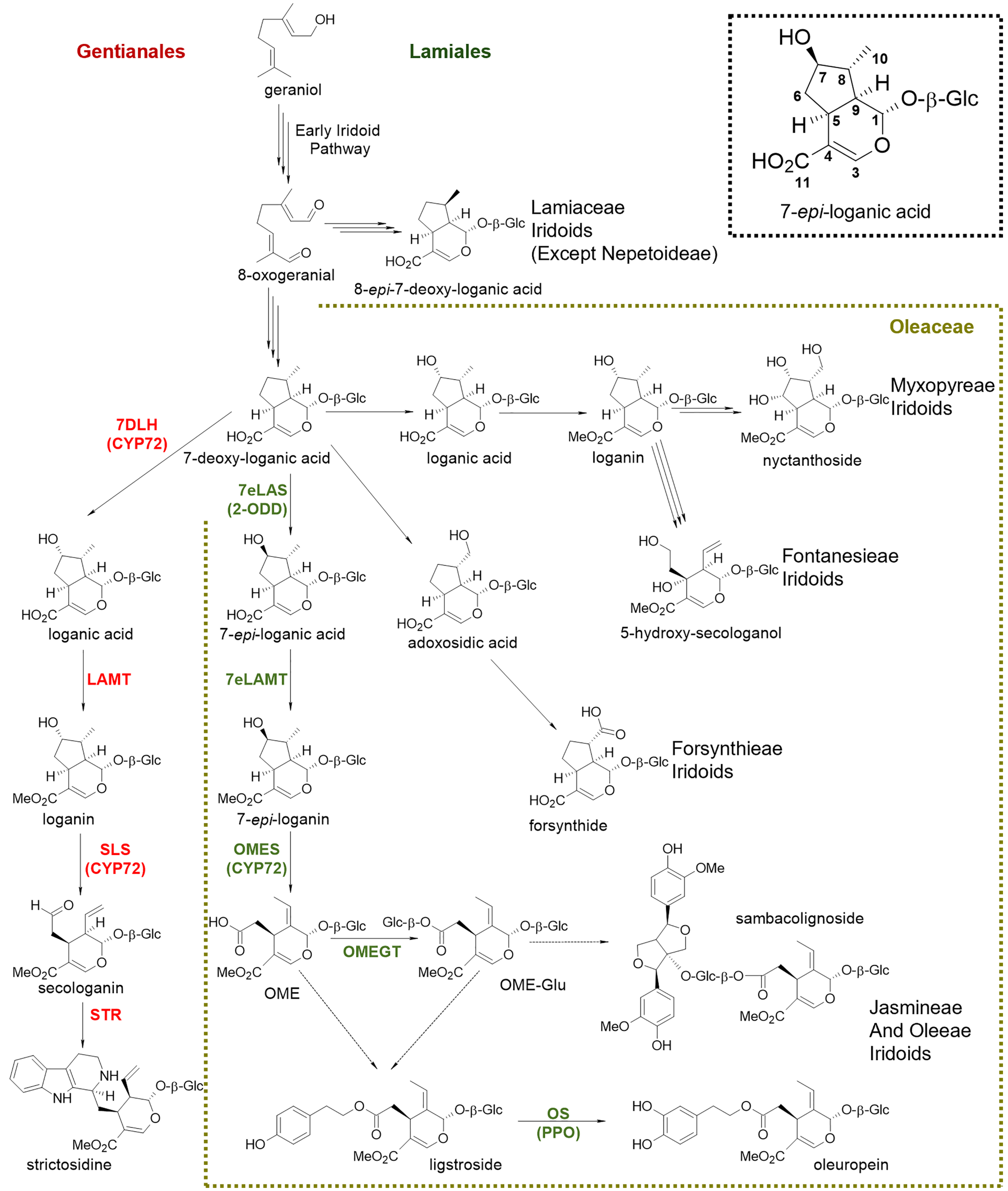

Figure 1. Secoiridoid biosynthetic pathways in commonly studied species.
Enzymes marked in red have been characterized in *Catharanthus roseus*; enzymes in olive green were characterized in this work, with the exception of OMES, which was characterized by our group in previous work (Rodríguez-López et al., 2021). Information on the taxonomic restriction of iridoids within the five tribes of Oleaceae was obtained from the compilation of Jensen et al. (2002). Sambacolignoside has been reported for *Jasminum sambac* and ligstroside and/or oleuropein for several Oleeae, including *Fraxinus excelsior* and *Olea europaea*, and some Jasmineae, but not *J. sambac*. Enzyme

(legend continued on next page)

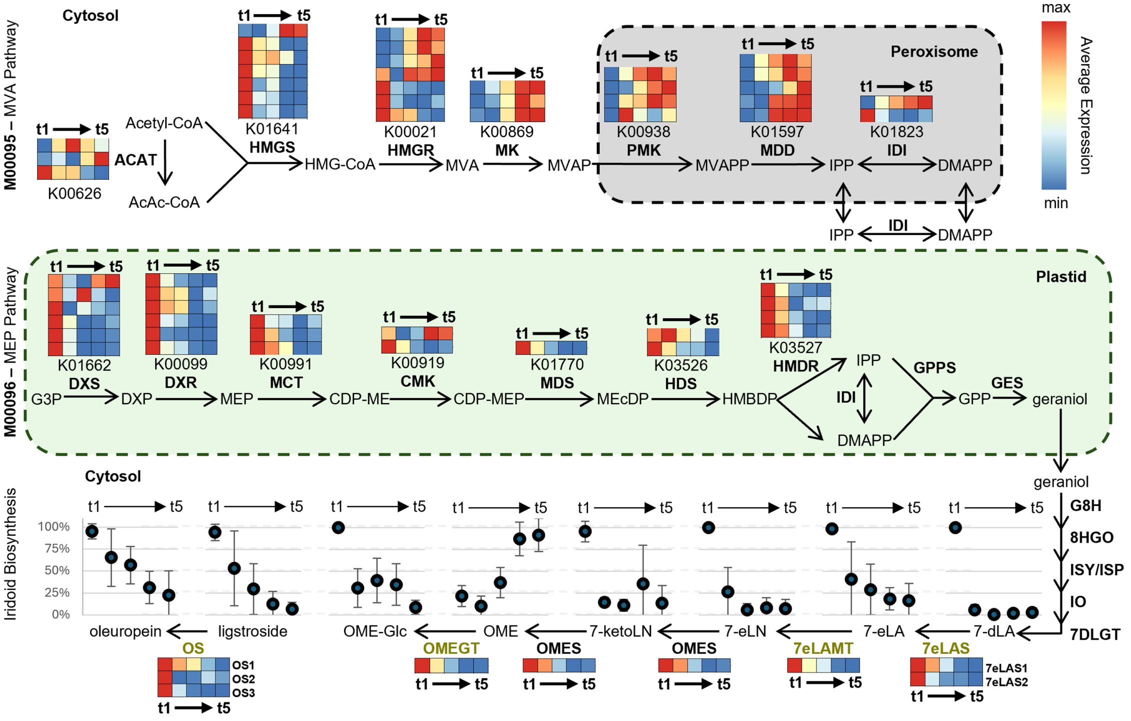

Figure 2. Enriched KEGG modules and iridoid biosynthetic genes.

Schematic of differentially expressed genes from enriched KEGG modules of terpenoid biosynthesis, whose expression increased (KEGG module ID M00095—MVA pathway; top) or decreased (KEGG module ID M00096—MEP pathway; center) during olive maturation. The secoiridoid biosynthetic pathway is shown at the bottom, with enzymes characterized in this work shown in olive green; ligstroside biosynthesis remains unsolved and is therefore represented as a disconnected arrow. Heatmaps show expression patterns of all differentially expressed genes (one per row) annotated with the KEGG orthology number shown below each plot, with colors showing the average (n = 6) of the log-transformed transcripts per million ($\log_2$(TPM + 1)) of all species, with a color scale ranging from the minimum (blue) to the maximum (red) mean expression value per gene (row). Each column of the heatmaps corresponds to a collection time, from left to right, 45, 65, 85, 105, and 125 days after flowering (DAF), with the time series represented by an arrow connecting the first time point (t1: 45 DAF) to the last (t5: 125 DAF). Scatterplots show the mean (dots) and standard deviation (bars) of the intensity, scaled by species, of standard-confirmed iridoids at the five measured stages of maturation (n = 6 for all but the last time point, with n = 5); that is, for each compound, the area under the curve (AUC) of each collection time was divided by the maximum AUC for that compound in a particular species, and that ratio, expressed as a percentage, was averaged for all species at each time point. The x axis represents collection times in the same manner as in the heatmaps, at 20-day regular intervals from 45 to 125 DAF (t1 → t5). MVA pathway: ACAT, acetyl coenzyme A (CoA) acetyltransferase; HMGS, hydroxymethylglutaryl-CoA synthase; HMGR, hydroxymethylglutaryl-CoA reductase; MK, mevalonate kinase; PMK, phosphomevalonate kinase; MDD, mevalonate diphosphate decarboxylase; and IDI, isopentenyl diphosphate delta-isomerase. MEP pathway: DXS, 1-deoxy-D-xylulose-5-phosphate synthase; DXR, 1-deoxy-D-xylulose-5-phosphate reductoisomerase; MCT, 2-C-methyl-D-erythritol 4-phosphate cytidylyltransferase; CMK, 4-(cytidine 5′-diphospho)-2-C-methyl-D-erythritol kinase; MDS, 2-C-methyl-D-erythritol 2,4-cyclodiphosphate synthase; HDS, 4-hydroxy-3-methylbut-2-en-1-yl diphosphate synthase; HMDR, 1-hydroxy-2-methyl-2-(E)-butenyl 4-diphosphate reductase; and GPPS, geranyl pyrophosphate synthase. Iridoid biosynthesis: GES, geraniol synthase; G8H, geraniol 8-hydroxylase; 8HGO, 8-hydroxygeraniol oxidoreductase; ISY, iridoid synthase; ISP, iridoid synthase paralog; IO, iridoid oxidase; 7DLGT, 7-deoxyloganetic acid glucosyltransferase; 7eLAS, 7-*epi*-loganic acid synthase; 7eLAMT, 7-*epi*-loganic acid O-methyltransferase; OMES, oleoside-11-methyl ester synthase; OMEGT, oleoside-11-methyl ester glucosyl transferase; and OS, oleuropein synthase.

mapping average of 90% (Supplemental Figure 2B). Principal-component analysis showed that most changes in expression were due to maturation, with the main principal component (PC1) explaining 24% of the variance and clearly separating the samples by maturation stage (Supplemental Figure 3). Interestingly, although PC2 (8% of the variance) separated samples by cultivar within each ripening state (Supplemental Figure 3), no separation was consistent with ligstroside or oleuropein content in the first 11 components, which together explained 75% of the variance.

A likelihood ratio test, blocked by cultivar, revealed a total of 41 182 differentially expressed genes (DEGs; false discovery rate <0.01) whose expression changed during ripening. Using a self-organizing map (SOM) for dimensionality reduction and performing hierarchical clustering analysis on the resulting

abbreviations: 7DLH, 7-deoxy-loganic acid hydroxylase; 7eLAS, 7-*epi*-loganic acid synthase; SLS, secologanin synthase; STR, strictosidine synthase; LAMT, loganic acid O-methyltransferase; 7eLAMT, 7-*epi*-loganic acid O-methyltransferase; OMES, oleoside methyl ester synthase; OMEGT, oleoside-11-methyl ester glucosyl transferase; and OS, oleuropein synthase. Protein families: CYP72, cytochrome P450 CYP72 family; 2-ODD, 2-oxoglutarate-dependent dioxygenase; and PPO, polyphenol oxidase. Compounds: OME, oleoside-11-methyl ester, and OME-Glc, 7-β-1-D-glucopyranosyl oleoside-11-methyl ester.

codebook vectors, we clustered the DEGs into eight distinct expression patterns (Supplemental Figure 4A); these fell into two basic categories, increasing (Supplemental Figure 4B–4E) and decreasing (Supplemental Figure 4F–4I) with maturation at different rates. An enrichment analysis showed that genes upregulated during olive fruit maturation were enriched in Kyoto Encyclopedia of Genes and Genomes (KEGG) pathway annotations consistent with sugar catabolism and respiration, as well as fatty acid biosynthesis (Supplemental Table 2). On the other hand, downregulated genes were overrepresented in processes related to photosynthesis, the cell wall, and biosynthesis of secondary metabolites, particularly terpenoids (Supplemental Table 2). Interestingly, enrichment analysis of KEGG module annotations suggested that the mevalonate pathway was upregulated and the non-mevalonate pathway was downregulated as maturation progressed (Figure 2; Supplemental Table 3). Iridoids are derived from geraniol produced by the MEP pathway (Contin et al., 1998). Consistently, the accumulation of measured metabolites (with the exception of OME) and the expression pattern of secoiridoid biosynthetic genes decreased during ripening (Figure 2). Thus, to identify missing enzymes in the oleuropein biosynthetic pathway, we focused on the 24 857 genes that were differentially downregulated during ripening.

Comparative co-expression analysis reveals that an oxoglutarate dependent dioxygenase (ODD) hydroxylates 7-deoxy-loganic acid to produce 7-*epi*-loganic acid

The early steps of iridoid biosynthesis are the same in both olive and *C. roseus*. The first step in which the chemistry diverges occurs after the formation of 7-deoxy-loganic acid. In *C. roseus*, 7-deoxy-loganic acid is hydroxylated by 7DLH (Cr7DLH), a cytochrome p450 from the CYP72 family, to form loganic acid (Salim et al., 2013), whereas in olive, 7-deoxy-loganic acid is hydroxylated to form 7-*epi*-loganic acid. We initially assumed that the 7eLAS from olive would be a homolog of Cr7DLH; however, no olive protein with sequence similarity to Cr7DLH showed any hydroxylation activity on 7-deoxy-loganic acid. We therefore widened our search; however, a guilt-by-association approach yielded too many gene candidates to test, owing to the confounding factor of fruit ripening.

Because the early iridoid pathway is shared across the Asterids, we performed a comparative co-expression analysis to reduce the number of candidate genes. Genome-guided transcriptome assemblies were generated for five Lamiales species and integrated with published data from other iridoid producers within the Asterids, for a total of 15 plant species: nine Lamiales, five Gentianales, and one Cornales (Supplemental Table 4). We focused on members of the Oleaceae within our selection that had reliable reports of secoiridoid accumulation and available RNA-seq data for aerial, underground, and reproductive tissues. We therefore selected project PRJNA723725, which contained RNA-seq data on leaf, flower, stem, and root tissues of *J. sambac*, and project PRJEB4958, which contained RNA-seq data on leaf, flower, cambium, and root tissues of *F. excelsior. J. sambac* and *F. excelsior* have been reported to accumulate secoiridoids in leaf tissue (Ross et al., 1982; Damtoft et al., 1992; Jensen et al., 2002), and although no information is available for other tissues, we can reasonably assume that at least one of the selected tissues will have little to no secoiridoid biosynthesis. RNA-seq data were mapped against their respective genome-guided assemblies, and expression patterns were estimated using a Z score calculated on log-transformed transcripts per million values. The codebook vectors from SOMs were used to perform hierarchical clustering analysis, which enabled us to visually distinguish eight expression patterns (Figure 3). For each species, we selected the best BLAST results for biosynthetic genes in the early iridoid pathway against each genome-guided assembly, and we selected the cluster that contained the highest number of early biosynthetic gene candidates as the cluster likely to contain the missing step (Supplemental Figure 5).

As seen in Supplemental Figure 4A and 4B, in *J. sambac*, candidates for ISY paralog (ISP), iridoid oxidase, 7-deoxyloganetic acid glucosyltransferase, and oleoside methyl ester synthase (OMES) grouped together in a cluster that was expressed in every tissue except flowers (Figure 3A); we therefore selected 2561 transcripts (from 1818 orthogroups) with that expression pattern for further analysis. Interestingly, in *F. excelsior*, candidate biosynthetic genes (ISY, ISP, iridoid oxidase, 7eLAMT, and OMES) also clustered together (Supplemental Figure 5C and 5D). However, they showed expression in leaves and flowers and little to no expression in roots and cambium tissue (Figure 3B), yielding almost twice as many candidates (4238 genes; 2790 orthogroups). These candidate lists were integrated with the DEGs from olive ripening, keeping only those genes that were differentially expressed in olive during ripening and also had an orthogroup member in the candidate list for *J. sambac* and *F. excelsior*. There were 321 orthogroups that met these criteria (Figure 3C), which led to a reduction of olive gene candidates from the initial 24 857 DEGs to 789, only 332 of which had a Pfam annotation.

Among these candidates, annotations related to oxidases included eight multicopper oxidases, which were unlikely to catalyze the expected reaction and were not explored further; six cytochrome p450 enzymes; five ODDs; one Rieske oxygenase; and one peroxidase. When tested by transient expression in *N. benthamiana* leaves, with 7-deoxy-loganic acid infiltrated as a substrate, contigs TRINITY_GG_13709_c0_g1_i1 and TRINITY_GG_36519_c0_g1_i2, annotated as ODDs, were shown to consume 7-deoxy-loganic acid and produce 7-*epi*-loganic acid in a stereoselective manner, with no other epimer detected (Figure 3D). This activity was confirmed by assays of purified enzymes heterologously expressed in *Escherichia coli*, which produced a single peak in the product chromatogram, coinciding with 7-*epi*-loganic acid (Supplemental Figure 6). To avoid confusion with the 7DLH reported in *C. roseus*, which is a cytochrome P450, these ODDs were named *O. europaea* 7eLAS1 (Oe7eLAS 1) and Oe7eLAS2, respectively. Interestingly, despite showing low co-expression with each other (r = 0.46), their coding sequences have 91% identity, and their peptide sequences share a 95% similarity. Notably, *C. roseus* has eight members in this orthogroup, including deacetoxyvindoline 4-hydroxylase, an enzyme from the late MIA pathway that is expressed in leaf idioblasts (Li et al., 2023).

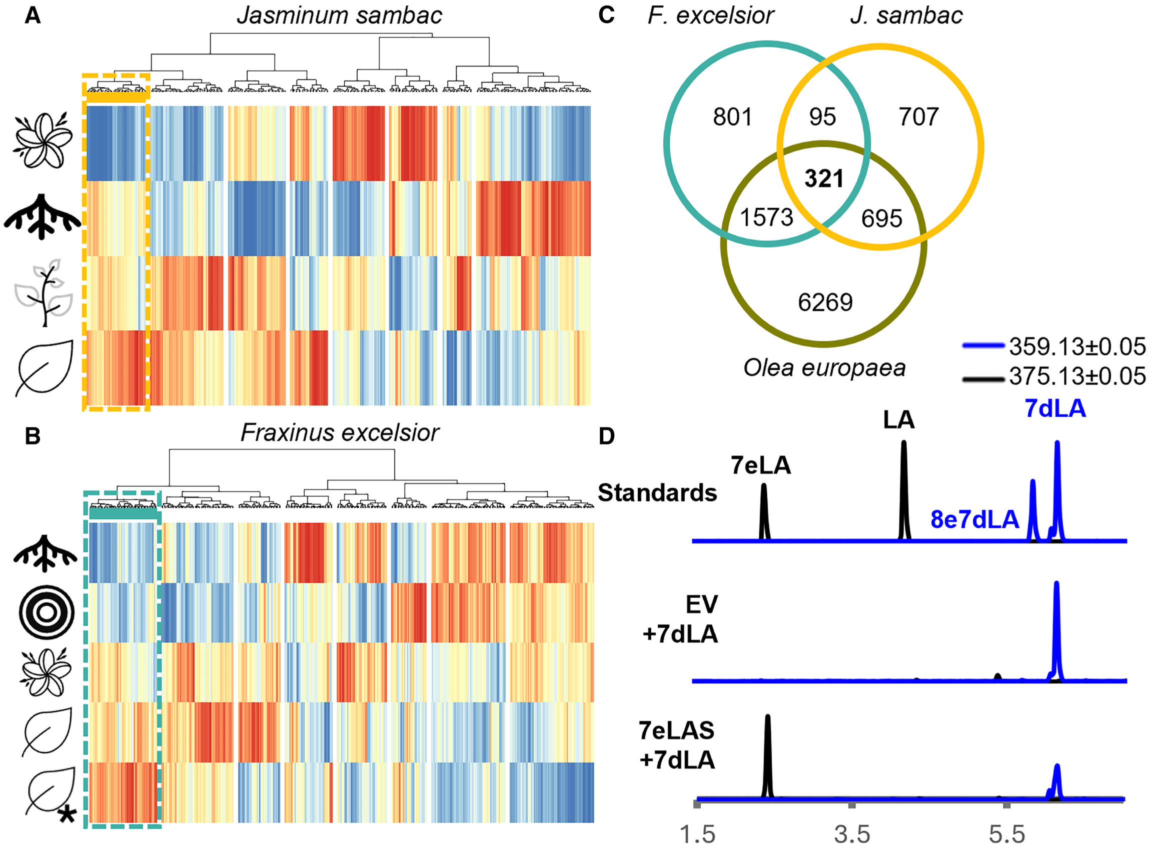

Figure 3. Comparative transcriptomics of Oleaceae species.

(A and B) Heatmaps of the 400 self-organizing map nodes showing the expression patterns of *Jasminum sambac* **(A)** and *Fraxinus excelsior* **(B)** in different tissues, denoted by icons; the selected clusters, in yellow and turquoise, contain candidate biosynthetic genes in the early iridoid pathway. From top to bottom: flower, root, stem, and leaves of *J. sambac* and root, cambium (concentric circles), flower, leaf, and leaf of a selfed tree (leaf + asterisk) of *F. excelsior*.

(C) Venn diagram of orthogroup membership of genes belonging to the clusters of *J. sambac* and *F. excelsior*, and the differentially expressed genes from *Olea europaea* that decreased during ripening; a total of 321 orthogroups are shared among the three gene selections.

(D) Extracted ion chromatogram of the most abundant adducts of loganic acid (LA; $[M–H]^-$) and 7-*epi*-loganic acid (7eLA; $[M–H]^-$) in black (375.1297 ± 0.05) and 7-deoxy-loganic acid (7dLA; $[M–H]^-$) and 8-*epi*-7-deoxy-loganic acid (8e7dLA; $[M–H]^-$) in blue (359.1348 ± 0.05). From top to bottom: mix of standards (Standards) and extracts of *N. benthamiana* leaves co-infiltrated with 7-deoxy-loganic acid and *Agrobacterium* carrying an empty vector (EV + 7dLA) or 7-*epi*-loganic acid synthase (7eLAS + 7dLA).

7-*epi*-loganic acid O-methyltransferase (7eLAMT)

After formation of 7-*epi*-loganic acid, a methyltransferase is predicted to convert this intermediate to 7-*epi*-loganin. Analyzing the resulting orthogroups, we extracted the sequences that belonged to the *C. roseus* LAMT (CrLAMT) orthogroup (OG0000240), which catalyzes the formation of loganin from loganic acid. A phylogenetic analysis (Figure 4) revealed that the olive orthologs of interest clustered within the Lamiales species in a clade adjacent to the Gentianales, where CrLAMT is located. The contig TRINITY_GG_16319_c0_g1_i4 from the *O. europaea* assembly clustered with the Lamiales LAMTs, appeared to be a full-length protein, and was differentially expressed during maturation; it was therefore selected as a likely pathway candidate. When tested *in vitro*, the heterologously expressed, purified protein consumed 7-*epi*-loganic acid to produce 7-*epi*-loganin (Figure 4) and was thus named *O. europaea* 7eLAMT (Oe7eLAMT). Interestingly, the enzyme showed no measurable activity when fed 7-deoxy-loganic acid (Figure 4), which points to a route analogous to that in *C. roseus*, in which 7-deoxy-loganic acid is first oxidized and then methoxylated (Figure 1).

OME glucosyl transferase (OMEGT)

After formation of 7-*epi*-loganin, the previously reported CYP72 OMES catalyzes the formation of OME, which is then converted through an unknown mechanism to ligstroside (Figure 1). Analysis of liquid chromatography–mass spectrometry profiles of olive fruits revealed a chromatographic peak that was highly correlated with 7-*epi*-loganin and secoxyloganin and a fragmentation pattern that matched an iridoid with two hexoses, consistent with literature reports of a glycoside moiety of OME (Kuwajima et al., 1989). We speculated that a glycosylated OME product might be an on-pathway intermediate to oleuropein. To identify the biosynthetic gene responsible for this glucosylation, we gathered genes from among the fruit DEGs that had Pfam annotations of UDP glucosyltransferases. After phylogenetic analysis of these sequences (Figure 5), we searched for UDP-glucose transferases (UGTs) from phylogenetic group L (AtUGT75B1/B2), enzymes that are known to

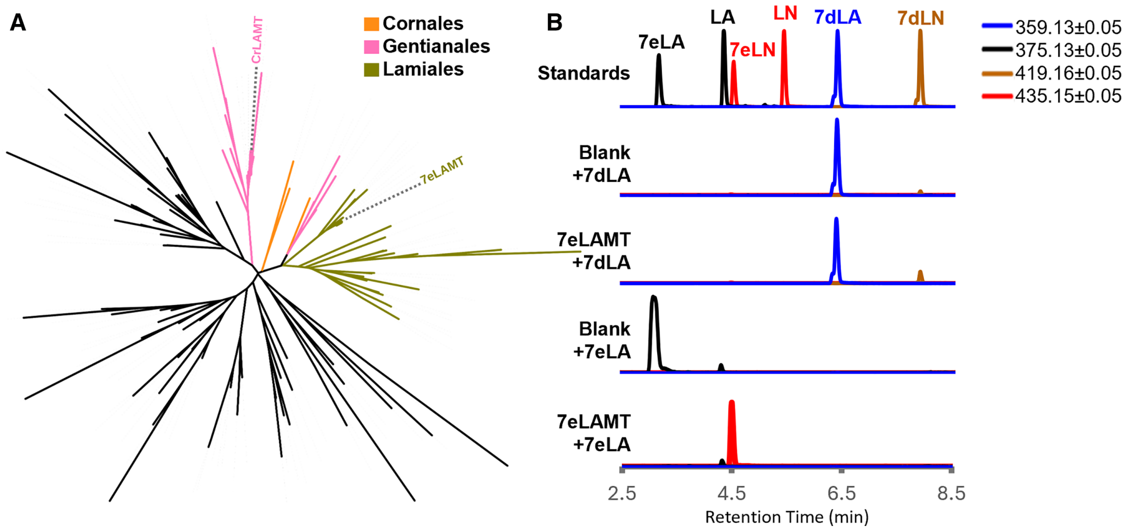

Figure 4. Characterization of 7-*epi*-loganic acid O-methyltransferase (Oe7eLAMT).

(A) Unrooted OrthoFinder tree of group OG0000240, including orthologs of *Catharanthus roseus* loganic acid O-methyltransferase (CrLAMT) from 15 plant species. The clades of the closest orthologs are highlighted according to their corresponding order: Lamiales (olive green), Gentianales (pink), and Cornales (orange).

(B) Extracted ion chromatogram of the most abundant adducts of loganic acid (LA; [M–H]$^-$) and 7-*epi*-loganic acid (7eLA; [M–H]$^-$) in black (375.1297 ± 0.05), loganin (LN; [M+formate]$^-$) and 7-*epi*-loganin (7eLN; [M+formate]$^-$) in red (435.1508 ± 0.05), 7-deoxy-loganic acid (7dLA; [M–H]$^-$) in blue (359.1348 ± 0.05), and 7-deoxy-loganin (7dLN; [M+formate]$^-$) in brown (419.1559 ± 0.05). From top to bottom: mix of standards (Standards), negative control, and purified protein reactions incubated with 7-deoxy-loganic acid (Blank + 7dLA and 7eLAMT + 7dLA, respectively) and with 7-*epi*-loganic acid (Blank + 7eLA and 7eLAMT + 7eLA).

recognize carboxylic groups and catalyze the formation of glucose ester bonds (Caputi et al., 2012). The candidate TRINITY_GG_29808_c0_g1_i1 was selected for heterologous expression in *E. coli*, and *in vitro* testing of the purified protein in the presence of OME and UDP-glucose revealed the production of a compound matching the retention time, *m/z*, and fragmentation pattern of the peak found in olive fruits (Figure 5). To confirm the identity of this product, we performed a large-scale reaction, purified the resulting peak, and confirmed the structure via NMR, identifying the compound as OME-Glc (Supplemental Figures 7–15). We thus named the enzyme *O. europaea* OMEGT (OeOMEGT).

Oleuropein synthase (OS)

Oleuropein is presumed to be produced by oxidation of the hydroxytyrosol moiety of ligstroside, as suggested by labeling experiments in *Syringa josikaea* (Damtoft et al., 1993). It was recently reported that olive PPOs have the capacity to oxidize tyrosol, hydroxytyrosol, and some of its esters (Sánchez et al., 2023; Derardja et al., 2024), but the products of the oxidation reactions of phenolic esters were not reported, and ligstroside was not included in the panel of substrates. We therefore decided to narrow our search to the seven genes in our assembly that were differentially downregulated during ripening and were annotated as PPOs. When *N. benthamiana* leaves were co-infiltrated with ligstroside and *Agrobacterium* harboring the transcripts TRINITY_GG_32073_c0_g1_i1 and TRINITY_GG_25161_c0_g1_i1, oleuropein was detected (Figure 6). These sequences were therefore named OeOS1 and OeOS2 respectively, on the basis of expression levels, and shared only 46.5% amino acid identity. Notably, the sequence of OeOS2 is 99% identical to that of the enzyme OePPO3 reported by Sánchez et al. (2023). We also tested a sequence (TRINITY_GG_32052_c0_g1_i1) with 79.8% amino acid identity to OeOS1, which was reported by Liu et al. (2023) to be syntenic with OeOS1 and probably the result of a recent duplication event. We found this sequence to have detectable OS activity and thus named it OeOS3 (Figure 6).

Pathway reconstitution

The enzyme responsible for conversion of OME or OME-Glc to ligstroside was not identified, despite extensive screening of a wide variety of enzyme candidates. However, we could reconstitute the late-stage intermediate OME-Glc in *N. benthamiana* by transient, sequential expression of 7eLAS, 7eLAMT, OMES, and OMEGT enzymes from olive and feeding with the initial substrate, 7-deoxy-loganic acid. As shown in Figure 7, upon 7-deoxy-loganic acid infiltration, 7eLAS produces 7-*epi*-loganic acid, which is converted into 7-*epi*-loganin when 7eLAMT is added to the mixture; OME is then produced via ketologanin when OMES is included, and finally, OME-Glc is produced by addition of OMEGT, demonstrating that the combination of these enzymes sequentially converts 7-deoxy-loganic acid to OME-Glc (Figure 7).

DISCUSSION

Ripening of olive fruit is a complex process, involving a color change from green to dark purple as well as lipid accumulation and organoleptic changes that make the fruit more palatable for seed dispersers. Our RNA-seq results, consistent with previous reports (reviewed by Skodra et al., 2021), reflect the biology of the olive drupe as a sink tissue, as we see an increase in transcript levels of genes related to receiving carbon in the form of soluble

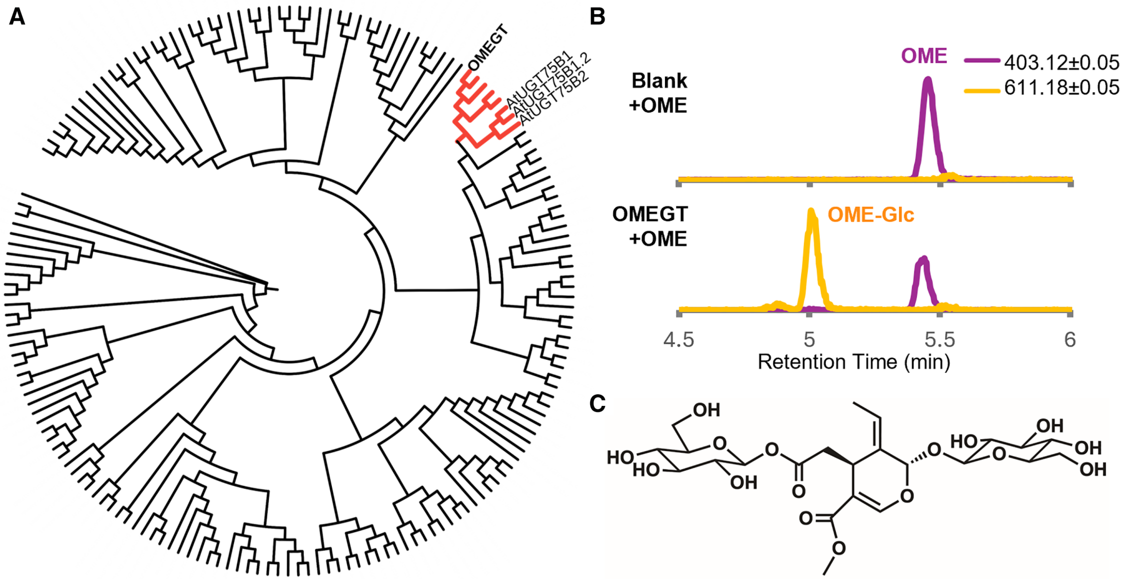

Figure 5. Assays of oleoside-11-methyl ester glucosyl transferase (OMEGT).

(A) Best-fit tree (WAG + F + R6) constructed from a MUSCLE alignment of the protein sequences of *Olea europaea* and *Arabidopsis thaliana* UGTs. In red, we highlight the clade corresponding to the closest olive homologs of AtUGT75 proteins, with the position of the oleoside-11-methyl ester glucosyltransferase (OMEGT) discovered in this article.

(B) Extracted ion chromatogram of the most abundant oleoside-11-methyl ester (OME; purple; $[M–H]^-$ = 403.1246 ± 0.05) and its glucoside adduct (OME-Glc; orange; $[M+formate]^-$ = 611.1829 ± 0.05) from the negative control reaction (Blank + OME; top) and the *in vitro* reaction with purified protein (OMEGT + OME; bottom).

(C) NMR-confirmed structure of the HPLC-purified product of the reaction: 7-β-1-D-glucopyranosyl oleoside-11-methyl ester (OME-Glc).

sugars and rerouting carbon from conversion into starch to fatty acid biosynthesis as the fruit ripens (Supplemental Table 2). At the same time, we observe a decrease in the expression of genes related to chlorophyll biosynthesis, a phenomenon that is partially responsible for the color change from green to purple. Among these changes, enrichment analysis suggested a shift in isoprenoid biosynthesis from the plastidial MEP pathway, decreasing as the fruit matured, to the cytosolic MVA pathway, which increased during the same process (Figure 2; Supplemental Table 3). This was accompanied by a decrease in the accumulation of iridoids, which are derived from geraniol produced via the MEP pathway (Figure 2). We hypothesize that oleuropein accumulation is not only regulated through decreasing expression of biosynthetic genes, from the upstream MEP pathway to the here-discovered OSs (OS1, OS2, and OS3), but also by a decline in oleuropein across cultivars that is likely due to degradation. Nevertheless, in analyses of expression patterns of previously reported hydrolyzing enzymes (Koudounas et al., 2015; Volk et al., 2019), only EAME1, reported by Volk et al. (2019) to act on elenolic acid, the OME aglycone, was negatively correlated with its upstream substrate. Interestingly, Koudounas et al. (2021) reported that silencing of the β-glucosidase paradoxically reduces oleuropein levels 1000-fold, pointing to a more complex role than degradation. However, when these enzymes were tested individually or in conjunction with the enzymes found in this work, none catalyzed the production of ligstroside or oleuropein.

Under the assumption that similar reactions must be catalyzed by homologous enzymes, we discovered 7eLAMT, identified by homology to *C. roseus* LAMT; an OMEGT, aided by homology to *Arabidopsis thaliana* group L UGTs; and two oleoside synthases (OS1 and OS2), annotated as PPOs by protein family homology. However, when testing for homologs of *C. roseus* 7DLH and other transcripts annotated as cytochrome P450s, we did not find the expected activity. We then used a comparative co-expression method that combined guilt-by-association approaches with sequence orthology inference, enabling us to group correlation modules between different species. By analyzing expression data from *J. sambac* and *F. excelsior* and comparing, via orthology, the transcripts that were co-expressed with the known biosynthetic gene homologs, we reduced the number of candidate genes from several thousand to a dozen, enabling the discovery of 7eLAS. However, this method assumes that enzymes are in the same co-regulated module in the analyzed species, which is not always the case. For example, OMEGT and 7eLAMT are co-expressed with the early iridoid biosynthetic genes in *O. europaea* and *F. excelsior* but not in *J. sambac*. As seen in Figure 1, both enzymes would be expected to be present as intermediates in the sambacoside pathway; however, the tissue and variety distribution of sambacoside have not been reported, and it is possible that the sequenced *J. sambac* was not producing sambacoside in the tissues and at the time collected. Notably, in *C. roseus*, LAMT is regulated by ORCA transcription factors, whereas the immediately upstream iridoid biosynthesis from 7DLH to GES is in a different co-regulated module controlled by BIS1 and BIS2 (Van Moerkercke et al., 2015, 2016; Colinas et al., 2021); a similar phenomenon might occur in the case of 7eLAMT in *J. sambac*. Interestingly, none of the reported degradation enzymes were co-expressed across species in the sampled tissues.

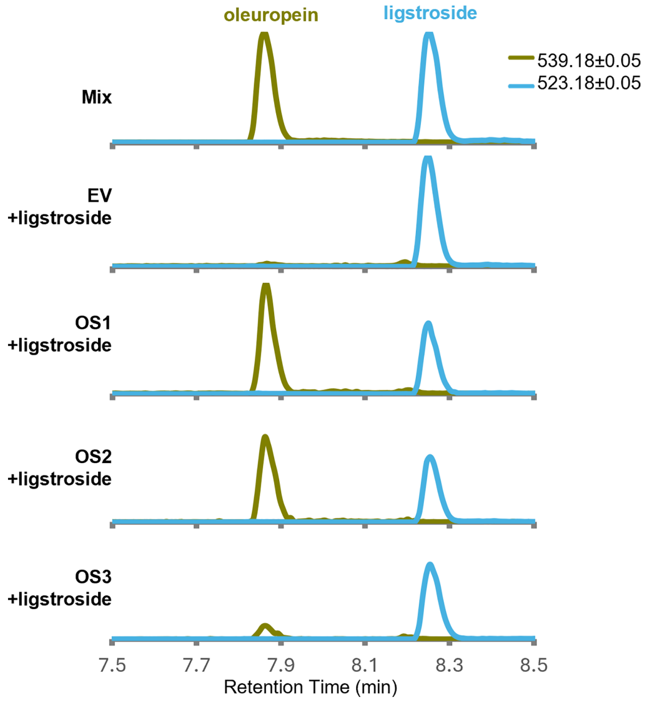

Figure 6. Characterization of oleuropein synthase activity.
The ion chromatogram of the most abundant oleuropein adduct ([M–H]$^-$ = 539.1770 ± 0.05) is shown in olive green, and the most abundant ligstroside adduct ([M–H]$^-$ = 523.1821 ± 0.05) is shown in cornflower blue. From top to bottom: mix of standards and leaf extracts of *Nicotiana benthamiana* co-infiltrated with ligstroside and *Agrobacterium* harboring either an empty vector (EV) or oleuropein synthase 1 (OS1), OS2, or OS3.

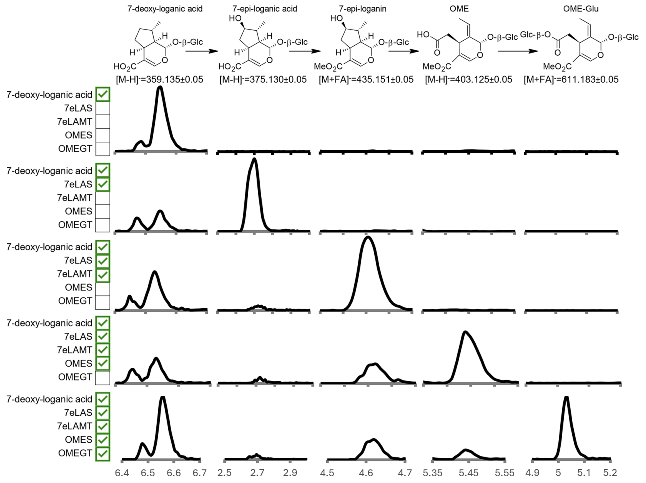

Figure 7. Pathway reconstruction in *Nicotiana benthamiana*.
Extracted ion chromatograms of the most abundant adducts of, from left to right, 7-deoxy-loganic acid ([M–H]$^-$ = 359.1348 ± 0.05), 7-*epi*-loganic acid ([M–H]$^-$ = 375.1297 ± 0.05); 7-*epi*-loganin ([M+formate]$^-$ = 435.1508 ± 0.05); oleoside-11-methyl ester (OME; [M–H]$^-$ = 403.1246 ± 0.05), and 7-β-1-D-glucopyranosyl oleoside-11-methyl ester (OME-Glc; [M+formate]$^-$ = 611.1829 ± 0.05). From top to bottom: extracts of *N. benthamiana* leaves infiltrated with 7-deoxy-loganic acid (7dLA) and *Agrobacterium* containing an empty vector and sequential co-infiltrations of 7-*epi*-loganic acid synthase (7eLAS), 7-*epi*-loganic acid O-methyltransferase (7eLAMT), oleoside-11-methyl ester synthase (OMES), and oleoside-11-methyl ester glucosyl transferase (OMEGT), denoted by green checkmarks to the right of the name. A schematic of the reconstructed biosynthetic module is shown at the top.

We analyzed publicly available RNA-seq data from the cambium of *F. excelsior* clones challenged with the emerald ash borer (Doonan et al., 2023; PRJDB15336; Supplemental Figure 16) and olive subjected to different challenges: stems from two wild olive cultivars with different tolerances to *Verticillium dahliae* (Mascuñano et al., 2025; PRJNA1030405; Supplemental Figure 17), leaves from the olive cultivars Koroneiki and Nocellara del Belice challenged with *Spilocea oleagina* (Marchese et al., 2023; PRJNA929711; Supplemental Figure 18), and xylem of olive cultivars Leccino and Cellina di Nardò (PRJNA780294; Supplemental Figure 19), as well as Ogliarola salentina (Giampetruzzi et al., 2016; PRJNA316374; Supplemental Figure 20) upon infection by *Xylella fastidiosa.* In all cases, similar expression patterns of previously reported biosynthetic genes were observed alongside the enzymes discovered here, supporting a co-regulation hypothesis. An exception was observed in the two wild olive cultivars, in which *7eLAS1*, *OMES*, and *EAME1* genes showed higher basal expression in the susceptible AC15 cultivar, whereas ISY, ISP, 7eLAMT, OMEGT, OS1, OS2, OS3, and, notably, 7eLAS2, showed the opposite pattern (Mascuñano et al., 2025; PRJNA1030405; Supplemental Figure 17). Although these results should be interpreted with caution, as the project differs in tissue type and degree of domestication, they suggest—together with the above-mentioned comparative transcriptomics approach—that the later steps of the pathway branch into different co-regulated modules in different species.

Using the method presented here, we discovered a 2-ODD that catalyzes the stereoselective oxidation of 7-deoxy-loganic acid to 7-*epi*-loganic acid, named 7eLAS. The oxidation of 7-deoxy-loganic acid is a critical junction in the emergence of iridoid diversity within the Oleaceae, as it separates the five monophyletic tribes into three distinct pathways matching likely founder-event speciation, with Myxopyreae and Fontanesieae, the oldest and second oldest tribes to diverge (Dupin et al., 2024), producing loganic acid and loganin (Jensen et al., 2002) with the exact same stereochemistry as that in *C. roseus* (Gentianales). Given the restrictionof oleoside-type secoiridoids to Jasmineae and Oleeae and their position as the hindmost divergent sister tribes in the Oleaceae (Dupin et al., 2024), we posit the hypothesis that the ancestral oxidation of 7-deoxy-loganic acid produced loganic acid, with the production of 7-*epi*-loganic acid (and the oxidation of carbon 10 in Forsythieae) emerging later. In *C. roseus* (Gentianales) and *Camptotheca acuminata* (Cornales), production of loganin is catalyzed by enzymes from the CYP72 family (Salim et al., 2013; Yang et al., 2019). Cytochrome P450s are endoplasmic reticulum–anchored enzymes, dependent on a cytochrome P450 reductase to regenerate their iron catalytic center, and thus have low catalytic efficiencies (Bar-Even and Tawfik, 2013) that, coupled with their low expression in eukaryotic and prokaryotic microbial platforms (Renault et al., 2014; Li et al., 2024), make them one of the bottlenecks in the synthetic reconstruction of plant biosynthetic pathways. On the other hand, ODDs are highly efficient, soluble enzymes, easily expressed in microbial platforms, making them very attractive

for metabolic engineering (Zhao et al., 2025). Unfortunately, few Oleaceae genomes have been reported outside the Oleeae, and the question of whether loganic acid production in Myxopyreae and Fontanesieae is catalyzed by a 7eLAS-like dioxygenase or a 7DLH-like cytochrome P450 enzyme remains open.

Gene discovery is an endeavor with a very low success rate; estimating from the recent completion of the taxol biosynthetic pathway, even with cutting-edge single-nucleus sequencing, the success rate was around 1% for FoTO1 (McClune et al., 2025). Here, we made use of bulk RNA-seq data, within the capability of most laboratories, to reduce the number of candidates to test for a reaction expected to be shared among several species. With this approach, we reduced the number of candidates for the oxidation of 7-deoxy-loganic acid to 12, two of which had the expected activity, a remarkable success rate. Although the approach is not without its drawbacks, particularly the assumption of co-regulation across species, given the increasing availability of expression data across the plant kingdom, this method can facilitate the discovery of novel pathways for plant natural products.

METHODS

Iridoid standards

Loganin, secologanin, ligstroside, oleuropein, and salidroside standards were purchased from Sigma-Aldrich; 8-*epi*-loganin was purchased from AnalytiCon Discovery GmbH (Potsdam, Germany); and OME was purchased from PhytoLab GmbH (Vestenbergsgreuth, Germany). The remaining iridoid standards (7-deoxy-loganic acid, 7-deoxy-8-*epi*-loganic acid, 7-deoxy-loganin, 7-deoxy-8-*epi*-loganic acid, 7-*epi*-loganic acid, 7-*epi*-loganin, and 7-ketologanin) were synthesized from commercially available geniposide (Biosynth Carbosynth, Staad, Switzerland) as reported previously (Rodríguez-López et al., 2021).

Plant material

Olives from Moraiolo, Coratina, Leccino, Arbequina, Tendellone, and Dolce d’Andria cultivars were harvested from the collection fields of CNR IBBR in Perugia (Italy) every 20 days from 45 (stage 1) until 125 (stage 5) days after flowering. Olive fruit mesocarp was frozen in liquid nitrogen, pulverized using a mortar and pestle, and stored at -80°C until needed.

RNA-seq analysis

RNA was extracted from frozen and milled olive fruit mesocarp using the Plant RNeasy Kit (QIAGEN; Hilden, Germany) and sent to BGI Genomics (Shenzhen, China) for RNA-seq following the company’s protocols, which included mRNA enrichment, library preparation, and paired-end sequencing (2×150bp). Raw read quality was assessed using FastQC (v0.11.5; Babraham Bioinformatics, 2016), and the results were aggregated using MultiQC (v1.17; Ewels et al., 2016) and processed using Trimmomatic (Bolger et al., 2014). Trimmed files were mapped against the published genomes of the Farga (Cruz et al., 2016) and Arbequina (Rao et al., 2021) olive cultivars using HISAT2 (v2.2.1; Kim et al., 2019). Genome-guided transcriptome assembly was performed using Trinity (v2.8.5; Grabherr et al., 2011; Haas et al., 2013) with a maximum intron length of 2500 bp. The resulting contigs were cleaned of duplicates using CD-HIT (v4.7; Fu et al., 2012) with a 90% identity threshold, retaining the longest contig, and coding sequences were predicted using TransDecoder (Haas, n.d.). Genes were annotated using the predicted protein sequences and eggNOG-mapper (v2.1.12-1; Huerta-Cepas et al., 2019; Cantalapiedra et al., 2021) with an E-value cutoff of 1×10^{-3}, a threshold score of 60, 40% identity, and 20% coverage, using DIAMOND (Buchfink et al., 2021). Expression was estimated by quasi-mapping the reads using Salmon (v0.14.1; Patro et al., 2017).

The genomes, gene models, and predicted peptide sequences were obtained from published work for *Antirrhinum majus* (Tavares et al., 2018; Li et al., 2019), *Camptotheca acuminata* (Zhao et al., 2017), *Callicarpa americana* (Hamilton et al., 2020), *C. roseus* (Li et al., 2023), *Cinchona pubescens* (Canales et al., 2022), *Gelsemium sempervirens* (Franke et al., 2019), *Mitragyna speciosa* (Brose et al., 2021), *Rauvolfia tetraphylla* (Stander et al., 2023), and *Sesamum indicum* (Wang et al., 2022). For the remaining species, only the genome was used, with no annotation, and the same pipeline described above was applied to generate a genome-guided transcriptome, with the difference being that raw sequencing data were obtained from the Sequence Read Archive (project numbers corresponding to each species are shown in Supplemental Table 4). The genomes used for these species were obtained from GenBank assemblies (GA) and annotated using the BioProject (BP) as follows: *Forsythia suspensa* (GA: GCA_023638005.1; BP: PRJNA793127), *F. excelsior* (GA: GCA_019097785.1; BP: PRJEB4958), *J. sambac* (GA: GCA_018223645.1; BP: PRJNA723725), *Osmanthus fragrans* (GA: GCA_019395295.1; BP: PRJNA529305), and *Penstemon barbatus* (GA: GCA_003313485.2; BP: PRJNA479669). For *J. sambac* and *F. excelsior* only, expression was estimated by quasi-mapping using Salmon (v0.14.1; Patro et al., 2017). Orthogroup inference was performed using OrthoFinder (v2.5.5; Emms and Kelly, 2019), integrating the predicted peptides of all the above-mentioned species. Contextualization of the pathway was performed using RNA-seq runs from projects PRJNA929711 (Marchese et al., 2023), PRJNA1030405 (Mascuñano et al., 2025), PRJNA316374 (Giampetruzzi et al., 2016), and PRJNA780294, and expression was quantified by quasi-mapping against the gene models of the Farga olive genome (Cruz et al., 2016). Similarly, project PRJDB15336 (Doonan et al., 2023) was mapped against our genome-guided transcriptome assembly of *F. excelsior*.

Cloning methods

RNA was extracted from olive fruits using the Plant RNeasy Kit (QIAGEN; Hilden, Germany), and cDNA libraries were prepared using SuperScript IV VILO MM (Thermo Fisher Scientific, Waltham, MA, USA). Candidate genes were amplified using Platinum SuperFi PCR MM (Thermo Fisher Scientific, Waltham, MA, USA) and cloned using ClonExpress II (Vazyme, Nanjing, China) into either pOPINF (OMEGT, 7eLAS) or pOPINM (7eLAMT) for heterologous expression in *E. coli* or directly into the 3Ω1 destination vector for heterologous expression in *N. benthamiana* (Supplemental Table 5). Plasmids were propagated in *E. coli* Top10, and sequences were confirmed by Sanger sequencing (GENEWIZ Germany GmbH, Leipzig, Germany).

Reconstitution in *N. benthamiana*

Sequence-verified plasmids were transformed into *Agrobacterium tumefaciens* GV3101 via electroporation and plated on LB agar with antibiotics (50 $\mu g \cdot ml^{-1}$ rifampicin, 50 $\mu g \cdot ml^{-1}$ gentamicin, and 200 $\mu g \cdot ml^{-1}$ spectinomycin). Single colonies were picked and confirmed by colony PCR using Phire HotStart II Master Mix (Thermo Fisher Scientific, Waltham, MA, USA). Positive colonies were inoculated into liquid LB medium with the above-mentioned antibiotics, grown overnight at 28°C with agitation at 220 rpm in the dark, and pelleted by centrifugation at 5000 RCF for 5 min. The pellet was resuspended in infiltration buffer (50 mM MES [pH 5.5], 10 mM $MgCl_2$, and 200 μM acetosyringone) to reach an optical density (OD_{600}) of 0.6 and incubated in darkness at 28°C and 220 rpm for 2 h. When more than one gene was infiltrated, an equivolumetric mixture was prepared prior to incubation. Three-week-old *N. benthamiana* plants were selected, and the abaxial side of selected leaves was infiltrated with the *Agrobacterium* solution using a needle-less syringe. After 72 h, the substrate was infiltrated in infiltration buffer without acetosyringone, and 96 h later, the infiltrated tissue was isolated, frozen in liquid nitrogen, and extracted with 10 volumes of methanol. The extract was sonicated, centrifuged, and filtered through a 0.45-μm PTFE filter before injection into the high-performance liquid chromatography (HPLC) system for analysis.

Heterologous expression in *E. coli*

For protein production in *E. coli*, sequence-verified plasmids were transformed into *E. coli* BL21 via heat shock transformation and plated on LB medium with carbenicillin (100 μg·ml^{-1}), and single colonies were confirmed by PCR as described above. Positive individual colonies were inoculated into 5 ml liquid LB broth with carbenicillin and incubated overnight at 37°C and 220 rpm. This pre-inoculum was then added to 100 ml fresh YT medium with carbenicillin and incubated at 37°C and 220 rpm, then transferred to 18°C and 220 rpm when an OD_{600} of 0.6 was reached. IPTG was added to a final concentration of 0.5 mM to induce protein expression, and cultures were incubated overnight. Cells were harvested by centrifugation (5000 RCF for 10 min), the pellet was weighed, and protein was extracted using the B-PER Cell Lysis Kit (Thermo Fisher Scientific, Waltham, MA, USA) following the manufacturer's instructions. His-tagged proteins were purified from the clarified solution by incubation with 100 μl Ni-NTA agarose beads (Qiagen) for 1 h at 4°C and eluted using B1 buffer (50 mM Tris–HCl, 50 mM glycine, 500 mM NaCl, and 250 mM imidazole [pH 8.0]). Buffer was exchanged to A4 buffer (20 mM HEPES, 150 mM NaCl, and 10% glycerol [pH 7.5]) by serial dilution using Amicon 30-kDa molecular-weight cutoff concentrators (Millipore). Protein concentration was estimated by measuring absorbance at 280 nm using a NanoDrop spectrophotometer (Thermo Fisher Scientific) and calculating the molar extinction coefficient based on the protein sequence using ExPASy ProtParam (Gasteiger et al., 2005).

Enzymatic assays *in vitro*

To assay methyltransferase activity, purified enzyme was diluted to a final concentration of 0.1 g·l^{-1}, and reactions were performed in 50 μl of 50 mM Tris buffer (pH 8) with freshly added 0.1% (v/v) β-mercaptoethanol, 50 μM ascorbic acid, and 100 μM *S*-adenosyl methionine. To assay UDP-glucosyltransferase activity, 0.1 g·l^{-1} enzyme was incubated with 250 μM UDP-glucose in 50 mM Tris buffer (pH 7.5). For assays to obtain pH optima, the same conditions were maintained, but 50 mM MES buffer was used for lower pH values. Reactions were initiated by addition of 50 μM substrate, incubated at 30°C, and stopped after 2 h by addition of 100 μl ice-cold methanol. To assay dioxygenase activity, 5 μg of purified enzyme was added to 100 μl of reaction buffer containing 25 mM HEPES (pH 7.4), 20 mM ascorbate, 800 μM iron sulfate, 400 μM α-ketoglutarate, and 7 μM 7-deoxy-loganic acid. The reaction was incubated overnight at 30°C and stopped by addition of 100 μl ice-cold methanol. Assays were centrifuged and filtered through a 0.45-μm PTFE filter prior to HPLC injection.

Identification of 7-β-1-D-glucopyranosyl oleoside-11-methyl ester (OME-Glc)

His-tagged enzyme was produced in *E. coli* in a large 1-l batch and purified using Ni-agarose columns in an ÄKTA FPLC system (Cytiva). A large-volume reaction was performed by setting 100 parallel 200-ml reactions in 50 mM MES (pH 5) and was subjected to semi-preparative HPLC for product isolation. An Agilent 1260 Infinity II HPLC instrument was connected to an autosampler, diode array detector, and fraction collector for compound detection and isolation. Chromatographic separation was performed using a Phenomenex Kinetex XB-C18 column (5.0 μm, 100 Å, 100 × 2.1 mm) maintained at 40°C under gradient elution using reversed-phase conditions. The mobile phases used for separation were water with 0.1% formic acid (A) and acetonitrile (B). The flow rate was set to 1.5 ml·min^{-1}, and chromatographic separation was performed at 5% B for 2 min, followed by a linear gradient from 5% to 10% B over 12 min, 90% B for 3 min, and 10% B for 3 min (t_{total} = 20 min). Prior to injection, the samples were diluted to 1 mg·ml^{-1} with methanol and filtered using a 0.22-μm PTFE syringe filter. The diluted samples were placed in the autosampler, 20-μL injections were performed, and fractions were collected by monitoring UV absorbance at 254 and 238 nm. Fractions were pooled and evaporated to dryness. The isolated compound was then submitted for NMR analysis.

NMR characterization

NMR spectra of enzymatically generated 7-glucopyranosyl OME were measured with a 700 MHz Bruker Avance III HD spectrometer (Bruker BioSpin GmbH, Rheinstetten, Germany) equipped with a TCI cryoprobe using standard pulse sequences as implemented in Bruker TopSpin (v3.6.1; Bruker BioSpin GmbH, Rheinstetten, Germany) at 298 K. Chemical shifts were referenced to the residual solvent signals of MeOH-d_3 (δ_H 3.31/δ_C 49.0). The assignments and spectra are shown in Supplemental Figures 7–15. The chemical shifts agreed with published data (Kuwajima et al., 1989).

Metabolite profiling using HPLC–MS

Metabolite profiling was performed as described previously (Rodríguez-López et al., 2021), with minor modifications. Samples were chromatographically separated using a Thermo UltiMate 3000 UHPLC system (Thermo Fisher Scientific) equipped with an Acquity UPLC BEH C18 column (2.1 × 50 mm, 1.7 μm, 100 Å; Waters) coupled via pneumatic-assisted ESI to an Impact II q-TOF mass spectrometer (Bruker Daltonik). Iridoids were separated at 40°C using a gradient from 0.1% formic acid in water to acetonitrile, following previously reported gradients (Rodríguez-López et al., 2021, 2022). The output was ionized in negative mode, with a capillary voltage of 3.5 kV, a nebulizer pressure of 2.5 bar, and nitrogen as the drying gas (flow rate of 11 l·min^{-1}, 350°C). Data-dependent fragmentation was triggered at an absolute threshold of 400 and acquired for the most intense peaks, which were excluded after three events, with dynamic collision energy from 20 to 50 eV. Raw mass spectrometry (MS) files were converted to mzXML using Bruker Data Analysis software (Bruker Daltonik, Bremen, Germany). When needed, extracted ion chromatograms were exported to CSV format using MZmine2 (v2.40.1) (Pluskal et al., 2010), and chromatograms were plotted using Microsoft Excel.

For *in vitro* assays of 7eLAS, UHPLC–HRMS analysis was performed using a Vanquish UHPLC system (Thermo Fisher Scientific) with a Waters Acquity UPLC BEH C18 column (2.1 × 50 mm, 1.7 μm, 130 Å). Iridoids were separated at 40°C with mobile phases consisting of Milli-Q water with 0.1% formic acid (A) and acetonitrile (B). The 7-min gradient consisted of a linear increase from 1% to 50% B over 5 min. The wash stage was set to 100% B for 0.5 min before switching back to 1% B for 1.5 min to condition the column for the next injection. The flow rate was 0.6 ml min^{-1}, the injection volume was 2 μl, and the sample tray was kept at 10°C. MS data acquisition was performed on a Q Exactive Plus Orbitrap mass spectrometer (Thermo Fisher Scientific) with full MS/dd-MS2 in negative ionization mode over the mass range *m/z* 120–1000. Source parameters were set to 3.5 kV, sheath gas flow rate 55, auxiliary gas flow rate 15, and capillary temperature 275°C. For full MS, the resolution was 70 000, the AGC target was set to 2e5, and the maximum IT was set to 100 ms. The parameters for dd-MS2 were as follows: resolution 35 000, mass isolation window 0.7 Da, AGC target 1e5, and maximum IT 100 ms. Normalized collision energy was set to three levels: 20%, 40%, and 60%. Spectral data were acquired in centroid mode. All parameters of the UHPLC–HRMS system were controlled using Xcalibur software version 4.3.73.11 (Thermo Fisher Scientific). Chromatographic peak areas from extracted ion chromatograms were integrated and extracted using Xcalibur Quan Browser version 4.3.73.11 (Thermo Fisher Scientific).

Data analysis

Unless otherwise specified, data analysis was performed using the *base* library of the R programming language (v4.4.1, R Core Team). Figures were generated with the aid of the *ggplot2* (Wickham, 2016), *gplots* (Warnes et al., 2009), and *pheatmap* (Kolde, 2019) libraries, and Venn diagrams were produced using the *VennDiagram* package (Chen and Boutros, 2011). Differential expression analysis, including count normalization, was performed using the likelihood ratio test option in DESeq2 (Love et al., 2014), and SOMs were created using the *kohonen* library (Wehrens and Kruisselbrink, 2018) as reported previously

(Rodríguez-López et al., 2022). The *seqinr* package (Charif and Lobry, 2007) was used to handle nucleotide and peptide sequences, and *ape* (Paradis et al., 2004) was used for tree handling and phylogenetic analyses. The UGT tree was generated from a multiple sequence alignment created in MUSCLE (Edgar, 2004; Madeira et al., 2024) and inferred using ModelFinder (Kalyaanamoorthy et al., 2017) via IQ-TREE (Nguyen et al., 2015). The LAMT orthogroup tree was selected from the OrthoFinder standard output results. Both trees were plotted using iTOL (v6; Letunic and Bork, 2024). Extracted ion chromatograms were generated from raw files using MZmine2 (Pluskal et al., 2010) and plotted using Excel; molecules were drawn using ChemDraw.

DATA AND CODE AVAILABILITY

Raw RNA-seq data will be available in the European Nucleotide Archive (ENA) at the European Bioinformatics Institute (EBI) under accession number PRJEB87345. The sequences of enzymes identified in this study are available in GenBank under the following accession numbers: 7eLAS1 (PV366771), 7eLAS2 (PV366772), 7eLAMT (PV358384), OMEGT (PV358385), OS1 (PV358386), OS2 (PV358387), and OS3 (PV358388). All code generated for this study is available at https://github.com/crdzl/olive_7eLAS.

FUNDING

C.E.R.-L. acknowledges the support of the School of Engineering and Sciences at Tecnológico de Monterrey for funding travel and lodging during his short research stay at the Max Planck Institute for Chemical Ecology. O.C. was supported by the short-term mobility program of the National Research Council (CNR), Italy, during her stay at the SOC Department of ICE. O.C. also acknowledges financial support from the ALIFUN project (ARS01_00783) and the PRIMA project BiomeNext (J63C21000100006), both funded by the Italian Ministry of University and Research.

ACKNOWLEDGMENTS

We thank Maritta Kunert and Matilde Florean from the Department of Natural Products Biosynthesis at the Max Planck Institute for Chemical Ecology for their assistance with mass spectrometry and pathway reconstitution in *Nicotiana benthamiana*. We also acknowledge Luciana Baldoni (CNR IBBR, Italy) for assistance with olive germplasm selection and for valuable suggestions during the development of this work. No conflict of interest is declared.

AUTHOR CONTRIBUTIONS

O.C., S.E.O'C., L.C., and C.E.R.-L. designed the experiments and wrote the manuscript; C.E.R.-L., Y.J., O.C., and M.O.K. identified, cloned, and characterized the enzymes; B.H. and R.M.A. synthesized standards and substrates; M.O.K., S.H., O.C., and Y.N. purified and determined the structure of OME-Glc; C.E.R.-L., A.G.-V., F.A., and E.F. performed the bioinformatics analyses; F.P., M.C.V., O.C., and S.M. provided plant material and extracted RNA and metabolites for transcriptomic and metabolomic analyses; O.C. and M.O.K. performed infiltrations and reconstituted the pathway in *Nicotiana benthamiana*. All authors reviewed the manuscript.

SUPPLEMENTAL INFORMATION

Supplemental information is available at *Plant Communications Online*.

Received: June 4, 2025
Revised: November 28, 2025
Accepted: January 7, 2026
Published: January 8, 2026

REFERENCES

Alagna, F., Mariotti, R., Panara, F., Caporali, S., Urbani, S., Veneziani, G., Esposto, S., Taticchi, A., Rosati, A., Rao, R., et al. (2012). Olive Phenolic Compounds: Metabolic and Transcriptional Profiling during Fruit Development. BMC Plant Biol. **12**:162. https://doi.org/10.1186/1471-2229-12-162.

Amiot, M.J., Fleuriet, A., and Macheix, J.J. (1986). Importance and Evolution of Phenolic Compounds in Olive during Growth and Maturation. J. Agric. Food Chem. **34**:823–826. https://doi.org/10.1021/jf00071a014.

Babraham Bioinformatics. 2016. *FastQC*. V. 0.11.5. Released. https://www.bioinformatics.babraham.ac.uk/projects/fastqc/.

Bar-Even, A., and Tawfik, D.S. (2013). Engineering Specialized Metabolic Pathways—Is There a Room for Enzyme Improvements? Food Biotechnol. Plant Biotechnol. **24**:310–319. https://doi.org/10.1016/j.copbio.2012.10.006.

Boachon, B., Robin Buell, C., Crisovan, E., Dudareva, N., Garcia, N., Godden, G., Henry, L., Kamileen, M.O., Kates, H.R., Kilgore, M.B., et al. (2018). Phylogenomic Mining of the Mints Reveals Multiple Mechanisms Contributing to the Evolution of Chemical Diversity in Lamiaceae. Mol. Plant **11**:1084–1096. https://doi.org/10.1016/j.molp.2018.06.002.

Bolger, A.M., Lohse, M., and Usadel, B. (2014). Trimmomatic: A Flexible Trimmer for Illumina Sequence Data. Bioinformatics **30**:2114–2120. https://doi.org/10.1093/bioinformatics/btu170.

Brose, J., Lau, K.H., Dang, T.T.T., Hamilton, J.P., Martins, L.d.V., Hamberger, B., Hamberger, B., Jiang, J., O'Connor, S.E., and Buell, C.R. (2021). The Mitragyna Speciosa (Kratom) Genome: A Resource for Data-Mining Potent Pharmaceuticals That Impact Human Health. G3 (Bethesda). **11**:jkab058. https://doi.org/10.1093/g3journal/jkab058.

Buchfink, B., Reuter, K., and Drost, H.-G. (2021). Sensitive Protein Alignments at Tree-of-Life Scale Using DIAMOND. Nat. Methods **18**:366–368. https://doi.org/10.1038/s41592-021-01101-x.

Cantalapiedra, C.P., Hernández-Plaza, A., Letunic, I., Bork, P., and Huerta-Cepas, J. (2021). eggNOG-Mapper v2: Functional Annotation, Orthology Assignments, and Domain Prediction at the Metagenomic Scale. Mol. Biol. Evol. **38**:5825–5829. https://doi.org/10.1093/molbev/msab293.

Caputi, L., Malnoy, M., Goremykin, V., Nikiforova, S., and Martens, S. (2012). A Genome-Wide Phylogenetic Reconstruction of Family 1 UDP-Glycosyltransferases Revealed the Expansion of the Family during the Adaptation of Plants to Life on Land. Plant J. **69**:1030–1042. https://doi.org/10.1111/j.1365-313X.2011.04853.x.

Canales, N.A., Pérez-Escobar, O.A., Powell, R.F., Töpel M, Kidner, C., Nesbitt, M., Maldonado, C., Barnes, C.J., Rønsted, N., Przelomska, N.A.S., Leitch, I.J., et al. (2022). A highly contiguous, scaffold-level nuclear genome assembly for the fever tree (Cinchona pubescens Vahl) as a novel resource for Rubiaceae research. Gigabyte **2022**:gigabyte71. https://doi.org/10.46471/gigabyte.71.

Charif, D., and Lobry, J.R. (2007). SeqinR 1.0-2: A Contributed Package to the R Project for Statistical Computing Devoted to Biological Sequences Retrieval and Analysis. In Structural Approaches to Sequence Evolution: Molecules, Networks, Populations, U. Bastolla, M. Porto, H. Eduardo Roman, and Michele Vendruscolo, eds. (Springer Berlin Heidelberg). https://doi.org/10.1007/978-3-540-35306-5_10.

Chen, H., and Boutros, P.C. (2011). VennDiagram: A Package for the Generation of Highly-Customizable Venn and Euler Diagrams in R. BMC Bioinf. **12**:35. https://doi.org/10.1186/1471-2105-12-35.

Colinas, M., Pollier, J., Vaneechoutte, D., Malat, D.G., Schweizer, F., De Milde, L., De Clercq, R., Guedes, J.G., Martínez-Cortés, T., Molina-Hidalgo, F.J., et al. (2021). Subfunctionalization of Paralog Transcription Factors Contributes to Regulation of Alkaloid Pathway Branch Choice in Catharanthus Roseus. Front. Plant Sci. **12**:687406. https://doi.org/10.3389/fpls.2021.687406.

Colinas, M., Tymen, C., Wood, J.C., David, A., Wurlitzer, J., Morweiser, C., Gase, K., Alam, R.M., Titchiner, G.R., Hamilton, J.P., et al. (2025). Discovery of Iridoid Cyclase Completes the Iridoid Pathway in Asterids. Nat. Plants **11**:2204–2216. https://doi.org/10.1038/s41477-025-02122-6.

Contin, A., van der Heijden, R., Lefeber, A.W., and Verpoorte, R. (1998). The Iridoid Glucoside Secologanin Is Derived from the Novel Triose Phosphate/Pyruvate Pathway in a Catharanthus Roseus Cell Culture. FEBS Lett. **434**:413–416. https://doi.org/10.1016/S0014-5793(98)01022-9.

Cruz, F., Julca, I., Gómez-Garrido, J., Loska, D., Marcet-Houben, M., Cano, E., Galán, B., Frias, L., Ribeca, P., Derdak, S., et al. (2016). Genome Sequence of the Olive Tree, Olea Europaea. GigaScience **5**:29. https://doi.org/10.1186/s13742-016-0134-5.

Damtoft, S., Franzyk, H., and Jensen, S.R. (1992). Excelsioside, a Secoiridoid Glucoside from Fraxinus Excelsior. Phytochemistry **31**:4197–4201. https://doi.org/10.1016/0031-9422(92)80442-H.

Damtoft, S., Franzyk, H., and Jensen, S.R. (1993). Biosynthesis of Secoiridoid Glucosides in Oleaceae. Phytochemistry **34**:1291–1299. https://doi.org/10.1016/0031-9422(91)80018-V.

Derardja, A.eddine, Pretzler, M., Barkat, M., and Rompel, A. (2024). Extraction, Purification, and Characterization of Olive (Olea Europaea L., Cv. Chemlal) Polyphenol Oxidase. J. Agric. Food Chem. **72**:3099–3112. https://doi.org/10.1021/acs.jafc.3c07776.

Diccionario de La Lengua Española. n.d. "Aceite." 23.8. Real Academia Española. Accessed February 19, 2026. https://dle.rae.es/aceite.

Doonan, J.M., Kosawang, C., Eisenring, M., Ladd, T., Roe, A.D., Budde, K.B., Jørgensen, H.J.L., Queloz, V., Gossner, M.M., and Nielsen, L.R. (2023). Transcriptome Profiling of Fraxinus Excelsior Genotypes Infested by Emerald Ash Borer. Sci. Data **10**:680. https://doi.org/10.1038/s41597-023-02588-z.

Dupin, J., Hong-Wa, C., Gaudeul, M., and Besnard, G. (2024). Phylogenetics and Biogeography of the Olive Family (Oleaceae). Ann. Bot. **134**:577–592. https://doi.org/10.1093/aob/mcae100.

Edgar, R.C. (2004). MUSCLE: Multiple Sequence Alignment with High Accuracy and High Throughput. Nucleic Acids Res. **32**:1792–1797. https://doi.org/10.1093/nar/gkh340.

Emms, D.M., and Kelly, S. (2019). OrthoFinder: Phylogenetic Orthology Inference for Comparative Genomics. Genome Biol. **20**:238. https://doi.org/10.1186/s13059-019-1832-y.

Ewels, P., Magnusson, M., Lundin, S., and Käller, M. (2016). MultiQC: Summarize Analysis Results for Multiple Tools and Samples in a Single Report. Bioinformatics **32**:3047–3048. https://doi.org/10.1093/bioinformatics/btw354.

Franke, J., Kim, J., Hamilton, J.P., Zhao, D., Pham, G.M., Wiegert-Rininger, K., Crisovan, E., Newton, L., Vaillancourt, B., Tatsis, E., et al. (2019). Gene Discovery in Gelsemium Highlights Conserved Gene Clusters in Monoterpene Indole Alkaloid Biosynthesis. Chembiochem **20**:83–87. https://doi.org/10.1002/cbic.201800592.

Fu, L., Niu, B., Zhu, Z., Wu, S., and Li, W. (2012). CD-HIT: Accelerated for Clustering the next-Generation Sequencing Data. Bioinformatics **28**:3150–3152. https://doi.org/10.1093/bioinformatics/bts565.

Gasteiger, E., Hoogland, C., Gattiker, A., Duvaud, S., Wilkins, M.R., Appel, R.D., and Bairoch, A. (2005). Protein Identification and Analysis Tools on the ExPASy Server. In The Proteomics Protocols Handbook, J.M. Walker, ed. (Humana Press). https://doi.org/10.1385/1-59259-890-0:571.

Giampetruzzi, A., Morelli, M., Saponari, M., Loconsole, G., Chiumenti, M., Boscia, D., Savino, V.N., Martelli, G.P., and Saldarelli, P. (2016). Transcriptome Profiling of Two Olive Cultivars in Response to Infection by the CoDiRO Strain of Xylella Fastidiosa Subsp. Pauca. BMC Genom. **17**:475. https://doi.org/10.1186/s12864-016-2833-9.

Grabherr, M.G., Haas, B.J., Yassour, M., Levin, J.Z., Thompson, D.A., Amit, I., Adiconis, X., Fan, L., Raychowdhury, R., Zeng, Q., et al. (2011). Full-Length Transcriptome Assembly from RNA-Seq Data without a Reference Genome. Nat. Biotechnol. **29**:644–652. https://doi.org/10.1038/nbt.1883.

Haas, B.. n.d. *TransDecoder*. V. 5.6.0. https://github.com/TransDecoder/TransDecoder.

Haas, B.J., Papanicolaou, A., Yassour, M., Grabherr, M., Blood, P.D., Bowden, J., Couger, M.B., Eccles, D., Li, B., Lieber, M., et al. (2013). De Novo Transcript Sequence Reconstruction from RNA-Seq Using the Trinity Platform for Reference Generation and Analysis. Nat. Protoc. **8**:1494–1512. https://doi.org/10.1038/nprot.2013.084.

Hamilton, J.P., Godden, G.T., Lanier, E., Bhat, W.W., Kinser, T.J., Vaillancourt, B., Wang, H., Wood, J.C., Jiang, J., Soltis, P.S., et al. (2020). Generation of a Chromosome-Scale Genome Assembly of the Insect-Repellent Terpenoid-Producing Lamiaceae Species, Callicarpa Americana. GigaScience **9**:giaa093. https://doi.org/10.1093/gigascience/giaa093.

Hoad, T.F. (2003). The Concise Oxford Dictionary of English Etymology (Oxford University Press). https://doi.org/10.1093/acref/9780192830982.001.0001.

Huerta-Cepas, J., Szklarczyk, D., Heller, D., Hernández-Plaza, A., Forslund, S.K., Cook, H., Mende, D.R., Letunic, I., Rattei, T., Jensen, L.J., et al. (2019). eggNOG 5.0: A Hierarchical, Functionally and Phylogenetically Annotated Orthology Resource Based on 5090 Organisms and 2502 Viruses. Nucleic Acids Res. **47**:D309–D314. https://doi.org/10.1093/nar/gky1085.

Jensen, S.R., Franzyk, H., and Wallander, E. (2002). Chemotaxonomy of the Oleaceae: Iridoids as Taxonomic Markers. Phytochemistry **60**:213–231. https://doi.org/10.1016/S0031-9422(02)00102-4.

Kalyaanamoorthy, S., Minh, B.Q., Wong, T.K.F., von Haeseler, A., and Jermiin, L.S. (2017). ModelFinder: Fast Model Selection for Accurate Phylogenetic Estimates. Nat. Methods **14**:587–589. https://doi.org/10.1038/nmeth.4285.

Kim, D., Paggi, J.M., Park, C., Bennett, C., and Salzberg, S.L. (2019). Graph-Based Genome Alignment and Genotyping with HISAT2 and HISAT-Genotype. Nat. Biotechnol. **37**:907–915. https://doi.org/10.1038/s41587-019-0201-4.

Kolde, R.. 2019. *Pheatmap: Pretty Heatmaps*. Released. https://github.com/raivokolde/pheatmap.

Koudounas, K., Banilas, G., Michaelidis, C., Demoliou, C., Rigas, S., and Hatzopoulos, P. (2015). A Defence-Related Olea Europaea β-Glucosidase Hydrolyses and Activates Oleuropein into a Potent Protein Cross-Linking Agent. J. Exp. Bot. **66**:2093–2106. https://doi.org/10.1093/jxb/erv002.

Koudounas, K., Thomopoulou, M., Rigakou, A., Angeli, E., Melliou, E., Magiatis, P., and Hatzopoulos, P. (2021). Silencing of Oleuropein β-Glucosidase Abolishes the Biosynthetic Capacity of Secoiridoids in Olives. Front. Plant Sci. **12**:671487. https://doi.org/10.3389/fpls.2021.671487.

Kuwajima, H., Matsuuchi, K., Takaishi, K., Inoue, K., Fujita, T., and Inouye, H. (1989). A Secoiridoid Glucoside from Ligustrum Japonicum. Phytochemistry **28**:1409–1411. https://doi.org/10.1016/S0031-9422(00)97757-4.

Letunic, I., and Bork, P. (2024). Interactive Tree of Life (iTOL) v6: Recent Updates to the Phylogenetic Tree Display and Annotation Tool. Nucleic Acids Res. **52**:W78–W82. https://doi.org/10.1093/nar/gkae268.

Li, C., Wood, J.C., Vu, A.H., Hamilton, J.P., Rodriguez Lopez, C.E., Payne, R.M.E., Serna Guerrero, D.A., Gase, K., Yamamoto, K., Vaillancourt, B., et al. (2023). Single-Cell Multi-Omics in the Medicinal Plant Catharanthus Roseus. Nat. Chem. Biol. **19**:1031–1041. https://doi.org/10.1038/s41589-023-01327-0.

Li, M., Zhang, D., Gao, Q., Luo, Y., Zhang, H., Ma, B., Chen, C., Whibley, A., Zhang, Y., Cao, Y., et al. (2019). Genome Structure and Evolution of Antirrhinum Majus L. Nat. Plants **5**:174–183. https://doi.org/10.1038/s41477-018-0349-9.

Li, Y., Li, J., Chen, W.-K., Li, Y., Xu, S., Li, L., Xia, B., and Wang, R. (2024). Tuning Architectural Organization of Eukaryotic P450 System to Boost Bioproduction in Escherichia Coli. Nat. Commun. **15**:10009. https://doi.org/10.1038/s41467-024-54259-1.

Liu, Q., Wang, C., Cui, Q., Fan, Y., Zhang, J., and Rao, G. (2023). Genome-Wide Analysis of the Polyphenol Oxidase Gene Family in Olea Europaea Provides Insights into the Mechanism of Enzymatic Browning in Olive Fruit. Antioxidants **12**:1661. https://doi.org/10.3390/antiox12091661.

Love, M.I., Huber, W., and Anders, S. (2014). Moderated Estimation of Fold Change and Dispersion for RNA-Seq Data with DESeq2. Genome Biol. **15**:550. https://doi.org/10.1186/s13059-014-0550-8.

Madeira, F., Madhusoodanan, N., Lee, J., Eusebi, A., Niewielska, A., Tivey, A.R.N., Lopez, R., and Butcher, S. (2024). The EMBL-EBI Job Dispatcher Sequence Analysis Tools Framework in 2024. Nucleic Acids Res. **52**:W521–W525. https://doi.org/10.1093/nar/gkae241.

Marchese, A., Balan, B., Antonina Trippa, D., Bonanno, F., Caruso, T., Imperiale, V., Marra, F.P., and Giovino, A. (2023). NGS Transcriptomic Analysis Uncovers the Possible Resistance Mechanisms of Olive to Spilocea Oleagina Leaf Spot Infection. Front. Plant Sci. **14**:2023. https://www.frontiersin.org/journals/plant-science/articles/10.3389/fpls.2023.1219580.

Mascuñano, B., Coto-Elena, J., Guerrero-Sánchez, V.M., Paniagua, C., Blanco-Portales, R., Caballero, J.L., Trapero-Casas, J.L., Jiménez-Díaz, R.M., Pliego-Alfaro, F., Mercado, J.A., et al. (2025). Transcriptome Analysis of Wild Olive (Olea Europaea L. Subsp. Europaea Var. Sylvestris) Clone AC18 Provides Insight into the Role of Lignin as a Constitutive Defense Mechanism Underlying Resistance to Verticillium Wilt. BMC Plant Biol. **25**:292. https://doi.org/10.1186/s12870-025-06301-7.

McClune, C.J., Liu, J.C.-T., Wick, C., De La Peña, R., Lange, B.M., Fordyce, P.M., and Sattely, E.S. (2025). Discovery of FoTO1 and Taxol Genes Enables Biosynthesis of Baccatin III. Nature **643**:582–592. https://doi.org/10.1038/s41586-025-09090-z.

Mousavi, S., Stanzione, V., Mariotti, R., Mastio, V., Azariadis, A., Passeri, V., Valeri, M.C., Baldoni, L., and Bufacchi, M. (2022). Bioactive Compound Profiling of Olive Fruit: The Contribution of Genotype. Antioxidants **11**:672. https://doi.org/10.3390/antiox11040672.

Nguyen, L.-T., Schmidt, H.A., von Haeseler, A., and Minh, B.Q. (2015). IQ-TREE: A Fast and Effective Stochastic Algorithm for Estimating Maximum-Likelihood Phylogenies. Mol. Biol. Evol. **32**:268–274. https://doi.org/10.1093/molbev/msu300.

Paradis, E., Claude, J., and Strimmer, K. (2004). APE: Analyses of Phylogenetics and Evolution in R Language. Bioinformatics **20**:289–290. https://doi.org/10.1093/bioinformatics/btg412.

Patro, R., Duggal, G., Love, M.I., Irizarry, R.A., and Kingsford, C. (2017). Salmon Provides Fast and Bias-Aware Quantification of Transcript Expression. Nat. Methods **14**:417–419. https://doi.org/10.1038/nmeth.4197.

Pluskal, T., Castillo, S., Villar-Briones, A., and Orešič, M. (2010). MZmine 2: Modular Framework for Processing, Visualizing, and Analyzing Mass Spectrometry-Based Molecular Profile Data. BMC Bioinf. **11**:395. https://doi.org/10.1186/1471-2105-11-395.

Rao, G., Zhang, J., Liu, X., Lin, C., Xin, H., Xue, L., and Wang, C. (2021). De Novo Assembly of a New Olea Europaea Genome Accession Using Nanopore Sequencing. Hortic. Res. **8**:64. https://doi.org/10.1038/s41438-021-00498-y.

Renault, H., Bassard, J.-E., Hamberger, B., and Werck-Reichhart, D. (2014). Cytochrome P450-Mediated Metabolic Engineering: Current Progress and Future Challenges. Curr. Opin. Plant Biol. **19**:27–34. https://doi.org/10.1016/j.pbi.2014.03.004.

Rodríguez-López, C.E., Hong, B., Paetz, C., Nakamura, Y., Koudounas, K., Passeri, V., Baldoni, L., Alagna, F., Calderini, O., and O'Connor, S.E. (2021). Two Bi-Functional Cytochrome P450 CYP72 Enzymes from Olive (Olea Europaea) Catalyze the Oxidative C-C Bond Cleavage in the Biosynthesis of Secoxy-Iridoids – Flavor and Quality Determinants in Olive Oil. New Phytol. **229**:2288–2301. https://doi.org/10.1111/nph.16975.

Rodríguez-López, C.E., Jiang, Y., Kamileen, M.O., Lichman, B.R., Hong, B., Vaillancourt, B., Buell, C.R., and O'Connor, S.E. (2022). Phylogeny-Aware Chemoinformatic Analysis of Chemical Diversity in Lamiaceae Enables Iridoid Pathway Assembly and Discovery of Aucubin Synthase. Mol. Biol. Evol. **39**:msac057. https://doi.org/10.1093/molbev/msac057.

Ross, S.A., El-Sayyad, S.M., Ali, A.A., and El-Keltawy, N.E. (1982). Phytochemical Studies on Jasminum Sambac. Fitoterapia **53**:91–95.

Ryan, D., Robards, K., and Lavee, S. (1999). Changes in Phenolic Content of Olive during Maturation. Int. J. Food Sci. Technol. **34**:265–274. https://doi.org/10.1046/j.1365-2621.1999.00261.x.

Salim, V., Fang, Y., Altarejos, J., and De Luca, V. (2013). Virus-Induced Gene Silencing Identifies Atharanthus Roseus 7-Deoxyloganic Acid-7-Hydroxylase, a Step in Iridoid and Monoterpene Indole Alkaloid Biosynthesis. Plant J. **76**:754–765. https://doi.org/10.1111/tpj.12330.

Sánchez, R., Arroyo, L., Luaces, P., Sanz, C., and Pérez, A.G. (2023). Olive Polyphenol Oxidase Gene Family. Int. J. Mol. Sci. **24**:3233. https://doi.org/10.3390/ijms24043233.

Skodra, C., Titeli, V.S., Michailidis, M., Bazakos, C., Ganopoulos, I., Molassiotis, A., and Tanou, G. (2021). Olive Fruit Development and Ripening: Break on through to the '-Omics' Side. Int. J. Mol. Sci. **22**:5806. https://doi.org/10.3390/ijms22115806.

Stander, E.A., Lehka, B., Carqueijeiro, I., Cuello, C., Hansson, F.G., Jansen, H.J., Dugé De Bernonville, T., Birer Williams, C., Vergès, V., Lezin, E., et al. (2023). The Rauvolfia Tetraphylla Genome Suggests Multiple Distinct Biosynthetic Routes for Yohimbane Monoterpene Indole Alkaloids. Commun. Biol. **6**:1197. https://doi.org/10.1038/s42003-023-05574-8.

Stull, G.W., Schori, M., Soltis, D.E., and Soltis, P.S. (2018). Character Evolution and Missing (Morphological) Data across Asteridae. Am. J. Bot. **105**:470–479. https://doi.org/10.1002/ajb2.1050.

Tavares, H., Whibley, A., Field, D.L., Bradley, D., Couchman, M., Copsey, L., Elleouet, J., Burrus, M., Andalo, C., Li, M., et al. (2018). Selection and Gene Flow Shape Genomic Islands That Control Floral Guides. Proc. Natl. Acad. Sci. USA **115**:11006–11011. https://doi.org/10.1073/pnas.1801832115.

Van Moerkercke, A., Steensma, P., Gariboldi, I., Espoz, J., Purnama, P.C., Schweizer, F., Miettinen, K., Vanden Bossche, R., De Clercq, R., Memelink, J., et al. (2016). The Basic Helix-Loop-Helix Transcription Factor BIS2 Is Essential for Monoterpenoid Indole Alkaloid Production in the Medicinal Plant Catharanthus Roseus. Plant J. **88**:3–12. https://doi.org/10.1111/tpj.13230.

Van Moerkercke, A., Steensma, P., Schweizer, F., Pollier, J., Gariboldi, I., Payne, R., Vanden Bossche, R., Miettinen, K., Espoz, J., Purnama, P.C., et al. (2015). The bHLH Transcription Factor BIS1 Controls the Iridoid Branch of the Monoterpenoid Indole Alkaloid Pathway in Catharanthus Roseus. Proc. Natl. Acad. Sci. USA **112**:8130–8135. https://doi.org/10.1073/pnas.1504951112.

Volk, J., Sarafeddinov, A., Unver, T., Marx, S., Tretzel, J., Zotzel, J., and Warzecha, H. (2019). Two Novel Methylesterases from Olea Europaea Contribute to the Catabolism of Oleoside-Type Secoiridoid

Esters. Planta **250**:2083–2097. https://doi.org/10.1007/s00425-019-03286-0.

Wang, M., Huang, J., Liu, S., Liu, X., Li, R., Luo, J., and Fu, Z. (2022). Improved Assembly and Annotation of the Sesame Genome. DNA Res. **29**:dsac041. https://doi.org/10.1093/dnares/dsac041.

Warnes, G.R., Bolker, B., Bonebakker, L., Gentleman, R., Huber, W., Liaw, A., Lumley, T., Maechler, M., Magnusson, A., Moeller, S., et al. (2009). Gplots: Various R Programming Tools for Plotting Data. R Package Version **2**:1.

Wehrens, R., and Kruisselbrink, J. (2018). "Flexible Self-Organizing Maps in Kohonen 3.0." Articles. J. Stat. Softw. **87**:1–18. https://doi.org/10.18637/jss.v087.i07.

Wickham, H. (2016). Ggplot2: Elegant Graphics for Data Analysis (Springer-Verlag). https://ggplot2.tidyverse.org.

Yang, Y., Li, W., Pang, J., Jiang, L., Qu, X., Pu, X., Zhang, G., and Luo, Y. (2019). Bifunctional Cytochrome P450 Enzymes Involved in Camptothecin Biosynthesis. ACS Chem. Biol. **14**:1091–1096. https://doi.org/10.1021/acschembio.8b01124.

Zhao, D., Hamilton, J.P., Pham, G.M., Crisovan, E., Wiegert-Rininger, K., Vaillancourt, B., DellaPenna, D., and Buell, C.R. (2017). De Novo Genome Assembly of Camptotheca Acuminata, a Natural Source of the Anti-Cancer Compound Camptothecin. GigaScience **6**:1–7. https://doi.org/10.1093/gigascience/gix065.

Zhao, S., Wu, L., Xu, Y., and Nie, Y. (2025). Fe(II) and 2-Oxoglutarate-Dependent Dioxygenases for Natural Product Synthesis: Molecular Insights into Reaction Diversity. Nat. Prod. Rep. **42**:67–92. https://doi.org/10.1039/D4NP00030G.

Plant Communications, Volume 7

Supplemental information

Comparative transcriptomic and co-expression analyses enable the discovery of key enzymes responsible for oleuropein biosynthesis in olive (*Olea europaea*)

Ornella Calderini, Mohamed O. Kamileen, Yoko Nakamura, Sarah Heinicke, Ryan M. Alam, Benke Hong, Yindi Jiang, Alma Gutiérrez-Vences, Fiammetta Alagna, Francesco Paolocci, Maria Cristina Valeri, Edoardo Franco, Soraya Mousavi, Roberto Mariotti, Lorenzo Caputi, Sarah E. O'Connor, and Carlos E. Rodríguez-López

SUPPLEMENTARY FIGURES

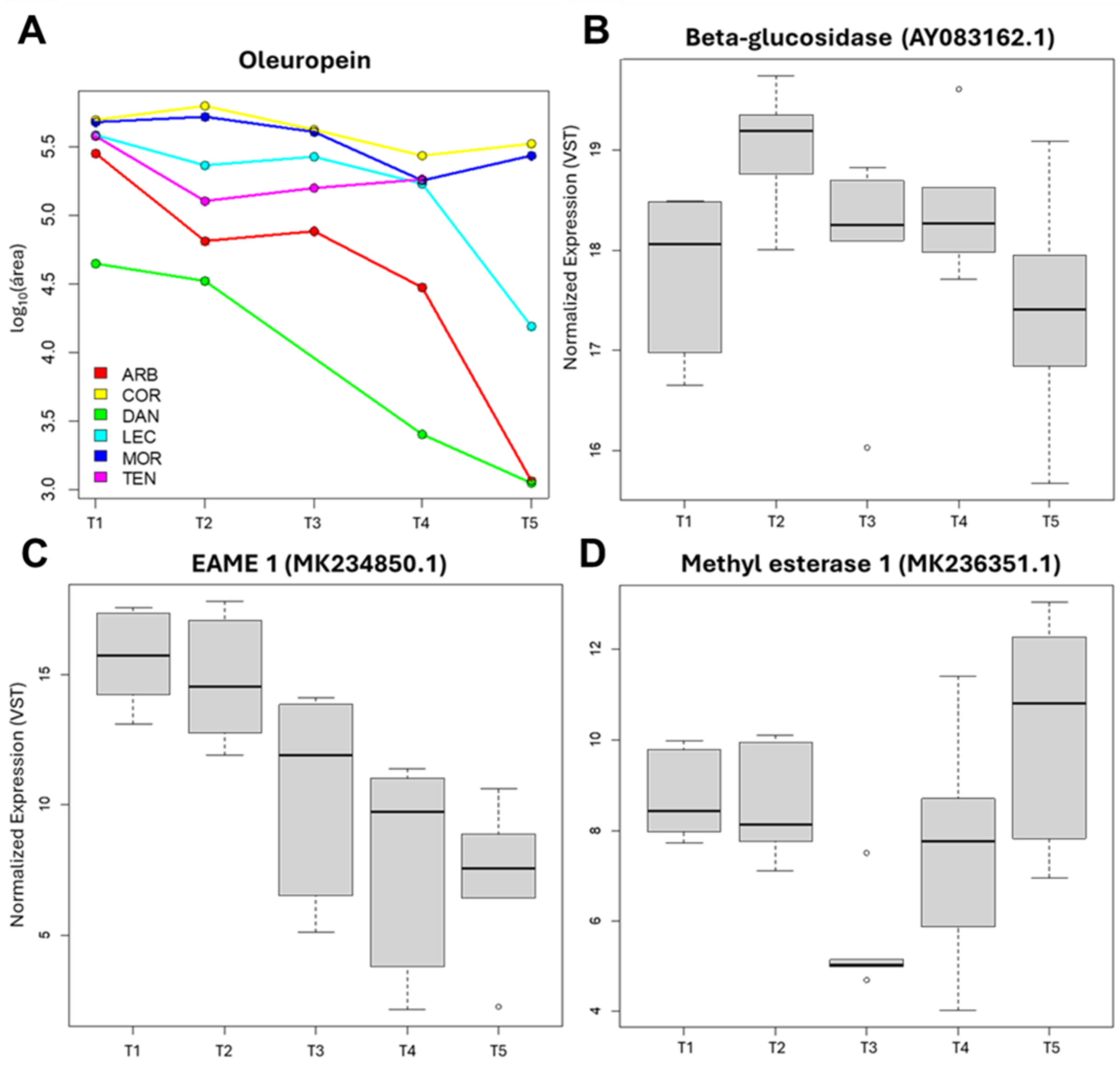

Figure S1. Oleuropein content and putative degradation pathway genes expression through olive maturation. (A) Logarithm of the area under the curve of the extracted ion chromatograms of the most abundant oleuropein adduct ([M-H]$^-$ = 539.1770 ± 0.05) of the cultivars *Dolce d'Andria* (DAN, green), *Tendellone* (TEN, purple), *Arbequina* (ARB, red), *Leccino* (LEC, cyan), *Coratina* (COR, yellow) and *Moraiolo* (MOR, blue). Boxplots depict the normalized mean expression after DESeq2 variance stabilized transformation of **(B)** a beta-glucosidase (GenBank: AY083162.1) reported by Koudounas *et al*. (2015), and **(C)** elenolic acid methylesterase 1 (EAME1, GenBank: MK234850.1) and **(D)** a methyl esterase 1 (GenBank: MK236351.1) reported by Volk *et al*. (2019), presumed to belong to degradation pathway of oleuropein and oleoside methyl ester.

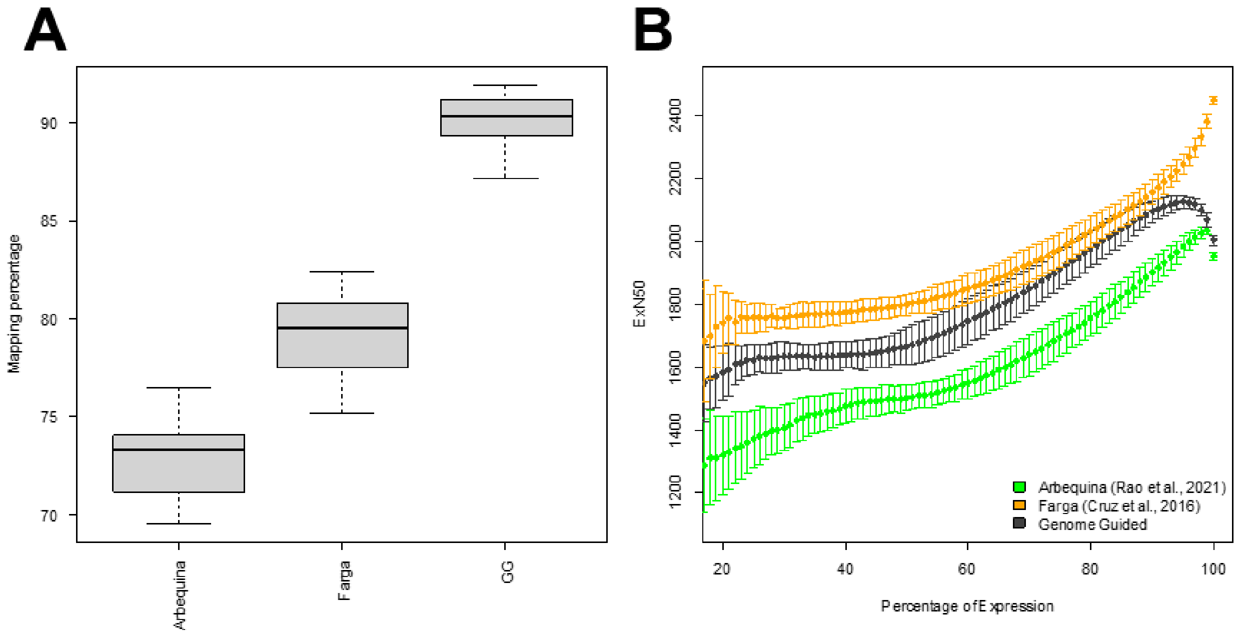

Figure S2. Assembly metrics. (A) Mapping of the sequenced reads to the published Arbequina (Rao *et al.*, 2021) and Farga (Cruz *et al.*, 2016) genomes, as well as the genome guided assembly using Farga genome as a reference (GG.) (B) ExN50 of contigs, i.e. N50 as a function of percentage of expression of the top x-expressed genes of the mappings against Arbequina (green), Farga (yellow) and our genome guided assembly (black), expressed as mean values (solid dots) and standard deviation (error bars.)

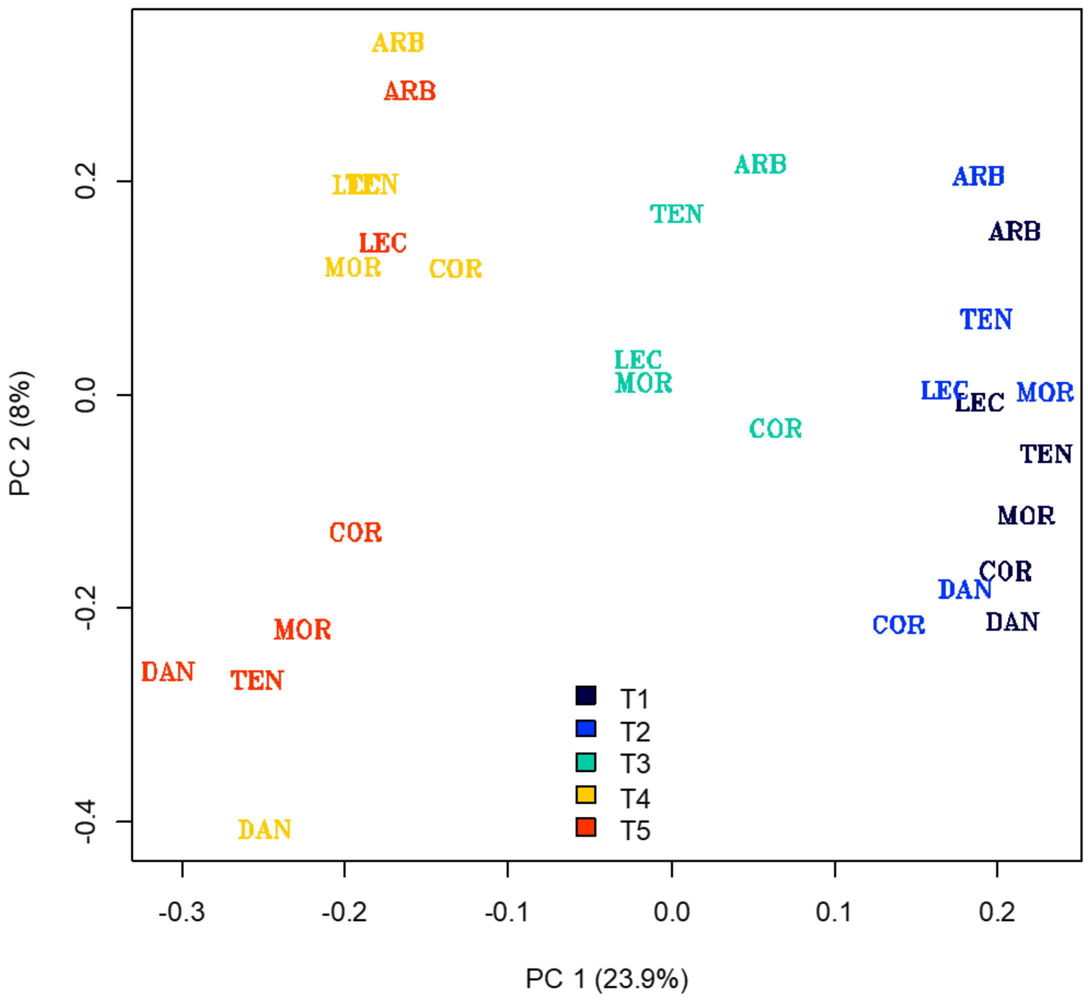

Figure S3. Principal Component Analysis (PCA) of expression data. A loadings plot of the main two components of a PCA of the expression of all genes detected in samples through maturation. Labels correspond to cultivars *Dolce d'Andria* (DAN), *Tendellone* (TEN)*, Arbequina* (ARB), *Leccino* (LEC), *Coratina* (COR) and *Moraiolo* (MOR). Colors correspond to stages 1 (black), 2 (blue), 3 (cyan), 4 (yellow) and 5 (red).

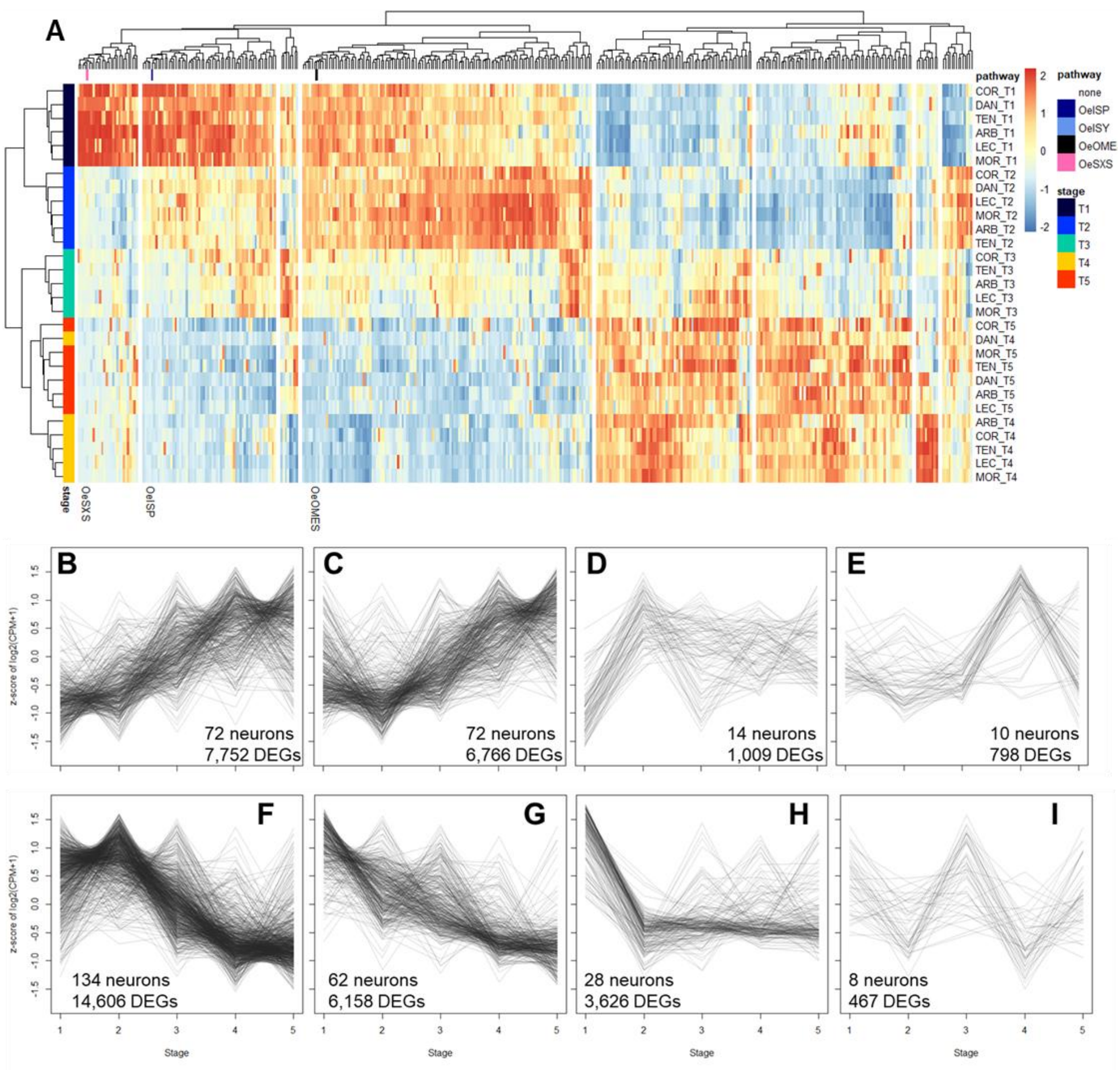

Figure S4. Heatmap of differentially expressed genes during Olive fruit maturation and ripening.
(A) Heatmap of the codebook vectors of the 400 self-organizing map nodes, showing the type expression pattern of the 41,182 differentially expressed genes in olive fruit through maturation. Row band colors correspond to stages 1 (black), 2 (blue), 3 (cyan), 4 (yellow) and 5 (red). **(B-I)** Line plots of the codebook vectors of the 400 self-organizing map nodes, grouped by cluster.

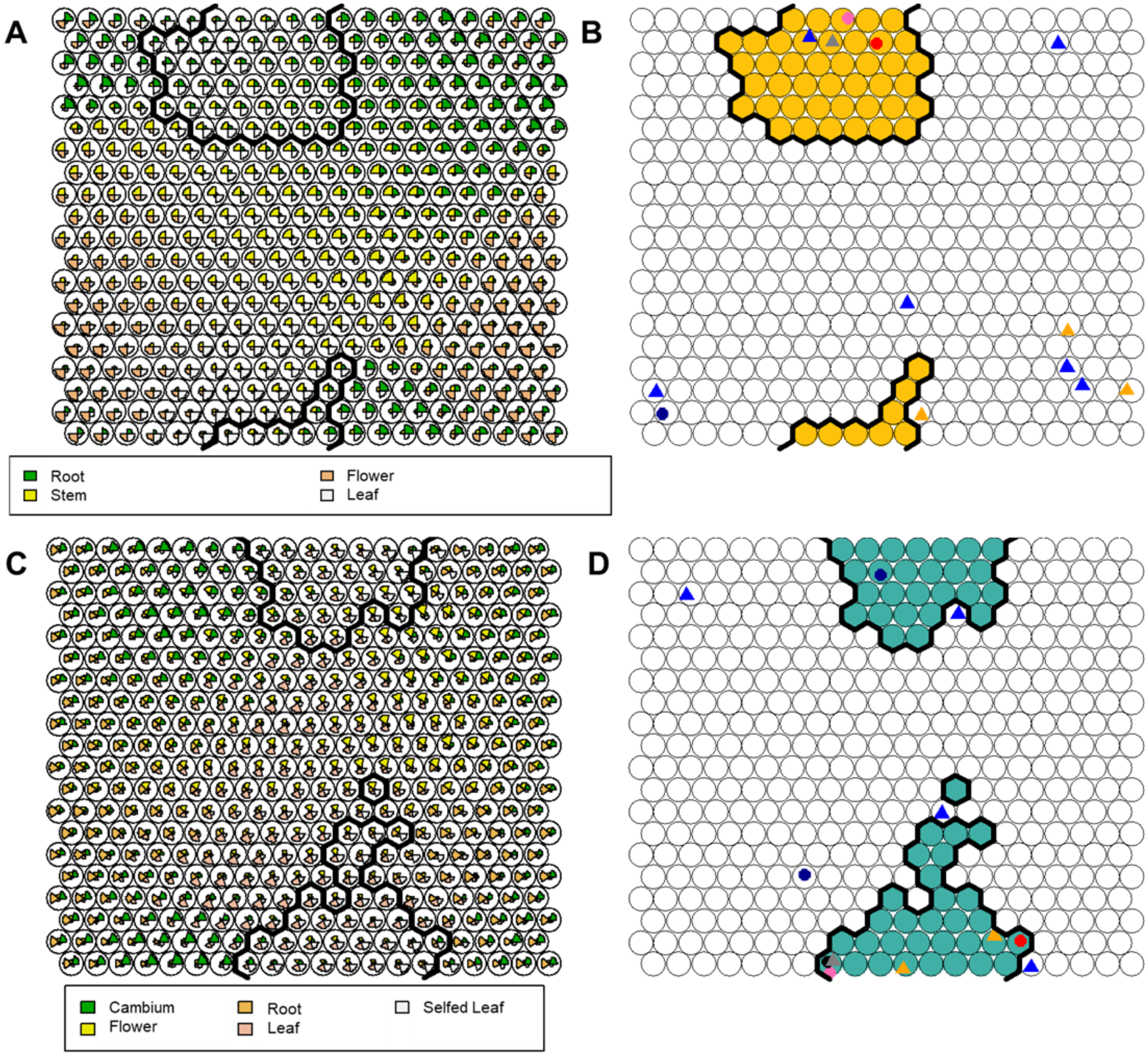

Figure S5. Self-Organizing Maps analysis of *Jasminium sambac* and *Fraxinus excelsior*. Codes plot **(A)** and mapping plots (B) of the self-organizing map (SOM) summarizing expression data of *J. sambac* and codes plot (C) and mapping plots (D) of the SOM for *F. excelsior* expression data. The best BLAST results of known biosynthetic enzymes are shown in the codes plot as figures: ISY, iridoid synthase (black circle); ISP, iridoid synthase paralogue (gray triangle); IO, iridoid oxidase (pink circle); DLGT, 7-deoxyloganetic acid glucosyltransferase (blue triangle); 7eLAMT, 7-*epi*-loganic acid methyl-transferase (dark blue circle); OMES, oleoside methyl ester synthase (red circle); OMEGT, oleoside-11-methyl ester glucosyl transferase (orange triangle). The selected cluster where most biosynthetic genes are located is highlighted in yellow for *J. sambac* and in green for *F. excelsior*.

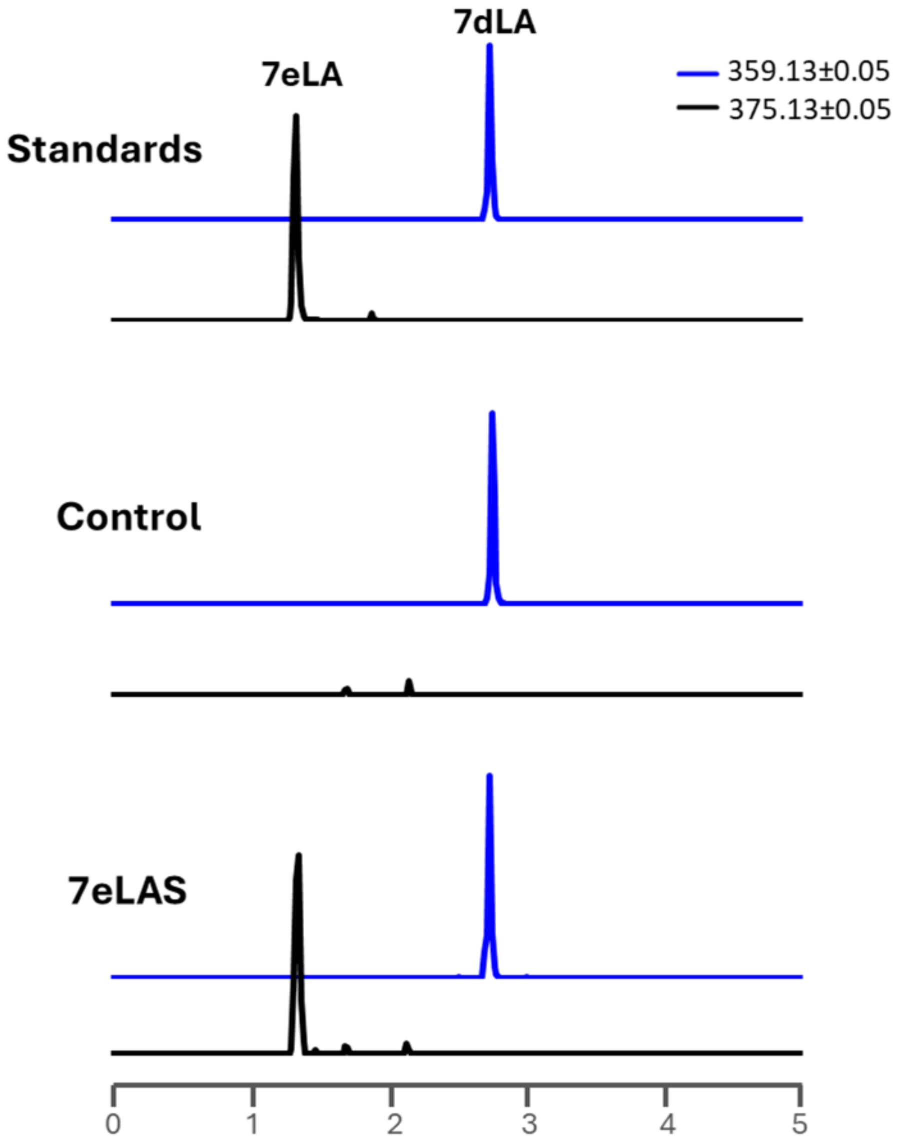

Figure S6. In vitro enzyme assay for Oe7eLAS. Extracted ion chromatogram (XIC) of the most abundant adducts of 7-*epi*-loganic acid (7eLA, $[M-H]^-$) in black (375.1297±0.05) and 7-deoxy-loganic acid (7dLA, $[M-H]^-$) in blue (359.1348 ± 0.05). From top to bottom: mix of standards (Standards), and incubations of 7-deoxy-loganic acid with boiled protein (Control) and purified protein (7eLAS), heterologously produced in *E. coli*

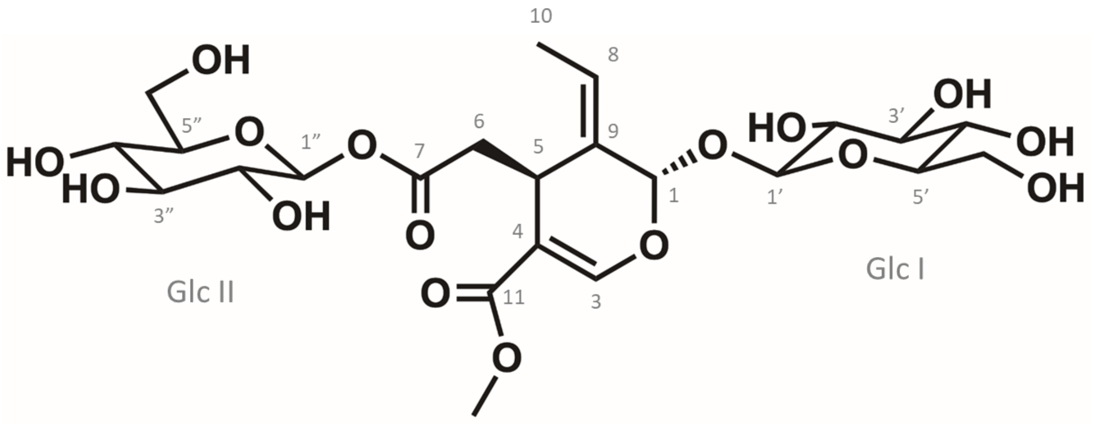

pos.	δ_H	mult.	J_{HH}	δ_C
1	5.94	*dd*	1.3/1.1	95.2
3	7.53	*s*	-	155.2
4	-	-	-	109.0
5	4.01	*dd*	9.2/3.7	31.1
6a	2.79	*dd*	15.4/3.7	40.4
6b	2.59	*dd*	15.4/9.2	40.4
7	-	-	-	171.8
8	6.11	*qd*	7.1/1.1	125.3
9	-	-	-	130.0
10	1.77	*dd*	7.1/1.3	13.7
11	-	-	-	168.6
OMe	3.71	*s*	-	51.8
**Glc I**				
1'	4.81	*d*	7.9	100.7
2'	3.31	*m**	-	74.7
3'	3.41	*dd*	8.7/8.7	78.0
4'	3.31	*m**	-	71.5
5'	3.32	*m**	-	78.3
6'a	3.90	*bd*	12.0	62.7
6'b	3.67	*dd*	12.0/5.9	62.7
**Glc II**				
1'	5.43	*d*	8.2	95.7
2'	3.33	*dd*	8.7/8.2	73.8
3'	3.41	*dd*	9.1/8.7	78.0
4'	3.35	*m**	-	71.0
5'	3.35	*m**	-	78.6
6'a	3.82	*bd*	12.3	62.3
6'b	3.68	*bd*	12.3	62.3
* overlapped signals J unresolved				

Figure S7. Chemical shift data for 7-β-1-D-glucopyranosyl oleoside-11-methyl ester. Chemical shifts table (right) acquired at 700MHz in MeOH-d_3 is shown along with the elucidated structure.

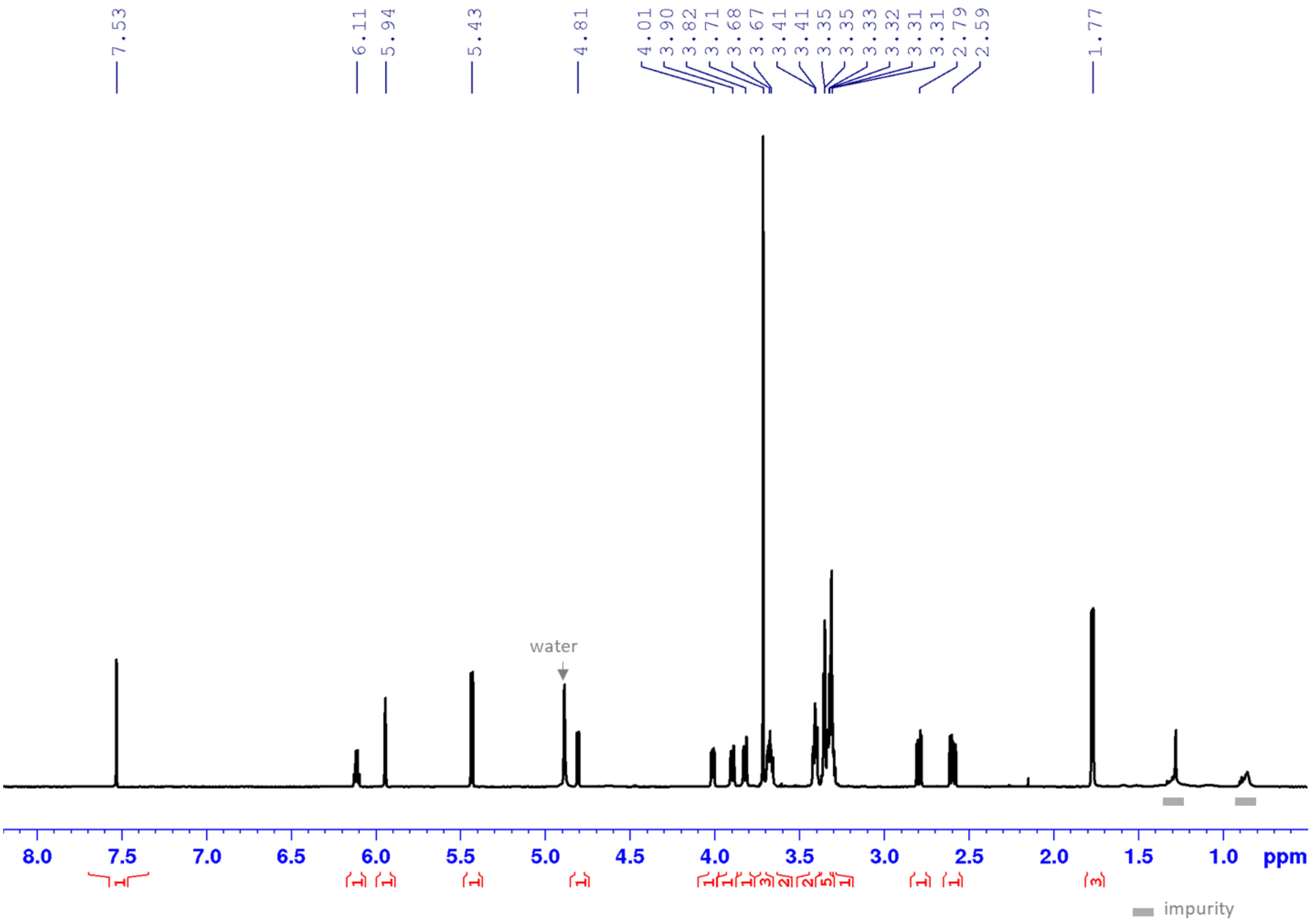

Figure S8. Proton NMR spectra for 7-β-1-D-glucopyranosyl oleoside-11-methyl ester with water suppression. Full range in MeOH-*d*3 is shown.

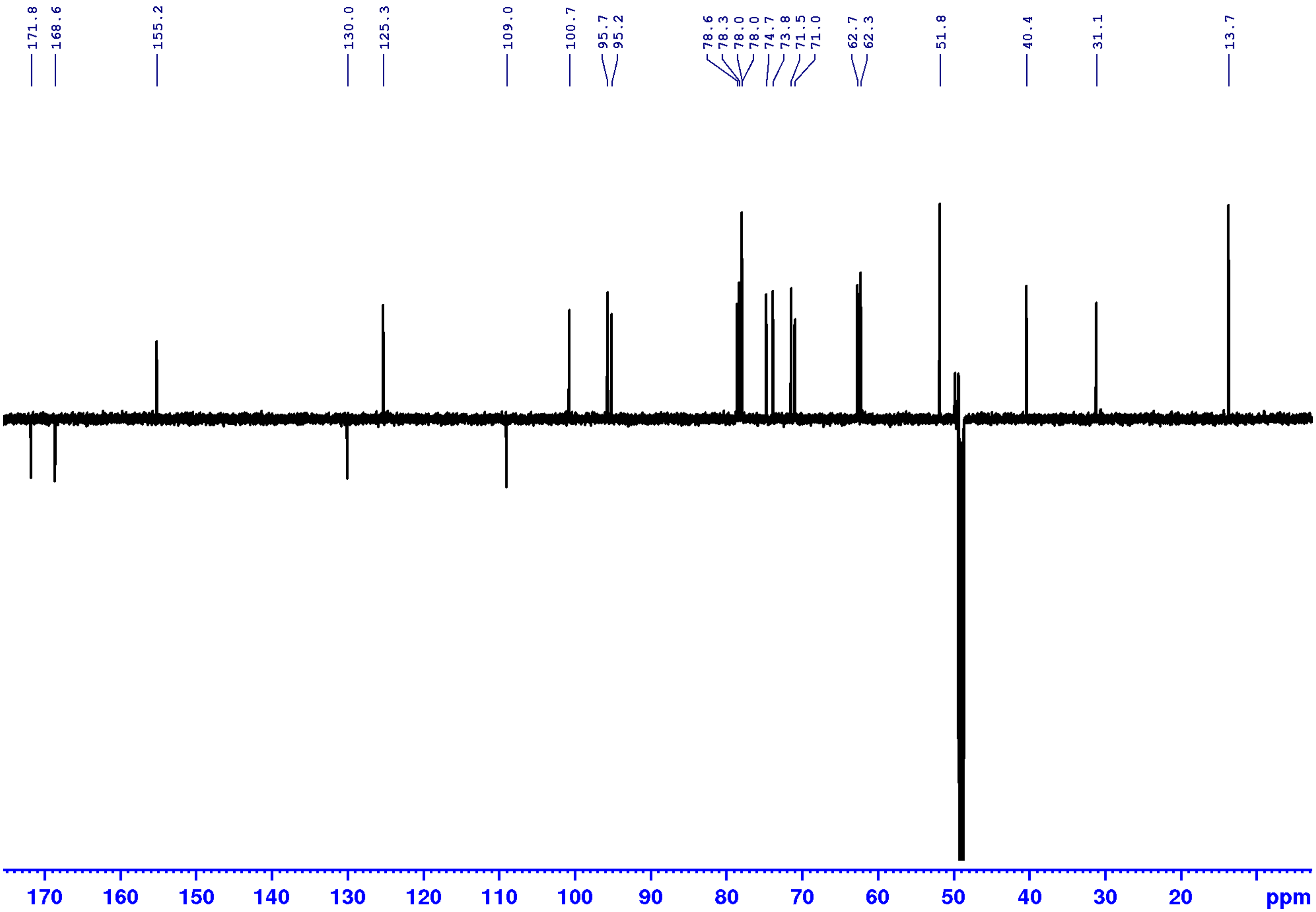

Figure S9. DEPTQ spectra for 7-β-1-D-glucopyranosyl oleoside-11-methyl ester.

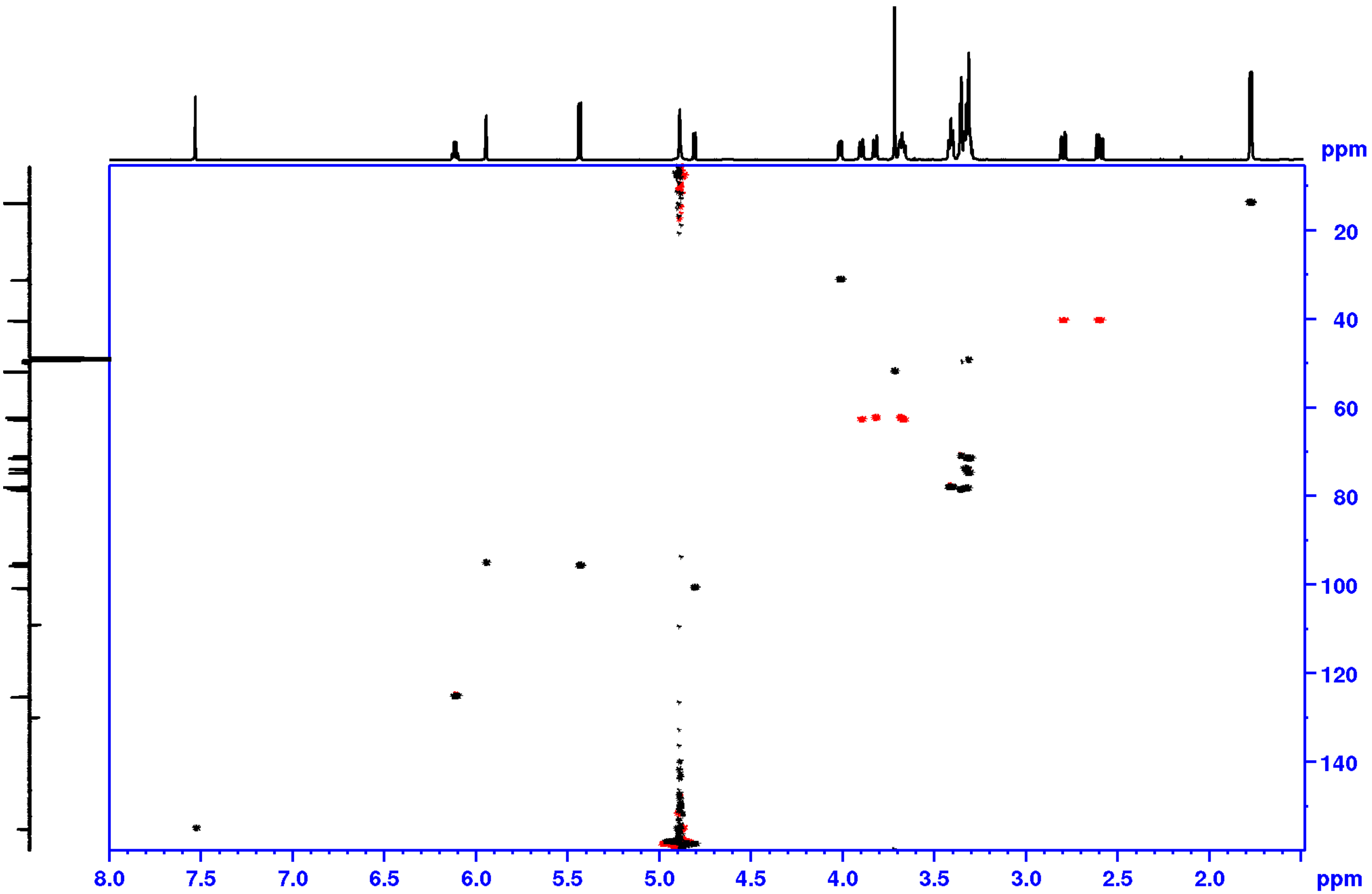

Figure S10. Full range phase sensitive HSQC spectra for 7-β-1-D-glucopyranosyl oleoside-11-methyl ester. CH/CH_3: black, CH_2: red.

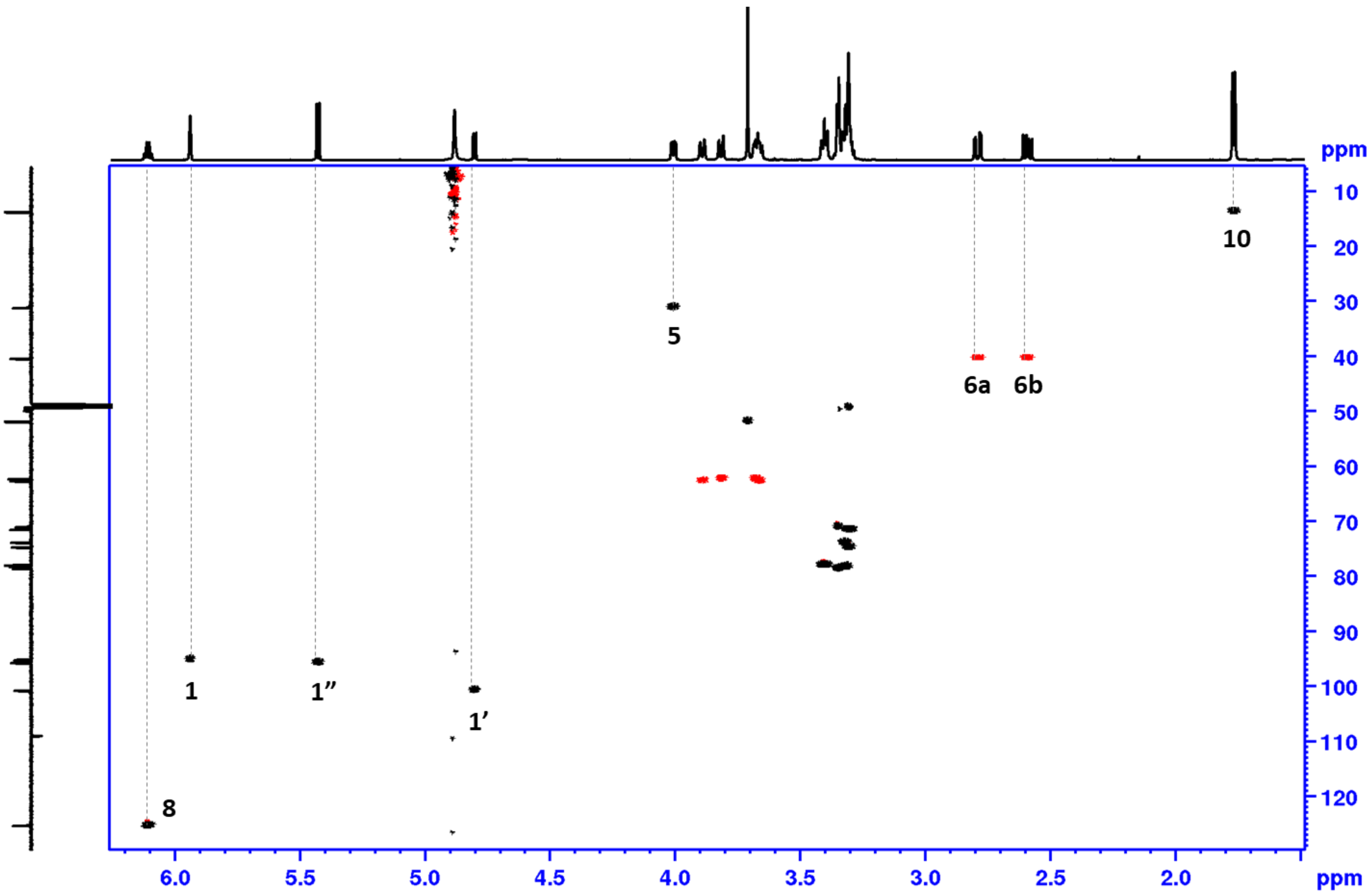

Figure S11. Focused phase sensitive HSQC spectra for 7-β-1-D-glucopyranosyl oleoside-11-methyl ester, from 1.5-6.5 ppm range. CH/CH_3: black, CH_2: red.

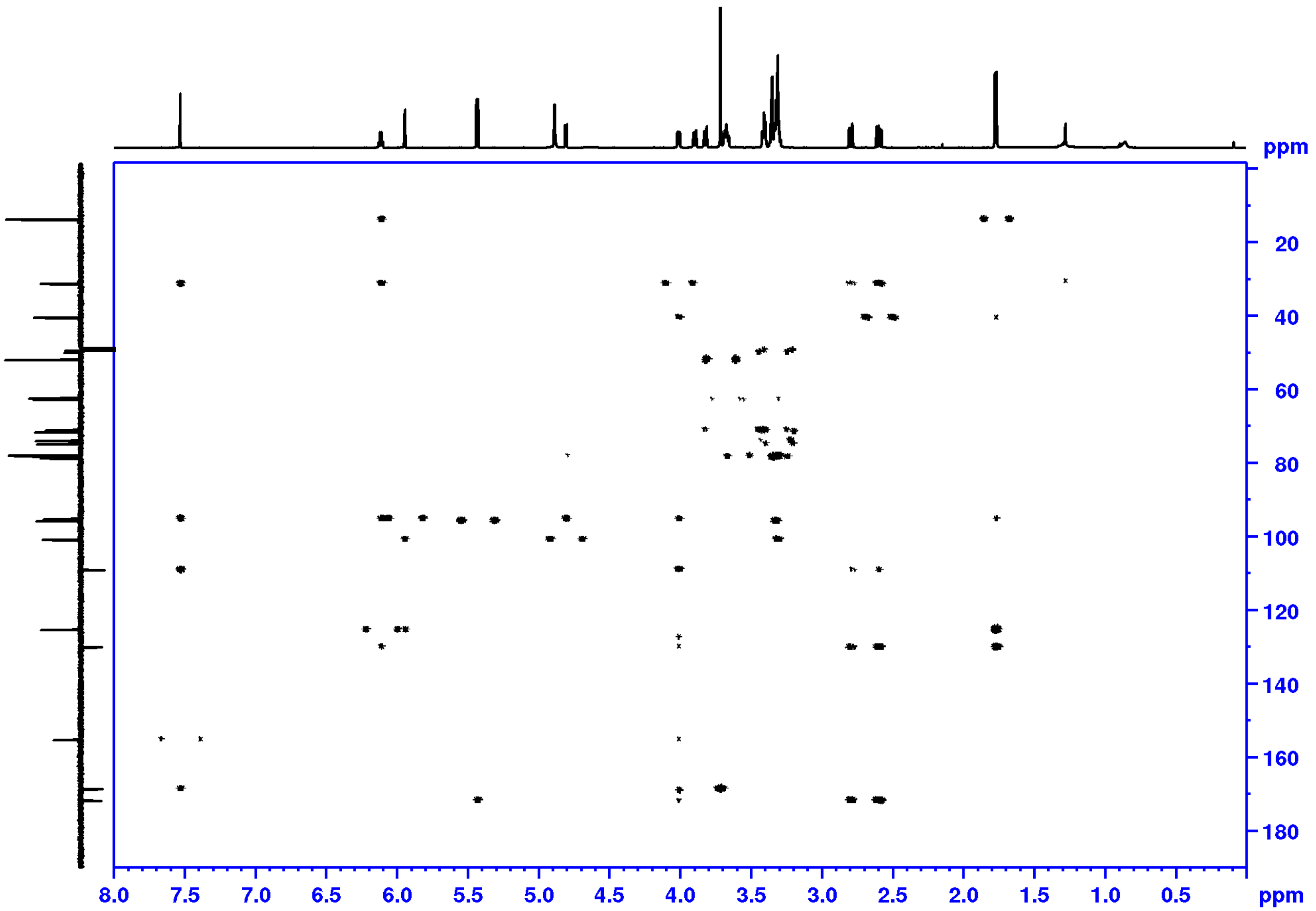

Figure S12. HMBC full range spectra for 7-β-1-D-glucopyranosyl oleoside-11-methyl ester in MeOH-*d*$_3$.

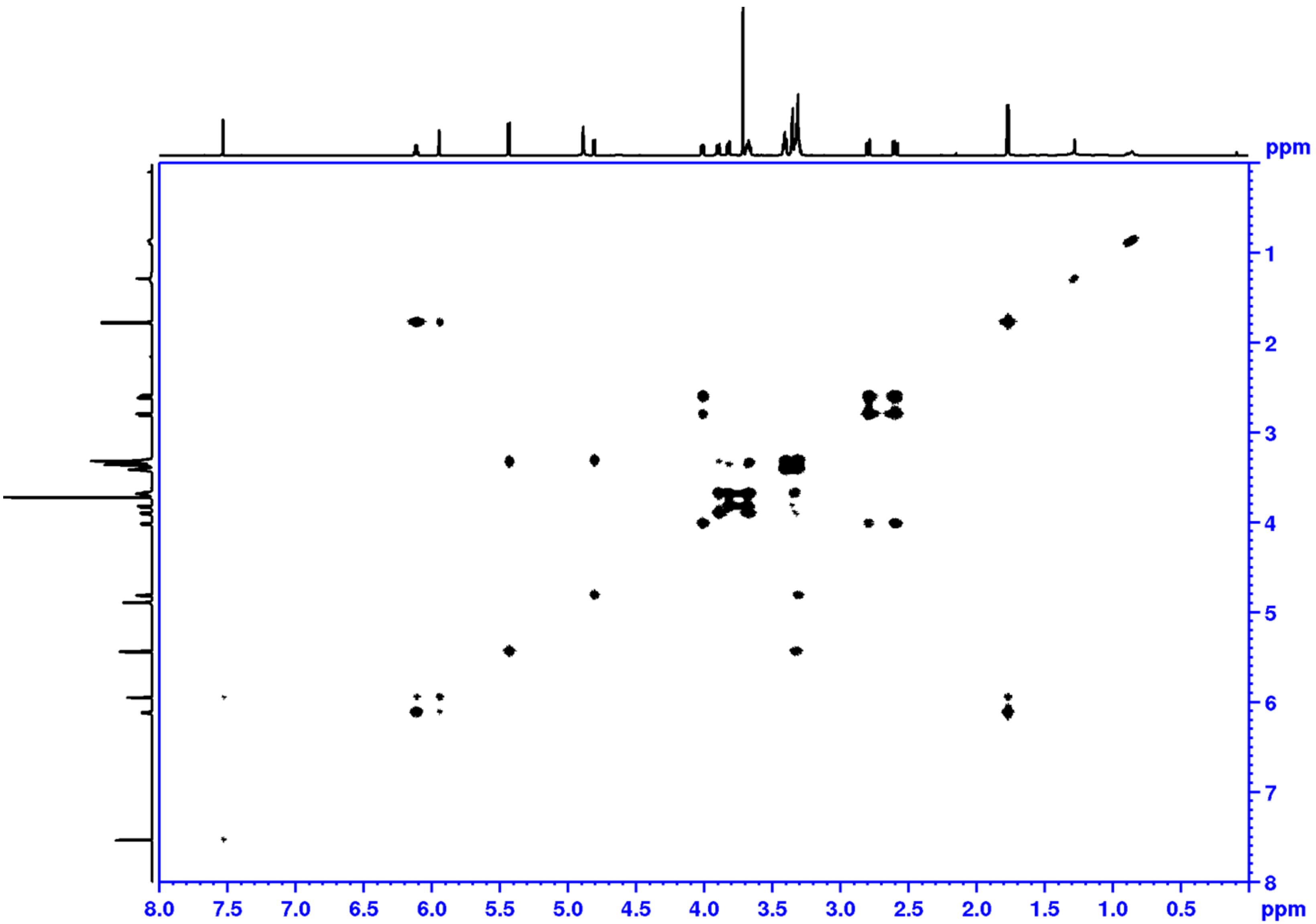

Figure S13. COSY full range spectra with water suppression for 7-β-1-D-glucopyranosyl oleoside-11-methyl ester in MeOH-*d*$_3$

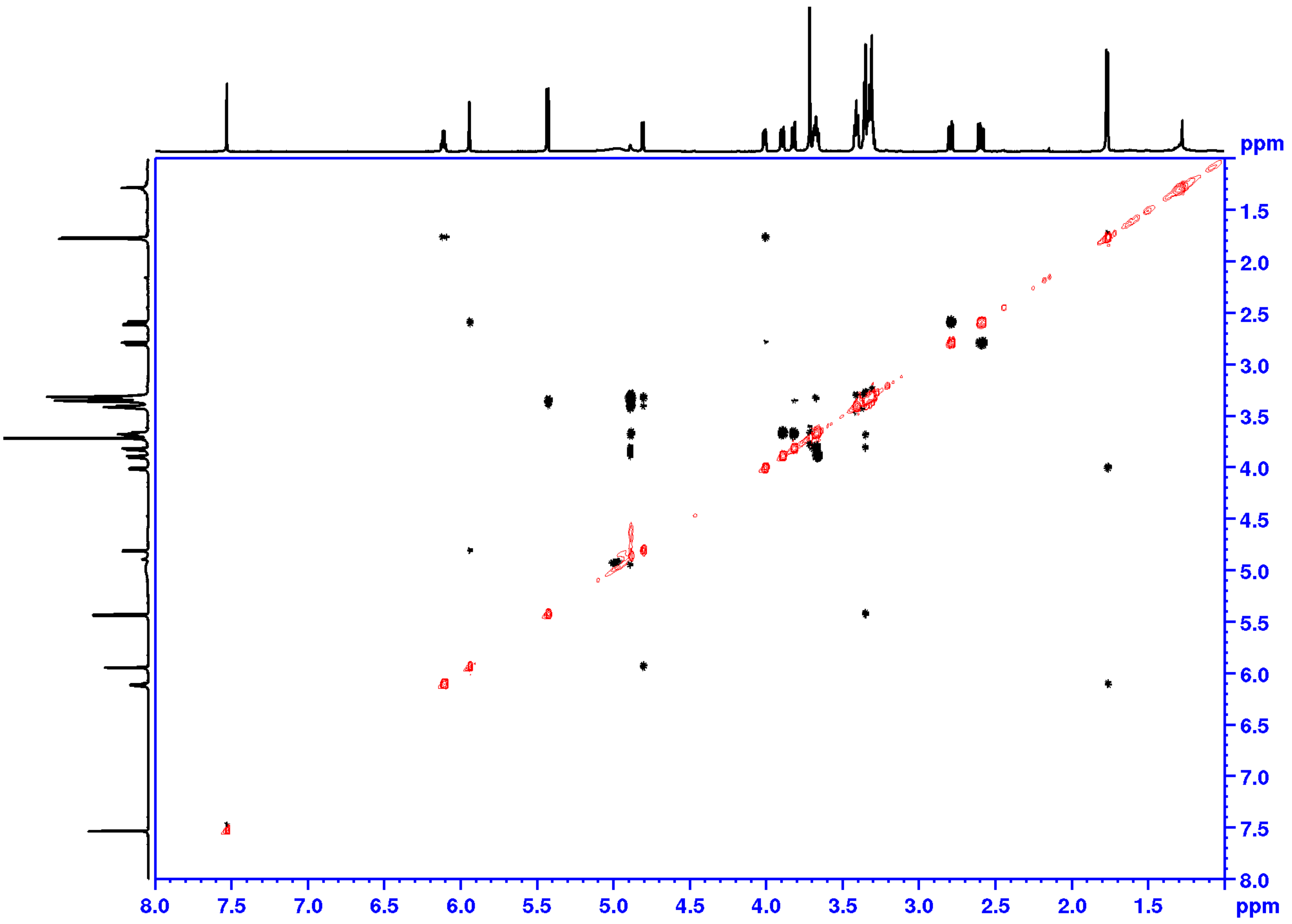

Figure S14. ROESY spectra for 7-β-1-D-glucopyranosyl oleoside-11-methyl ester, with water suppression.

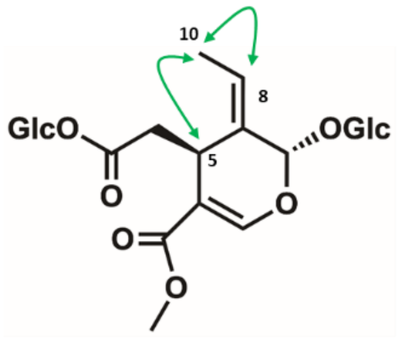

Green arrow shows important ROESY correlations

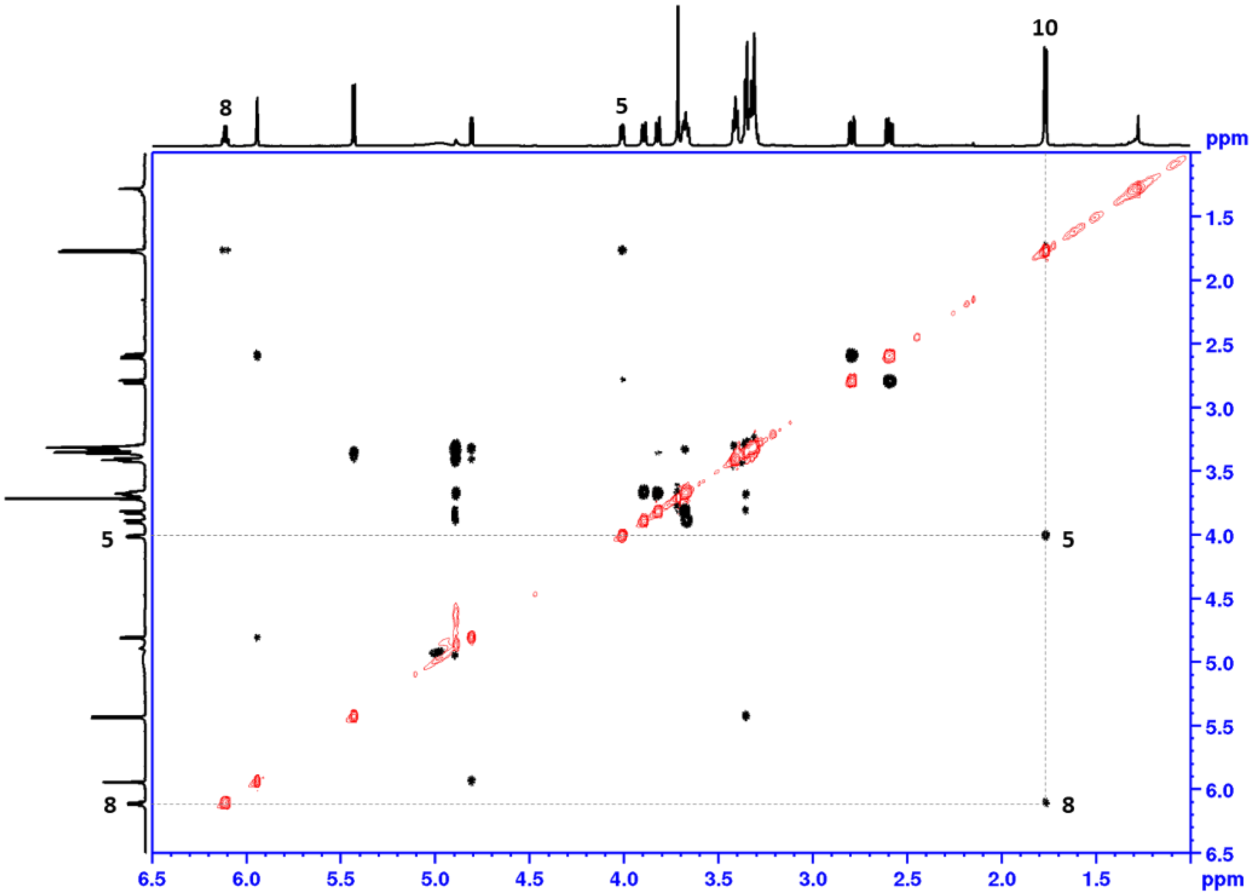

Figure S15. Focused ROESY spectra for 7-β-1-D-glucopyranosyl oleoside-11-methyl ester, with water suppression; range from 1.0-6.5 ppm.

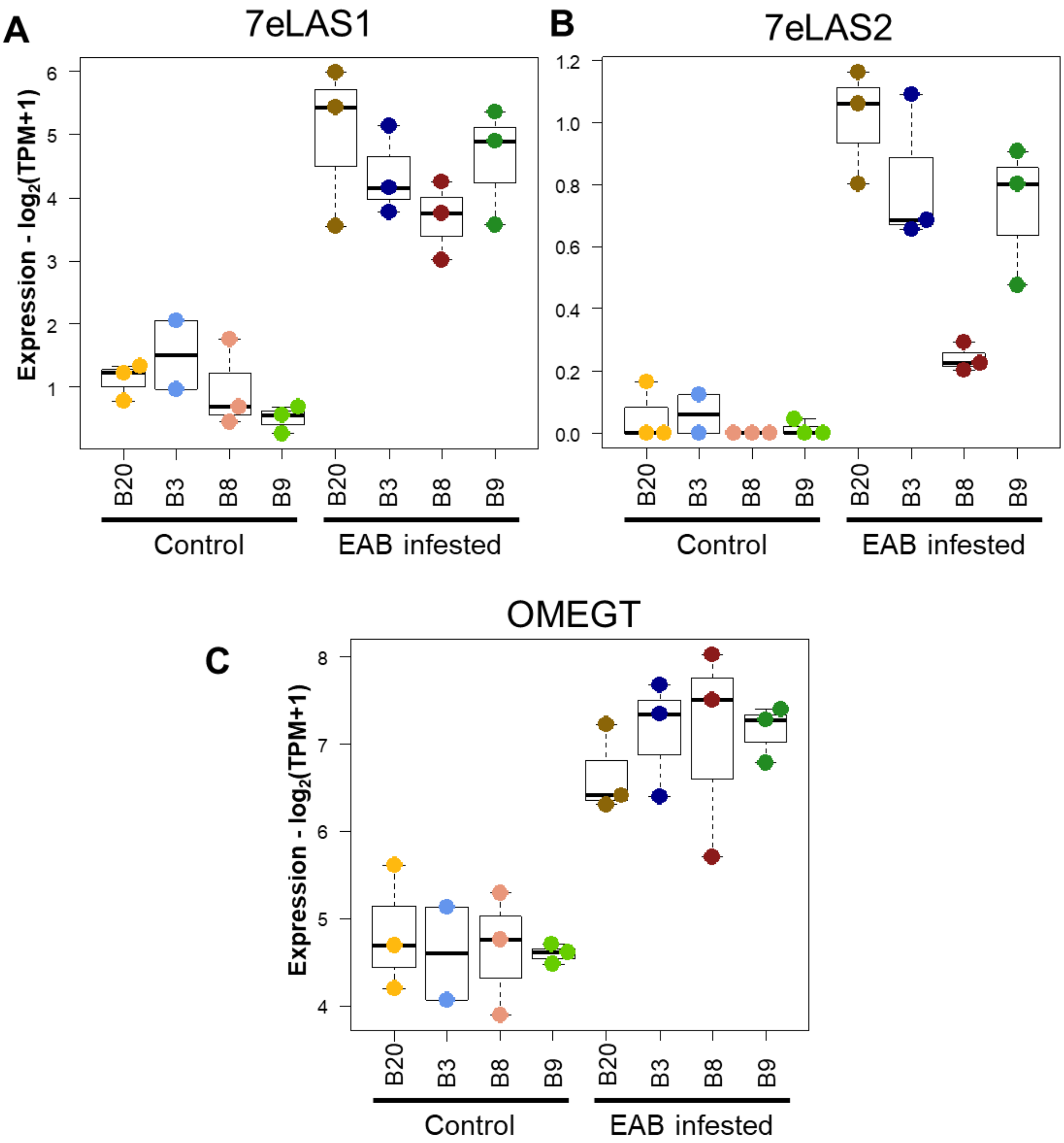

Figure S16. Response of *Fraxinus excelsior* cambium to an Emerald Ash Borer challenge. Expression is shown as a boxplot, with overlaid points for each individual (n=3 per combination) of B3 (cyan/blue), B8 (pink/red), B9 (green/dark green) and B20 (yellow/brown) genotypes infested with Emerald Ash Borer (right) and healthy (left.) RNA-seq data was obtained from project PRJDB15336 (Doonan et al., 2023), mapped against *F. excelsior* genome-guided transcriptome; only statistically significant ($p<0.05$) genes, via two-way ANOVA, are shown.

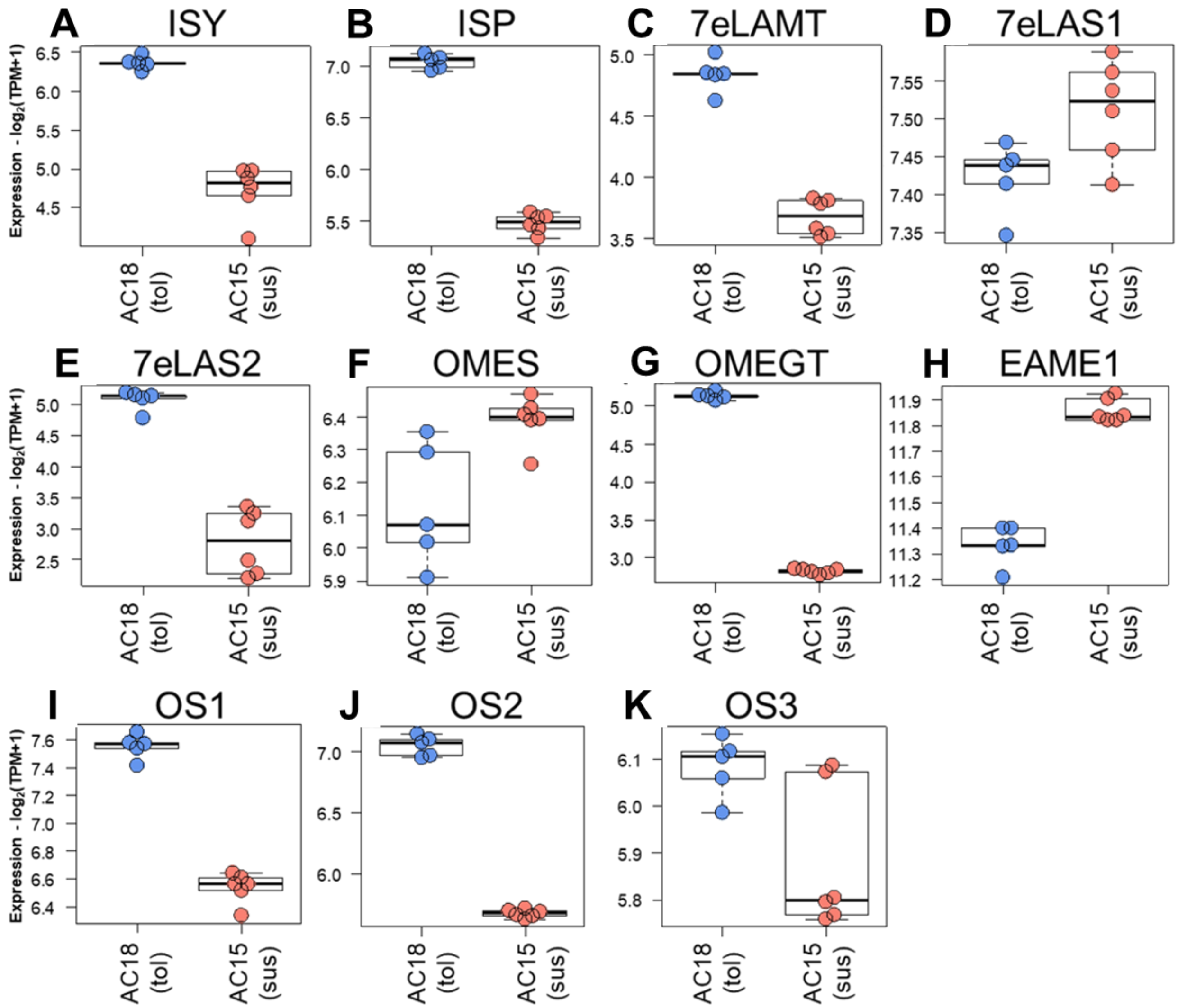

Figure S17. Response of oleaster cultivars AC15 and AC18 to infection by *Verticillium dahliae*. Expression is shown as a boxplot, with overlaid points for each individual of the tolerant cultivar AC18 (n=5) and the susceptible cultivar AC15 (n=6). RNA-seq data was obtained from project PRJNA1030405 (Mascuñano et al., 2025), mapped against the published gene models from the Farga genome (Cruz et al., 2016); only statistically significant ($p<0.05$) genes, via ANOVA, are shown.

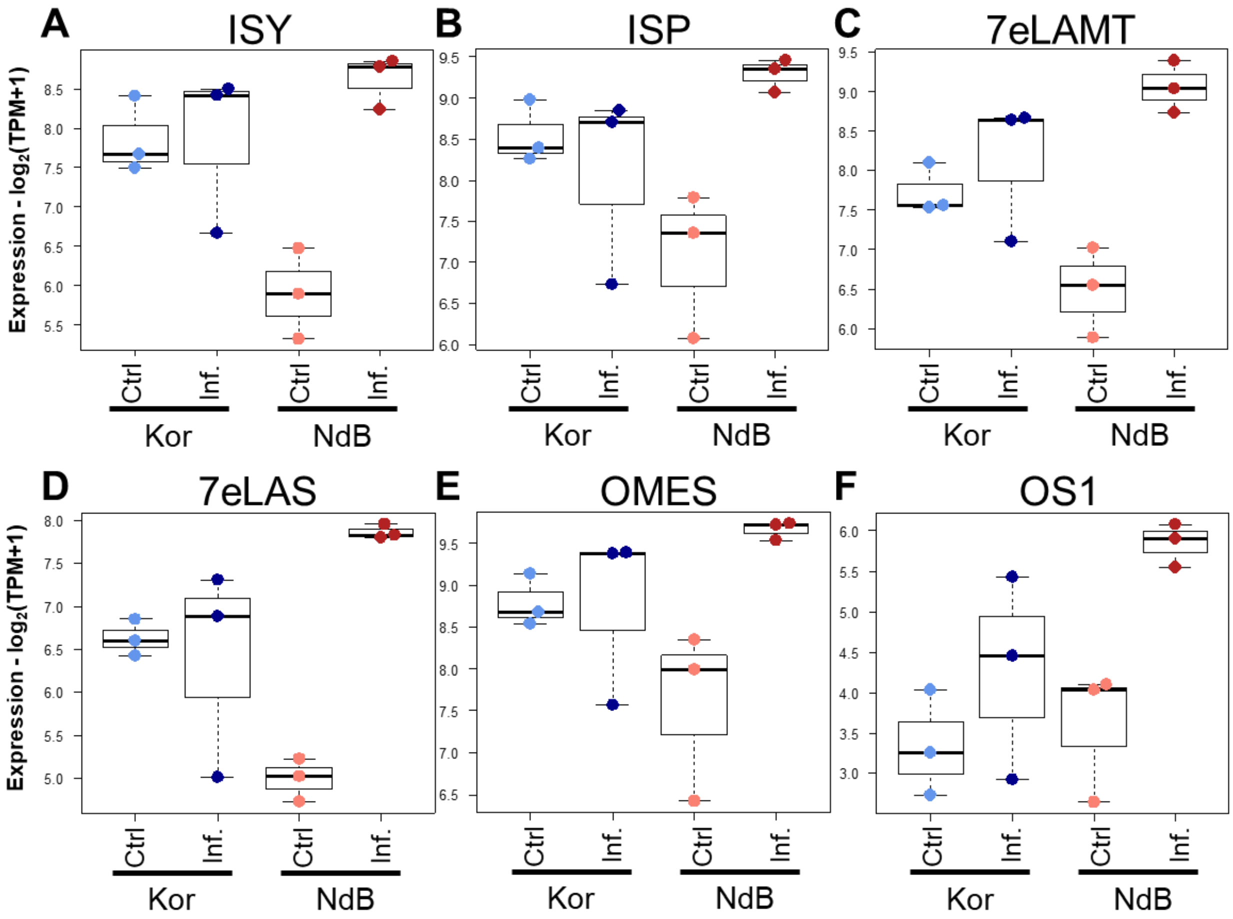

Figure S18. Differential response of olive cultivars Koroneiki and Nocellara del Belice to infection of *Spilocea oleagina*. Expression is shown as a boxplot, with overlaid points for each individual (n=3 per combination) of Koroneiki (Kor; left), a low susceptibility cultivar, and Norcellara del Belice (NdB; left) a highly susceptible cultivar, infected with *Spilocea oleagina* (Inf.) or healthy (Ctrl.) RNA-seq data was obtained from project PRJNA929711 (Marchese *et al*., 2023), mapped against the published gene models from the Farga genome (Cruz et al., 2016); only statistically significant ($p<0.05$) genes, via two way ANOVA, are shown.

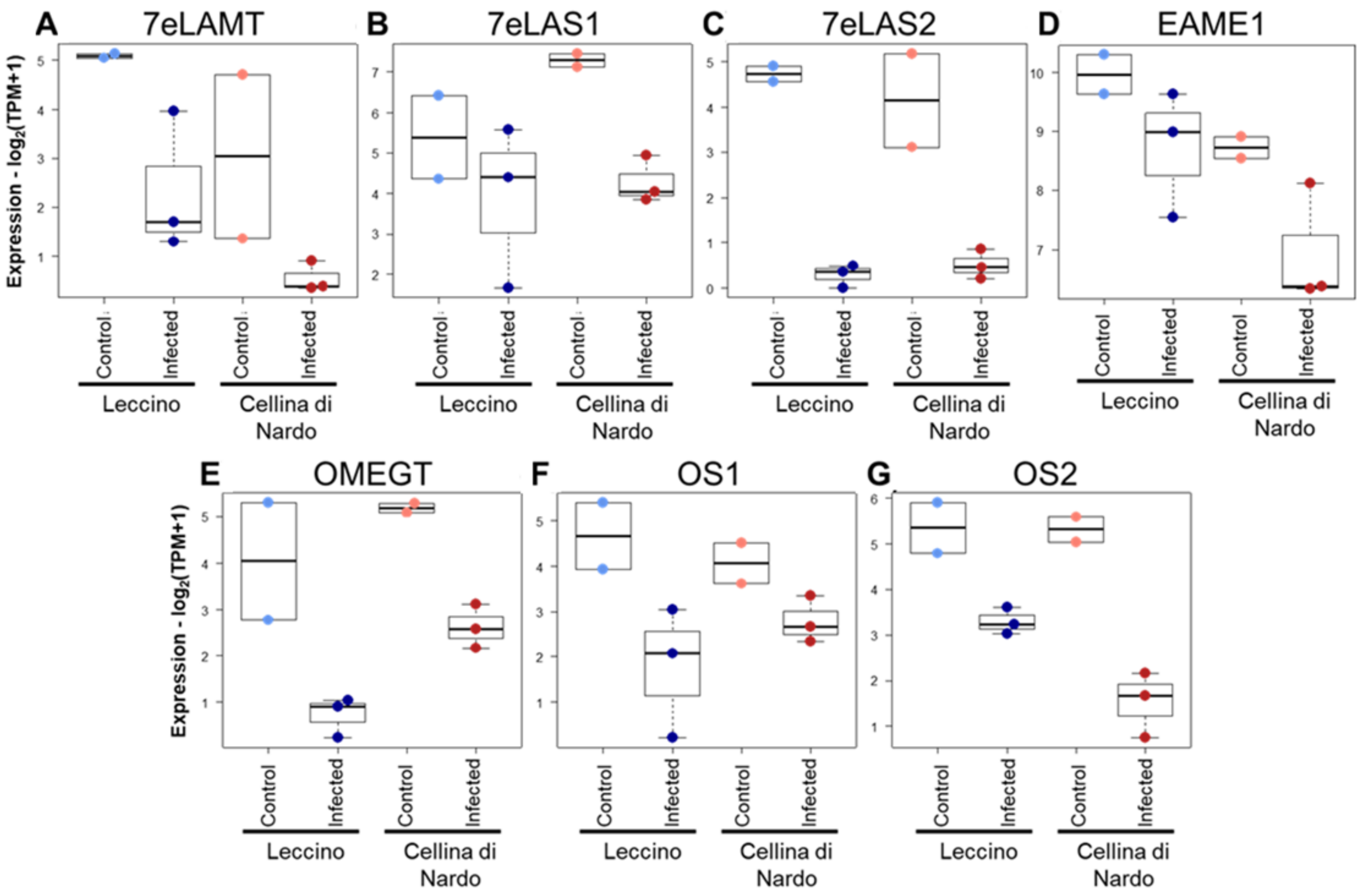

Figure S19. Response of olive cultivars to infection by *Xylella fastidiosa*. Expression is shown as a boxplot, with overlaid points for each individual of Leccino and Cellina di Nardo cultivars, infected with *Xylella fastidiosa* (n=3 per cultivar) or healthy (n=2 per cultivar.) RNA-seq data was obtained from project PRJNA780294 mapped against the published gene models from the Farga genome (Cruz et al., 2016); only statistically significant ($p<0.05$) genes, via two-way ANOVA, are shown.

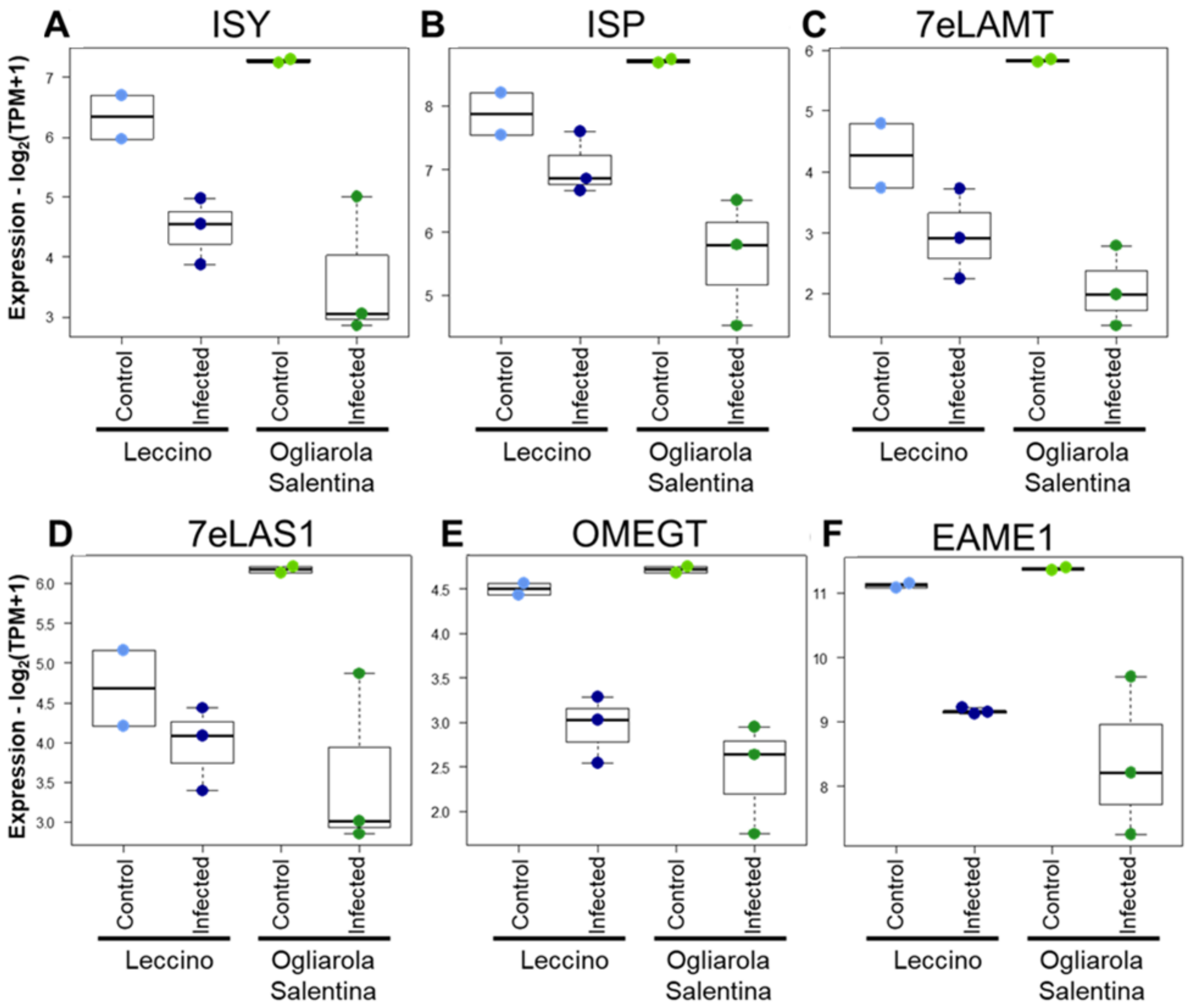

Figure S20. Response of olive cultivars to infection by *Xylella fastidiosa*. Expression is shown as a boxplot, with overlaid points for each individual of Leccino and Ogliarola Salentina cultivars, infected with *Xylella fastidiosa* (n=3 per cultivar) or healthy (n=2 per cultivar.) RNA-seq data was obtained from project PRJNA316374 (Giampetruzzi et al., 2016) mapped against the published gene models from the Farga genome (Cruz et al., 2016); only statistically significant ($p<0.05$) genes, via two-way ANOVA, are shown.
